# *Salmonella* Typhimurium hijacks a host glucose transporter for intravacuolar proliferation

**DOI:** 10.1186/s12964-026-02981-2

**Published:** 2026-06-05

**Authors:** Xianglan Fang, Liuliu Shi, Yan Ding, Youwei Wang, Wei Chen, Juan Xue, Long Wang, Yuanjian Hui, Xinyuan Tao, Wanmo Xiao, Jin Yang, Lijie Du, Zheng Cao, Duoshuang Xie, Kun Meng

**Affiliations:** 1 Department of Infection Prevention and Control, School of Public Health Institute of Infection and Immunity, Hubei Provincial Clinical Research Center of Central Nervous System Repair and Functional Reconstruction, Affiliated Taihe Hospital, Hubei University of Medicine, Shiyan, Hubei 442000 China; 2 School of Basic Medicine, Hubei Provincial Clinical Research Center for Umbilical Cord Blood Hematopoietic Stem Cells, Hubei Key Laboratory of Embryonic Stem Cell Research, Hubei University of Medicine, Shiyan, 442000 China; 3 Center for Evidence-Based Medicine and Clinical Research, Taihe Hospital, Hubei University of Medicine, Shiyan, 442000 China; 4 Life Science College, Wuhan University, Wuhan, 430072 China; 5 Department of Cardiology, Taihe Hospital, Hubei University of Medicine, Shiyan, Hubei 442000 China

**Keywords:** Intravacuolar bacterial, Glut1, Glucose metabolism, Anti-bacterial immunity

## Abstract

*Salmonella* is an intracellular pathogen that can reside within a vacuole, which protects it from cytosolic host defenses at the expense of limited nutrient access. Glucose serves as a critical carbon source supporting *Salmonella*'s intracellular replication. However, the molecular mechanisms driving glucose uptake of host cells and the pathways by which cytosolic glucose becomes accessible to intravacuolar *Salmonella* remain poorly understood. Here, we elucidate a three-pronged strategy through which *Salmonella* Typhimurium (*S.* Typhimurium) exploits Glut1 to co-opt host glucose metabolism for pathogenic advantage. Firstly, *S.* Typhimurium infection upregulates the glucose transporter Glut1 by activating the MAPK signaling cascade, enhancing host glucose uptake, and accelerating glycolytic flux. Secondly, *S.* Typhimurium utilizes Glut1 to the bacterial vacuolar membrane to establish a glucose-import conduit that facilitates bacterial acquisition of cytosolic glucose. Thirdly, K29-linked ubiquitination on bacterial vacuolar membranes is a previously unrecognized regulatory mechanism that potentiates Glut1 transporter activity. Inhibition of Glut1 potentiates *S.* Typhimurium-triggered innate immune responses and attenuates bacterial virulence in vitro and in vivo. Collectively, these findings delineate a novel paradigm of metabolic hijacking, wherein *S.* Typhimurium systematically rewires host glucose metabolic networks to support intracellular proliferation, providing new insights into host-directed antimicrobial interventions.

**Supplementary Information:**

The online version contains supplementary material available at 10.1186/s12964-026-02981-2.

## Introduction

*Salmonella enterica* is a Gram-negative facultative intracellular pathogen capable of infecting a broad range of hosts, causing diseases ranging from self-limiting gastroenteritis to systemic infections [1, 2]. During infection, *Salmonella* can occupy diverse intracellular niches depending on the cell type infected. One well-characterized niche is the *Salmonella*-containing vacuole (SCV), a compartment formed through interactions with the host endolysosomal system and the subsequent formation of *Salmonella*-induced filaments (SIFs). These membrane structures enable immune evasion but confine the pathogen within a membrane-bound environment [3–6]. Consequently, *Salmonella* needs to develop unique strategies to reprogram host metabolism and exploit available host metabolites to meet the bacterial energetic demands [7, 8]. It has been reported that *Salmonella* simultaneously utilizes multiple host-derived nutrients, including glucose, glycerol, fatty acids, and GlcNAc, to support intracellular proliferation and systemic infection [9]. While glucose serves as a primary carbon source for intracellular replication by boosting glycolysis [10–13], a recent study showed that *Salmonella* can achieve efficient intracellular replication independent of host glycolysis when other preferred carbon sources (such as glycerol and galactose) are sufficiently available [14]. Thus, the metabolic arms race between intracellular *Salmonella* and its host dictates the outcome of infection.

When macrophages recognize microbial ligands, their glucose metabolism undergoes a characteristic switch from oxidative phosphorylation (OXPHOS) to aerobic glycolysis. This well-defined metabolic transition in activated macrophages is commonly termed the “Warburg effect” [15]. *S*. Typhimurium infection of murine macrophages has been shown to trigger a comparable Warburg-like metabolic phenotype in host cells [13]. Although the host's glycolytic shift presents potential risks to *Salmonella* by promoting aldolase A-dependent v-ATPase assembly and consequent phagosome acidification [16], this metabolic reprogramming is generally recognized as beneficial and often essential for successful infection [10, 11, 13, 17, 18]. Increased glucose uptake and glycolytic flux may dampen some fatal immune responses. For instance, *Salmonella* redirects metabolism toward glycolysis as an antioxidant defense by mitigating trans-membrane electron flow to preserve the proton motive force (ΔpH) [19]. This metabolic shift also lowers the NAD⁺/NADH ratio, which in turn orchestrates divergent host antimicrobial immune responses [20]. Concurrently, this shift augments the availability of glucose and glycolytic intermediates to fuel bacterial energy metabolism and virulence [11]. A well-characterized paradigm involves the SPI-1 effector SopE2, which promotes the accumulation of 3-phosphoglycerate (3PG), pyruvate, and lactate. Among these, 3PG is a direct nutrient source for intracellular proliferation, while pyruvate and lactate act as signaling molecules to activate SPI-2 virulence gene expression [13]. Despite these insights, the molecular mechanisms underlying infection-induced glycolytic enhancement remain poorly defined.

Glucose transporter 1 (Glut1), as the primary facilitative glucose transporter in mammalian cells, catalyzes the rate-limiting initial step of cellular glucose uptake and thus occupies a central position in host carbohydrate metabolism and energy homeostasis [21, 22]. Recent multi-omics investigations have consistently observed that *Salmonella* infection induces significant upregulation of Glut1 expression [11, 13, 20], suggesting that the transporter may serve as a critical interface in host–pathogen metabolic interactions. However, the molecular mechanisms underlying this regulation, and the functional role of Glut1 during *S*. Typhimurium infection, particularly in supporting intravacuolar bacterial survival and modulating immune responses, remain poorly understood.

In this study, we demonstrate that *S*. Typhimurium infection upregulates the glucose transporter Glut1 through activation of the mitogen-activated protein kinase (MAPK) signaling cascade in murine macrophages, leading to enhanced host cellular glucose uptake and accelerated glycolytic flux. We further show that Glut1 is localized on the SCV membrane, establishing a putative glucose-import conduit that facilitates bacterial acquisition of cytosolic glucose. Notably, *S*. Typhimurium-induced K29-linked ubiquitination of Glut1 on vacuolar membranes potentiates the transporter activity, enhancing glucose translocation efficiency. Functional inhibition of Glut1 not only augments *S*. Typhimurium-triggered innate immune responses but also attenuates bacterial virulence in both in vitro and in vivo models. Collectively, these findings delineate a novel mechanism of metabolic hijacking, whereby *S*. Typhimurium subverts host Glut1 to reprogram glucose metabolic and immune pathways, thereby enabling its intracellular proliferation.

## Materials and methods

### Plasmids, antibodies, and reagents

For transient expression studies, the full-length open reading frames (ORFs) of GLUT1 and c-JUN were amplified from a HeLa cDNA library. *Salmonella* effector genes (SopB, SopE, SopE2) were amplified from the genomic DNA of *Salmonella* Typhimurium strain SL1344. These fragments were subsequently cloned into the pCS2-EGFP, pCS2-RFP, or pCS2-Flag vectors for mammalian cell expression. The plasmids pRK5-HA-Ub-WT and its lysine mutants were maintained in our laboratory [23, 24]. The reporter plasmids pGLUT1-Luc and pRL-TK were commercially obtained from MiaoLing Biology Company. The GLUT1 K4R mutant was generated by site-directed mutagenesis using a standard PCR-based cloning strategy. All constructs were verified by DNA sequencing prior to experimental use.

The following commercial antibodies were listed in this study. Antibodies for Glut1 (E4S7I, #73,015), NF-κB p65 (D14E12, #8242), phospho-NF-κB p65 (Ser536, 93H1, #3033), Erk1/2 (137F5, #4695), phospho-Erk1/2 (Thr202/Tyr204, D13.14.4E, #4370), JNK (#9252), phospho-JNK (Thr183/Tyr185, 81E11, #4668), p38 (D13E1, #8690), phospho-p38 (Thr180/Tyr182, D3F9, #4511), c-Jun (60A8, #9165), phospho-c-Jun (Ser73, D47G9, #3270), and HIF1α (D1S7W, #36169S) were from Cell Signaling Technology. Antibodies for Flag (AE092), HA (AE105), GFP (AE078), GAPDH (A19056), β-Actin (AC026), α-Tubulin (AC012), and H3 (A22348) were purchased from Abclonal. Antibodies for *Salmonella* (ab35156), DnaK (ab69617), and O-GlcNAc (ab2739) were from Abcam, and anti-Rab1 (11,671–1-AP) and anti-Rab5 (11,947–1-AP) antibodies were from Proteintech.

All pharmacological inhibitors were obtained from TargetMol. All compounds were dissolved in DMSO (T0341) as stock solutions and diluted to the indicated working concentrations in culture medium immediately before use. Working concentrations were used as follows: JNK inhibitor JNK-IN-8 (T2668, 5 μM), ERK1/2 inhibitor 2 (T8472, 5 μM), p38 inhibitor VX-702 (T2513, 10 μM), AP‑1 inhibitor T‑5224 (T5416, 5 μM), HIF‑1α inhibitor 2‑Methoxyestradiol (T2220, 50 μM), Glut1 inhibitor WZB117 (T7018, 50 μM), Brefeldin A (T6062, 10 μg/mL), Nocodazole (T2802, 5 μg/mL), Actinomycin D1 (T68795, 1 μg/mL) and Cycloheximide (T1225, 100 μg/mL). Other chemicals were from Sigma-Aldrich unless otherwise noted.

### Cell culture and transfection

HeLa (RRID: CVCL_0030), 293 T (RRID: CVCL_0063), RAW264.7 (RRID: CVCL_0493), MCF7 (RRID: CVCL_0031), SW480 (RRID: CVCL_0546), and A375 (RRID: CVCL_0132) cell lines were obtained from the American Type Culture Collection (ATCC). Stable HeLa cell lines expressing EGFP or EGFP-tagged proteins (VAMP8, STX7, VTI1B, SNAP25, or LC3) were generated in our previous studies and maintained in our laboratory [24]. All cells were cultured in high-glucose Dulbecco's modified Eagle medium (DMEM; HyClone) supplemented with 10% fetal bovine serum (FBS; Gibco), 2 mM L-glutamine, and 1% penicillin/streptomycin at 37 °C in a humidified 5% CO2 atmosphere. In this study, human epithelial cell lines and murine macrophages were used to explore the expression, subcellular localization, and biological function of Glut1, while 293 T cells were employed for biochemical assays due to its high transfection efficiency. All cell lines exhibited consistent growth characteristics and demonstrated the expected biological responses, including stable bacterial infection efficiency, high transfection efficiency, and inflammatory responses.

For transient transfection, cells were transfected using JetPrime transfection reagent (Polyplus) following the manufacturer's protocol. For siRNA-mediated gene silencing, 2 × 10⁶ cells were transfected with 200 pmol of siRNA using the following sequences: mGlut1 #a (5'-GGACCUCAAACUUCAUUGUTT-3'), mGlut1 #b (5'-CUGUCGGCCUCUUUGUUAATT-3'), hGLUT1 #1 (5'-CAGCCAAAGUGACAAG ACATT-3'), hGLUT1 #2 (5'-CCAAGAGUGUGCUAAAGAATT-3'), or negative control siRNA (5'-TTCTCCGAACGTGTCACGT-3'). Luciferase activity was assessed using the Dual-Luciferase Reporter Assay System (Promega) according to the manufacturer's instructions.

### Bacterial culture and infection

The pathogenic bacterial strains (*Salmonella* Typhimurium SL1344, EPEC E2348/69, *Shigella flexneri* 2457 T, *Klebsiella pneumoniae* JH1) were maintained in our lab. *Salmonella* Typhi Ty2 was kindly provided by Prof. Lu Feng and Prof. Lingyan Jiang of Nankai University. The SPI T3SS mutant derivatives of *S*. Typhimurium Δ*sipD* and Δ*ssaV* were generated by a standard homologous recombination method using the suicide plasmid pCVD442 in our previous study [25]. Bacteria were cultured in LB Broth at 37 °C with shaking. When necessary, cultures were supplemented with antibiotics with the following final concentrations: streptomycin, 100 μg/mL; ampicillin, 100 μg/mL; kanamycin, 50 μg/mL.

Bacterial infection of mammalian cells followed a previously described protocol with a few modifications [25]. Briefly, pathogenic bacteria were cultured overnight (approximately 16 h) at 37 °C on a shaker (220 rpm/min) and then subcultured in antibiotic-free LB at a dilution of 1:33 for another 3 h. Infection was performed at a multiplicity of infection (MOI) of 100 for 1 h at 37 °C. To promote and synchronize infection, 24-well plates were centrifuged at 700 g for 5 min at room temperature. The uninfected (UI) mock group was processed in parallel under identical conditions, including centrifugation at 700 g for 5 min, but without the addition of bacterial inoculum. A gentamicin-killing assay was performed to remove and kill the extracellular bacteria. Briefly, cells were washed with PBS twice, and the culture medium was replaced with fresh medium supplemented with 100 μg/mL gentamicin. After incubation for an indicated period, infected cells were collected and subjected to further analyses.

To measure *S*. Typhimurium replication within host cells, SW480 and RAW264.7 cells were infected with the indicated *S*. Typhimurium strains with an MOI of 10. A gentamicin-killing assay was performed as described above. At 1 h and 24 h post-infection, cells were lysed in cold PBS containing 1% Triton X-100, and serial dilutions were plated on the appropriate antibiotic agar plates to determine the colony-forming units. CFUs were normalized to host cell number at each time point to ensure accurate quantification of bacterial replication. The replication fold was the number of intracellular bacteria at 24 h divided by the number at 1 h.

### Actinomycin D and cycloheximide chase assays

For mRNA stability assessment, cells were treated with 1 μg/mL actinomycin D to block transcription, followed by RNA extraction at 0–10 h for RT-qPCR analysis. mRNA levels were normalized to *Gapdh* and expressed as relative quantities compared to 0 h. For protein degradation analysis, cells were treated with 100 μg/mL cycloheximide to inhibit new protein synthesis, and then harvested at the indicated time for western blotting. Protein levels were normalized to Tubulin and calculated as percentages relative to 0 h. All experiments were performed with triplicate biological replicates, and the degradation rates were determined by plotting relative levels at each time point.

### Magnetic nanoparticle labeling of *S*. Typhimurium and SCV isolation

*S*. Typhimurium labeling and SCV isolation were performed as previously described with modifications [26]. Briefly, carboxyl-coated paramagnetic Fe₃O₄ nanoparticles (30 nm diameter; Zhongkekeyou Beads, China) were prepared by sonicating an aliquot of the 3 mg/mL stock solution to disperse aggregates, followed by 1:10 dilution in sterile deionized water. Mid-log phase *S*. Typhimurium cultures (OD₆₀₀ ≈ 0.8) were pelleted (16,000 g, 5 min), resuspended in PBS, and incubated with nanoparticles at a 5:1 (v/v) ratio (37 °C, 20 min, 200 rpm shaking). Labeled bacteria were purified through a 0.2 μm syringe filter, washed twice with PBS, and recovered by back-flushing with 1 mL PBS. Bacterial viability and concentration were determined by serial dilution plating on LB agar.

For SCV isolation, mammalian cells were infected with labeled *S*. Typhimurium at MOI = 50 for 10 h. Cells were washed with PBS and lysed in 0.1% Triton X-100. As a background control, uninfected cells were processed in parallel by directly adding the same carboxyl-coated paramagnetic Fe₃O₄ nanoparticles (without bacteria) to the cells. Lysates were subjected to magnetic separation (Magnetic Separation Rack, NEB) for 5 min at 4 °C. The magnetic fraction was washed twice with PBS containing 2% sucrose, and the final pellet was collected. Isolated SCVs were either processed immediately or stored at −80 °C for downstream applications, including glucose uptake assays, immunoprecipitation, or immunoblotting.

### Glucose uptake and glycolytic rate assays

Glucose uptake was quantified using the Glucose Uptake-Glo™ Assay (Promega) following the manufacturer’s protocol with some modifications. Briefly, SCV-enriched particles or intact cells were washed with PBS and incubated with 1 mM 2-deoxyglucose (2-DG) in PBS for 10 min. The reaction was terminated using acidic Stop Buffer, followed by neutralization. Lysates were then mixed with 2DG6P detection reagent and incubated for 1 h at room temperature. Luminescence was quantified using a microplate reader to determine 2DG6P levels.

The extracellular acidification rate (ECAR) was measured using the Seahorse XF Glycolytic Rate Assay Kit (Agilent Technologies) following the manufacturer's protocol. Briefly, RAW264.7 macrophages were mock-infected or infected with *S*. Typhimurium for 10 h, then seeded onto Seahorse XF96 cell culture microplates at a density of 2 × 10^4^ cells per well. Prior to the assay, cells were washed and maintained in Seahorse XF DMEM medium (pH 7.4) supplemented with 2 mM glutamine, 10 mM glucose, and 1 mM pyruvate. The assay was performed using a Seahorse XFe96 Analyzer with the following measurement cycle: 3 min mixing, 2 min waiting, and 3 min recording. After baseline ECAR measurements, sequential injections of 10 μM oligomycin and 50 mM 2-DG were performed to assess glycolytic flux. Data were normalized to total cell number and analyzed using the Wave Desktop Software (Agilent Technologies).

### Biotinylated DNA synthesis and DNA pull-down assay

The Glut1 promoter region (−1800 to + 100 bp relative to TSS) was PCR-amplified from RAW264.7 genomic DNA using a biotin-11-UTP: TTP mixture (1:10 ratio) to generate the biotinylated DNA. Successful biotin labeling was confirmed by electrophoretic detection, with labeled fragments exhibiting slight size retardation compared to unmodified DNA. For the DNA pull-down assay, 2 μg of biotinylated probes were incubated with 500 μg of RAW264.7 nuclear extracts in binding buffer supplemented with 1 μg poly(dI-dC) at 4 °C for 2 h with rotation. DNA–protein complexes were captured using streptavidin magnetic beads for 1 h at 4 °C, followed by five washes and immunoblotting analyses.

### Immunoprecipitation

The transfected intact cells or enriched SCVs were washed once with PBS and lysed in ice-cold Buffer A (25 mM Tris–HCl, pH 7.5, 150 mM NaCl, 10% glycerol, 1% Triton X-100) supplemented with protease inhibitors. The lysates were briefly sonicated (20W power, 3-s pulses with 7-s intervals for 5 cycles) to ensure complete disruption and reduce viscosity. After centrifugation to remove debris, the supernatants were pre-cleared and subjected to anti-Flag immunoprecipitation following standard procedures. The immunoprecipitates were washed four times with ice-cold wash buffer B (25 mM Tris–HCl, pH 7.5, 150 mM NaCl, 0.5% Triton X-100), then eluted and for subsequent immunoblotting or MS analyses.

### Immunofluorescence microscopy and image analysis

For immunofluorescence staining, transfected or infected cells grown on coverslips were fixed in 4% paraformaldehyde for 10 min, permeabilized with 0.2% Triton X-100 for 15 min, and blocked with 2% BSA for 30 min. After incubation with primary antibodies, samples were labeled with species-matched Alexa Fluor-conjugated secondary antibodies. The nuclei were counterstained with DAPI for 2 min. Confocal images were acquired using a confocal microscope (FV3000RS, Olympus), with identical acquisition settings maintained across compared samples. Quantitative colocalization analysis was performed using ImageJ, and the colocalization index was calculated from at least 30 cells derived from three independent experiments. All images were processed uniformly with background subtraction, and threshold adjustments were applied equally to the control and experimental groups.

### Dual RNA-seq analysis

For dual RNA-seq analysis, RAW264.7 macrophages were infected with *S* Typhimurium SL1344 at an MOI of 100. At 10 h post-infection, cells were lysed in TRIzol reagent (Invitrogen), and total RNA was extracted following the manufacturer's protocol. RNA quality was assessed using an Agilent 2100 Bioanalyzer, and all samples had RNA integrity numbers (RIN) greater than 8.5.

Library preparation and sequencing were performed by Novogene (Beijing, China). Briefly, ribosomal RNA was depleted using the Ribo-Zero Gold Kit (Illumina), and stranded RNA-seq libraries were constructed using the NEBNext Ultra II Directional RNA Library Prep Kit. Sequencing was conducted on an Illumina NovaSeq 6000 platform with 150 bp paired end reads, generating approximately 40 million read pairs per sample.

For bioinformatic analysis, raw reads were first processed with Fastp (v0.23.2) to remove adapters and low-quality sequences. The cleaned reads were then simultaneously aligned to the murine reference genome (GRCm39) and the *S.* Typhimurium SL1344 reference genome (NCBI Assembly: GCF_000210855.2). Gene expression levels were quantified as FPKM (Fragments Per Kilobase of transcript per Million mapped reads) values using feature Counts (v2.0.3).

Differentially expressed genes (DEGs) were identified using DESeq2 (v1.38.3) with a significance threshold of -log_10_(*p*-value) > 1.3 (equivalent to *p* < 0.05). Functional enrichment analysis of DEGs was performed through Gene Ontology (GO) and Kyoto Encyclopedia of Genes and Genomes (KEGG) pathway analyses using the DAVID online tool (https://david.ncifcrf.gov/). Significantly enriched terms were defined as those with Benjamini–Hochberg adjusted *p*-value < 0.05.

### Metabolomics analysis

Cells were washed twice with ice-cold phosphate-buffered saline (PBS) and rapidly quenched in liquid nitrogen to preserve metabolic states. Cell samples were collected by scraping and stored at −80 °C until analysis. Metabolites were extracted using a methanol:acetonitrile:water (2:2:1, v/v/v) system. Untargeted metabolomic profiling was performed by PANOMIX Biomedical Tech Co., Ltd (Suzhou, China) using an ultra-high-performance liquid chromatography system (Vanquish, Thermo Fisher Scientific) coupled to a Q-Exactive HF-X mass spectrometer (Thermo Fisher Scientific). Chromatographic separation was achieved on a HILIC column (Waters ACQUITY UPLC BEH Amide, 2.1 × 100 mm, 1.7 μm) maintained at 40 °C, with a flow rate of 0.3 mL/min and an injection volume of 5 μL. The mass spectrometer operated in both positive and negative ionization modes with a mass range of 70–1050 m/z and resolution of 120,000.

Raw data were processed using Xcalibur 4.0 software (Thermo Fisher Scientific) for peak alignment, retention time correction, and feature extraction. Multivariate statistical analysis, including principal component analysis (PCA) and orthogonal partial least squares-discriminant analysis (OPLS-DA), was performed in MetaboAnalyst 5.0. Metabolite identification was achieved by matching accurate mass (mass accuracy < 5 ppm) and MS/MS fragmentation patterns against standard references in the Human Metabolome Database (HMDB) and METLIN databases.

Significantly altered metabolites were defined as those with *p* < 0.05 (Student's t-test). Pathway enrichment analysis was conducted by mapping these metabolites to KEGG pathways using MetaboAnalyst 5.0. All experiments included three independent biological replicates per condition, with quality control samples (pooled from all samples) analyzed throughout the sequence to monitor instrument stability.

### Mass spectrometry analyses

Mass spectrometry analyses were performed following established protocols with minor modifications [24, 25, 27]. All experiments were conducted with at least two independent biological replicates, each consisting of three pooled technical replicates. Two complementary MS approaches were employed to characterize Glut1 ubiquitination and its protein interactome. For the ubiquitinated peptide identification. Flag-tagged Glut1 was immunopurified from SCV or the uninfected cells, followed by in-solution digestion with sequencing-grade trypsin (Promega) at 37 °C for 16 h. The resulting peptides were separated using an EASY-nLC 1200 UHPLC system (Thermo Fisher Scientific) with a 60-min nonlinear gradient: 0–8% mobile phase B over 3 min, 8–28% B over 42 min, 28–38% B over 5 min, and 38–100% B over 10 min (mobile phase A: 0.1% formic acid in water; B: 80% acetonitrile/0.1% formic acid). Eluted peptides were analyzed on a Q Exactive Plus mass spectrometer (Thermo Fisher Scientific) operating in data-dependent acquisition mode. Full MS scans (m/z 350–1600) were acquired at 70,000 resolution, followed by HCD fragmentation of the top 20 most intense precursors at 17,500 resolution. For interactome identification, immunoprecipitated complexes were resolved by SDS-PAGE and visualized by silver staining. Entire gel lanes were excised, destained, reduced with DTT, alkylated with iodoacetamide, and digested with trypsin overnight at 37 °C. Extracted peptides were analyzed by LC–MS/MS using the same instrumentation and gradient conditions described above.

All raw spectra were processed using Proteome Discoverer software (v2.2, Thermo Fisher Scientific). Database searches were performed against the human proteome (UniProtKB) supplemented with Glut1 sequence and common contaminants. Search parameters included: trypsin specificity with up to 4 missed cleavages; precursor mass tolerance of 10 ppm, fragment mass tolerance of 0.02 Da, fixed modification of cysteine carbamidomethylation, variable modifications including methionine oxidation, lysine ubiquitination (Gly-Gly remnant, + 114.04 Da), and protein N-terminal acetylation. False discovery rates were determined using Percolator, with thresholds set at 1% for both peptide-spectrum matches and protein identifications.

### Mouse infection and bacterial virulence assessment

Five- to six-week-old wild-type C57BL/6 mice were obtained from the Experimental Animal Center of Hubei University of Medicine (Hubei, China) and housed under specific pathogen-free (SPF) conditions. All experimental protocols were approved by the Institutional Animal Care and Use Committee of Hubei University of Medicine (Protocol #03125020 K) and performed in accordance with national standards for animal welfare.

Mice (*n* = 10 per group) were pretreated for 7 days with daily intraperitoneal injections of WZB117 (10 mg/kg, formulated in 10% DMSO, 40% PEG300, 5% Tween 80, and 45% saline) or vehicle control, followed by intraperitoneal infection with 5 × 10^4^ CFU of S. Typhimurium. Survival was monitored for 82 h post-infection; surviving animals were then euthanized for tissue harvest. Serum was collected for cytokine quantification by ELISA. Liver and spleen samples were processed for bacterial load determination (via serial dilution and plating), histopathological examination (H&E staining), and molecular profiling (immunoblotting and RT‑qPCR).

### Immunohistochemical analysis

All procedures, including tissue sectioning and immunohistochemical staining, were performed by Servicebio Co., Ltd. (Wuhan, China). Briefly, liver tissues were fixed in 4% paraformaldehyde, processed through graded ethanol and xylene, and embedded in paraffin. Sections were cut at 3 μm thickness using a rotary microtome. Antigen retrieval was performed by heating slides in sodium citrate buffer (10 mM, pH 6.0) at 95 °C for 10 min. After blocking endogenous peroxidase activity with 3% H₂O₂, sections were incubated with anti-GLUT1 antibody (1:100 dilution) overnight at 4 °C, followed by appropriate HRP-conjugated secondary antibody.

Immunoreactivity was visualized using 3,3'-diaminobenzidine (DAB) as chromogen with hematoxylin counterstaining. The immunoreactive score (IRS) was determined using a semi-quantitative method [28] that evaluates both staining intensity and percentage of positive cells. The final IRS was calculated as the product of intensity and percentage scores, ranging from 0 to 12.

### Statistical analysis

Statistical data are shown as mean ± standard deviation (SD) from at least three independent replicates. The student’s unpaired *t*-test method was used to compare two experimental groups, and the one-way analysis of variance (ANOVA) method was used to compare multiple groups. A difference is considered significant as follows: *p < 0.05, **p < 0.01.

## Results

### *S.* Typhimurium infection increases host Glut1 expression and promotes glycolytic metabolism in murine macrophages

**Fig. 1:**
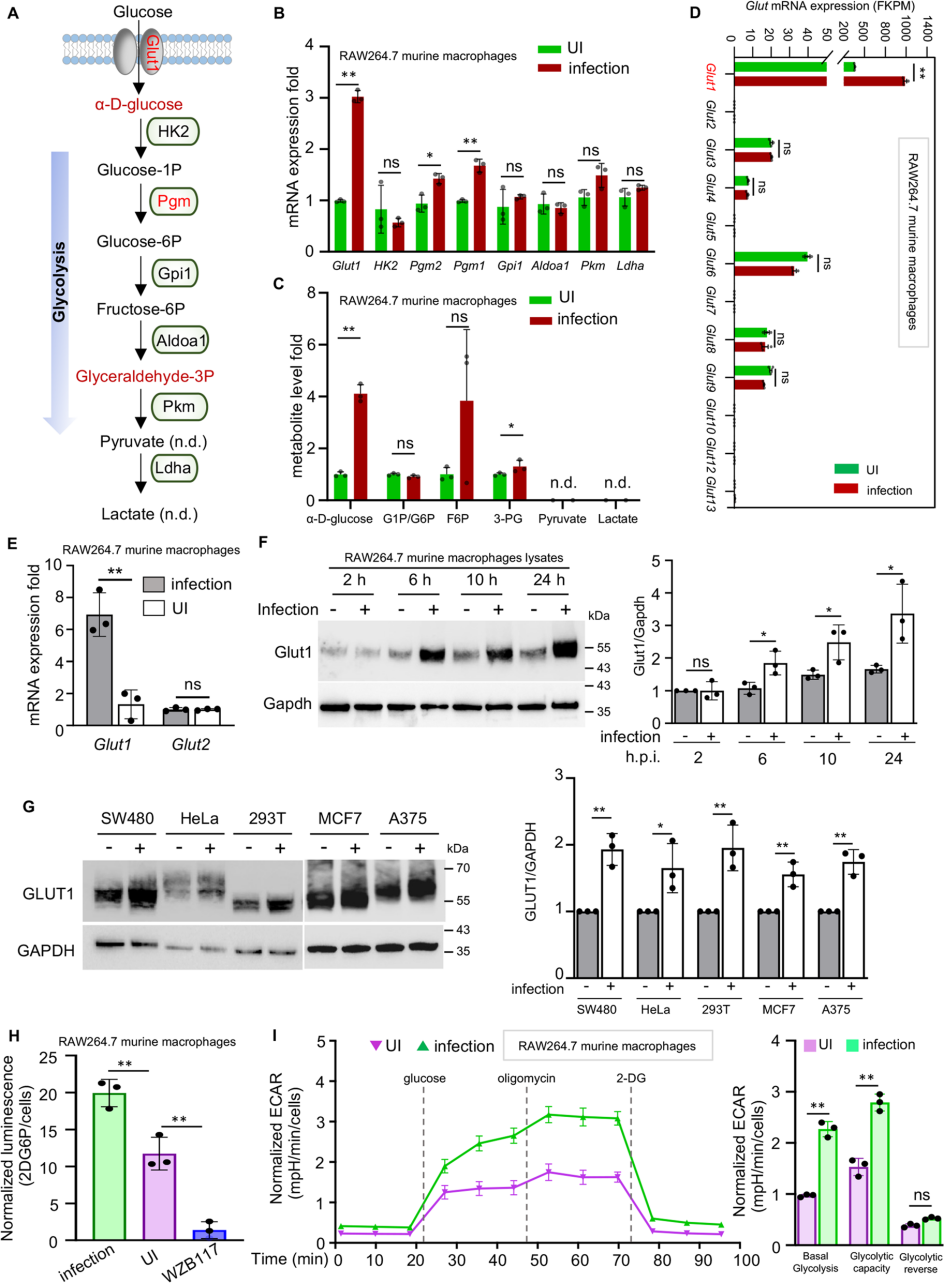
S. Typhimurium infection up-regulates Glut1 and enhances glycolysis in murine macrophages. **A**-**C** Transcriptomic and metabolomic profiling of the glycolytic pathway in RAW264.7 macrophages at 10 h post *S*. Typhimurium infection. **A** Schematic overview of pathway alterations. Components with significant changes (*p* < 0.05) are highlighted in red; non-detected metabolites are labeled “n.d.”. The overall omics analyses are provided in Supplementary Fig. 1. **B** Transcript levels of glycolytic genes from RNA-seq in infected versus uninfected (UI) RAW264.7 cells. **C** Relative abundance of glycolytic intermediates from metabolomics in infected versus uninfected cells. **D** Expression of glucose transporter (Glut) family members from RNA-seq in infected versus uninfected RAW264.7 cells. **E** qPCR validation of *Glut1* and *Glut2* mRNA expression in infected versus uninfected RAW264.7 cells at 10 h post-infection. **F** Time-course immunoblot analysis of Glut1 protein expression in RAW264.7 cells from 2–24 h post-infection. Each infected group was compared with its time‑matched uninfected control. **G** Western blot analysis of GLUT1 protein levels across multiple cell lines at 10 h post-infection. For F and G, a representative blot from three independent experiments is shown. Densitometric quantifications of Glut1, normalized to the respective loading control, are shown to the right of each blot. **H** Glucose uptake in infected versus uninfected RAW264.7 cells at 10 h post-infection, assessed by measuring intracellular 2-deoxy-D-glucose-6-phosphate (2-DG6P) normalized to total cell count. WZB117 was used as a positive control. **I** Extracellular acidification rate (ECAR) in infected versus uninfected RAW264.7 cells at 10 h post-infection. Shown are real-time ECAR tracing (left) and quantitative analysis of ECAR at distinct glycolytic stages (right). The data (**B**, **C**, **E**–**G**) are expressed as fold change relative to the uninfected control (set as 1). All bar graphs represent mean ± SD from three independent experiments. Statistical significance was determined by Student’s *t*-test. *p < 0.05, **p < 0.01. ns, not significant

To investigate how *S*. Typhimurium modulates host metabolism, we performed transcriptomic and metabolomic profiling of infected RAW264.7 macrophages. Infection induced widespread changes in host gene expression and metabolite levels. In terms of transcriptomics, the most significantly enriched pathways were immune response and metabolic regulation, among others (Supplementary Fig. 1A-B). In metabolomics, KEGG analysis revealed that the affected metabolic pathways included immune response, central carbon metabolism, and amino acid metabolism, among others (Supplementary Fig. 1C-D). Among the metabolic pathways altered, some glycolysis-related genes and metabolites showed consistent changes. Specifically, the expression of *Glut1* and *Pgm1/2* was significantly upregulated, accompanied by elevated intracellular levels of glucose and 3-phosphoglycerate (3-PG). Among these alterations, the upregulation of Glut1 and the concomitant accumulation of glucose represented the most prominent changes (Figs. 1A-C). This phenomenon was consistently observed in primary macrophages, where elevated *Glut1* expression was detected during *Salmonella* infection [11, 13, 20].

Among all glucose transporters (*Glut1*-*Glut13*), *Glut1* showed the most dramatic transcriptional induction upon infection, while other members remained largely unchanged (Fig. 1D). This specific upregulation was confirmed by qPCR (Fig. 1E). Western blot analysis further revealed a time-dependent upregulation of Glut1 protein in response to *S*. Typhimurium infection compared to its time‑matched uninfected controls (Fig. 1F). This upregulation was observed not only in RAW264.7 macrophages but also consistently across five additional cell lines, suggesting that *S*. Typhimurium may employ a conserved mechanism in Glut1 regulation (Fig. 1G). The molecular weight heterogeneity of Glut1 across cell lines may arise from variations in *N*-linked glycosylation [29].

Given the central role of Glut1 in glucose uptake, we assessed its functional contribution in RAW264.7 macrophages. Using a 2DG uptake assay, we found that infection significantly increased 2DG6P accumulation (Fig. 1H). Seahorse analysis further showed elevated ECAR in infected cells, indicating enhanced glycolytic capacity (Fig. 1I). Together, these results demonstrate that *S*. Typhimurium infection induces Glut1 upregulation and promotes host glycolysis in murine RAW264.7 macrophages, identifying Glut1 as a key target in *S*. Typhimurium-induced metabolic reprogramming.

### *S.* Typhimurium infection induces Glut1 expression through the Erk/Jnk-c-Jun signaling axis via SPI-1 effectors

Metabolic reprogramming is one of the hallmarks of host–pathogen interactions. We next examined the mechanisms underlying Glut1 upregulation during infection. Using actinomycin D (Act D) and cycloheximide (CHX) chase assays, we found that *S*. Typhimurium infection of RAW264.7 macrophages did not significantly alter Glut1 mRNA stability or protein degradation, indicating that Glut1 upregulation primarily results from enhanced de novo synthesis rather than impaired degradation (Supplementary Fig. 2).

**Fig. 2:**
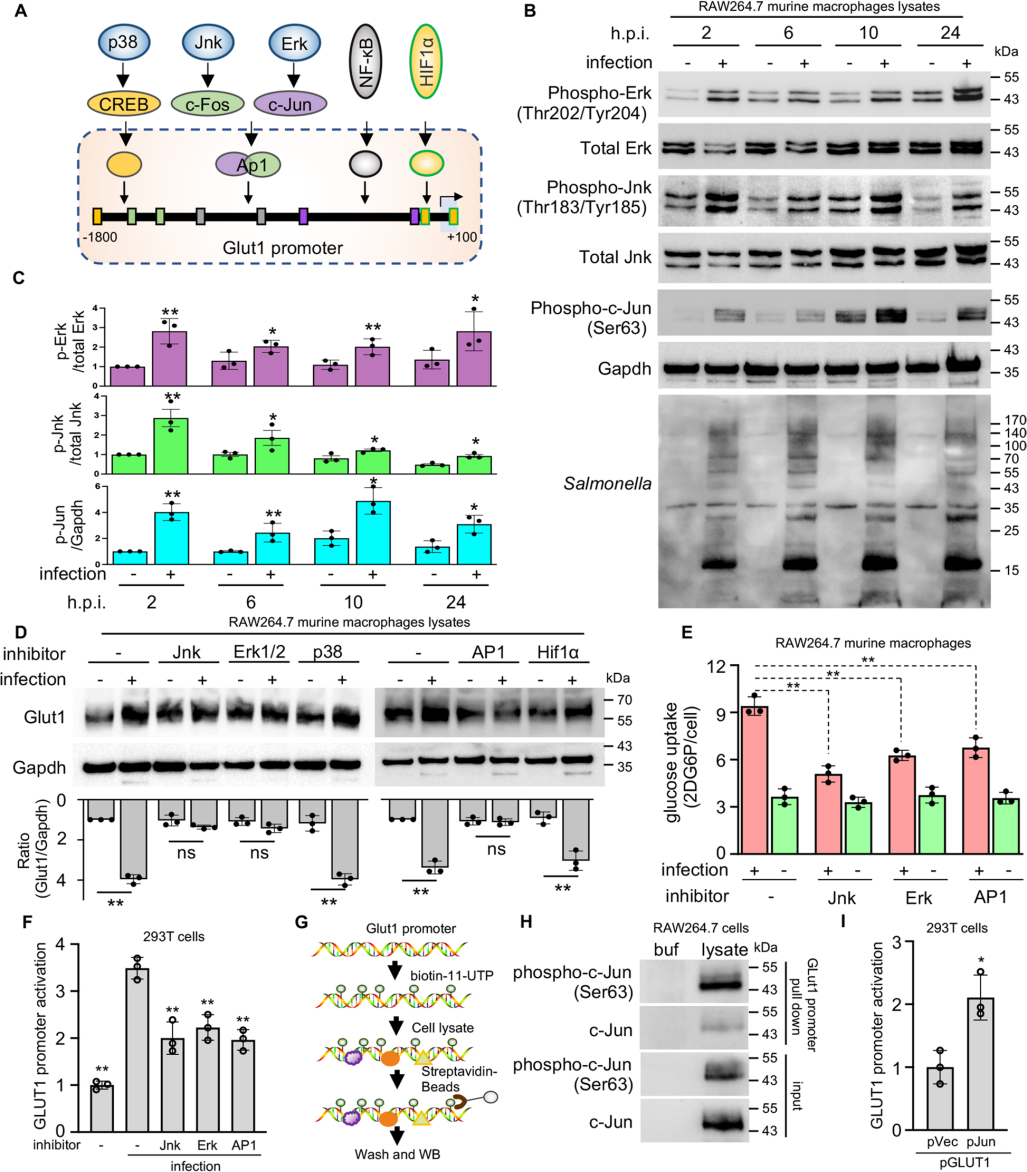
***S.*** Typhimurium infection induced Glut1 expression via Erk/Jnk-Ap1 axis. **A** In silico analysis of the Glut1 promoter. Schematic representation of the predicted transcription factor binding sites within the murine *Glut1* promoter region (−1800 to + 100 bp relative to the transcription start site, TSS). The analysis was performed using the JASPAR database. Potential regulatory sites are color-coded according to their corresponding transcription factors. **B**-**C** The effects of *S*. Typhimurium infection on MAPK pathway activation. **B** Time-course immunoblot analysis of MAPK signaling components (Erk1/2, Jnk, and c-Jun) in RAW264.7 cells post-infection (2–24 h.p.i.). Gapdh serves as the loading control, and a *Salmonella*-specific antibody was used as an infection control. Representative blots from three independent experiments are shown. **C** Densitometric quantification of phosphorylated MAPK levels, normalized to their respective total protein levels. For phospho‑c‑Jun, normalization was performed against GAPDH because total c‑Jun is also upregulated during infection (Supplementary Fig. 3 A). Data are expressed as fold change relative to the value in uninfected cells at the 2-h time point (set as 1). Each infected group was compared with its time‑matched uninfected control. **D**-**F** Pharmacological inhibition of MAPK signaling impairs *S*. Typhimurium-induced Glut1 upregulation and functional enhancement. **D** RAW264.7 cells were pretreated with the indicated MAPK pathway inhibitors (for specific inhibitors and concentrations used, see Materials and Methods) for 2 h and then either infected with *S*. Typhimurium or mock-infected for an additional 10 h (inhibitors were maintained throughout the infection). Glut1 protein levels were assessed by immunoblot. Representative blots from three independent experiments are shown. Densitometric quantification of Glut1, normalized to Gapdh, is presented below the blot. Data are expressed as fold change relative to the untreated, uninfected control (set as 1). **E** Glucose uptake activity was measured in RAW264.7 cells under the same pretreatment and infection conditions as described in (**D**). **F** 293 T cells were transfected with a GLUT1 promoter-driven luciferase reporter plasmid for 18 h, pretreated with MAPK inhibitors for 2 h, and then infected with *S*. Typhimurium for another 10 h (inhibitors were present throughout). Glut1 luciferase activation is shown as a fold change normalized to the unstimulated and uninfected control cells. **G**-**I** The transcription factor c-Jun directly binds to and activates the Glut1 promoter. **G** Schematic illustration of the DNA pull-down assay. A biotin-labeled DNA fragment covering the Glut1 promoter region was incubated with nuclear extracts prepared from RAW264.7 cells. Lysis buffer alone served as a negative control. Proteins bound to the biotinylated promoter were captured using streptavidin-conjugated magnetic beads. **H** Immunoblot analysis of the DNA pull-down samples and the corresponding nuclear extracts (input) using antibodies against c-Jun and phospho-c-Jun (Ser63). Representative blots from three independent experiments are shown. **I** 293 T cells were co-transfected with the pGLUT1-Luc reporter plasmid together with either an empty vector control or a c-JUN expression plasmid. Luciferase activity was measured at 18h post-transfection. All bar graphs represent mean ± SD from three independent experiments. Statistical significance was determined by Student’s *t*-test. *p < 0.05, **p < 0.01. ns, not significant

Promoter analysis of the *Glut1* gene identified multiple putative transcription factor binding sites, including those for MAPK pathway components (Erk1/2, Jnk, p38) downstream effectors (c-Jun, c-Fos, CREB), as well as NF-κB p65 and HIF1α (Fig. 2A). Western blot analysis showed that *S*. Typhimurium infection of RAW264.7 macrophages specifically enhanced phosphorylation of Erk, Jnk, and their downstream target c-Jun, whereas the activation states of p38 MAPK, NF-κB p65, and HIF1α remained unaltered (Supplementary Fig. 3 and Figs. 2B-C). Pharmacological inhibition experiments revealed that treatment with inhibitors targeting Erk1/2, Jnk, or AP-1 (a heterodimeric complex of c‑Jun and c‑Fos) significantly suppressed *S*. Typhimurium-induced Glut1 expression, glucose uptake, and *Glut1* promoter activation (Figs. 2D-F). DNA pull-down assays further confirmed that both phosphorylated and non-phosphorylated c-Jun directly bind to and activate the Glut1 promoter (Figs. 2G-I), establishing a direct regulatory mechanism.

**Fig. 3:**
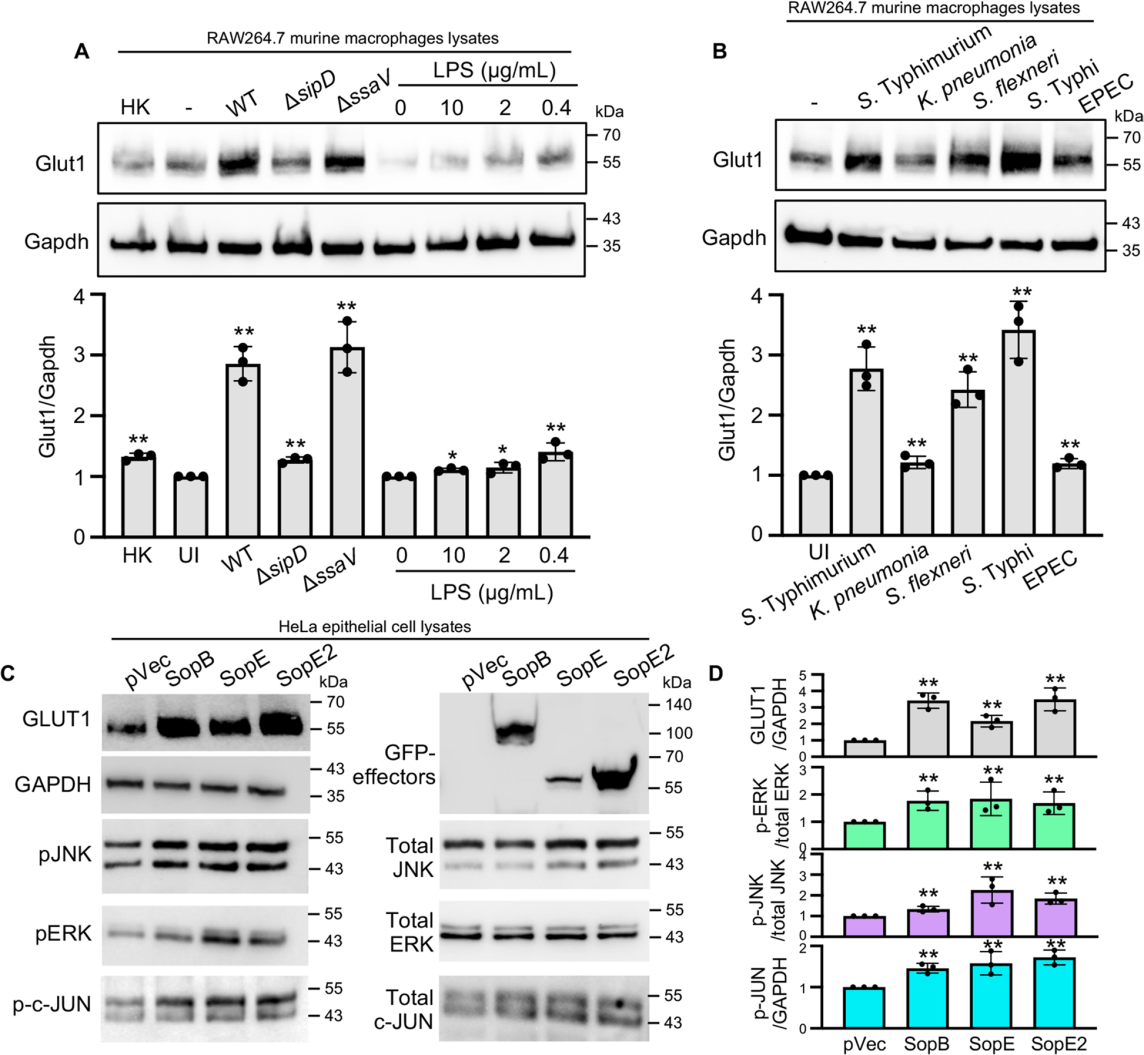
S. Typhimurium upregulates Glut1 expression in an SPI-1 T3SS-dependent manner. **A** Effects of *S*. Typhimurium mutants and LPS on Glut1 expression. RAW264.7 cells were infected with the indicated *S*. Typhimurium mutants (left) or treated with the specified concentrations of LPS for 10 h (right). HK, heat-killed. WT, wild-type. Δ*sipD*, SPI-1 T3SS-deficient mutant. Δ*ssaV*, SPI-2 T3SS-deficient mutant. **B** Glut1 induction by different bacterial pathogens. RAW264.7 cells were mock-infected or infected with the indicated bacteria at an MOI of 100 for 10 h. For (**A**) and (**B**), Glut1 protein levels were assessed by immunoblotting. Densitometric quantification of Glut1, normalized to Gapdh, is presented below the blots. Data are expressed as fold change relative to the respective untreated or uninfected control (set as 1). C**-**D Ectopic expression of SPI-1 T3SS effector proteins (SopB, SopE, SopE2) enhances GLUT1 levels. **C** HeLa cells were transfected with an empty vector (pVec) or plasmids expressing GFP-tagged effectors for 18 h, followed by immunoblotting with antibodies against the indicated proteins. **D** Densitometric quantification of GLUT1 and phosphorylated MAPK levels from (**C**), normalized to GAPDH and total MAPK, respectively. Data are expressed as fold change relative to the empty vector control (set as 1). For all panels, representative immunoblots from three independent experiments are shown. Bar graph data are presented as mean ± SD (*n* = 3). Statistical significance was determined by Student’s *t*-test. *p < 0.05, **p < 0.01

To identify the specific bacterial components responsible for *S*. Typhimurium-induced Glut1 upregulation, we conducted a series of experiments. LPS treatment only slightly induced Glut1 expression, with a much weaker effect than *S.* Typhimurium infection. Notably, LPS induced Glut1 in a concentration‑dependent manner, with peak expression at 0.4 μg/mL and reduced expression at higher concentrations (2 and 10 μg/mL). This pattern is consistent with the distinct, concentration-specific signaling outputs of the MyD88 and TRIF pathways downstream of TLR4 activation [30]. Using T3SS-deficient mutants, we found that both SPI-1 T3SS-deficient (Δ*sipD*) and heat-killed (HK) *S*. Typhimurium very slightly induced Glut1 expression, suggesting this upregulation may represent a pathogen-specific response rather than a general host reaction to bacterial infection (Fig. 3A). Consistent with this, among the pathogens tested, invasive intracellular bacteria (*Salmonella* and *Shigella*) induced Glut1 expression to a significantly greater extent, while extracellular pathogens (EPEC and *Klebsiella*) showed only minimal effects, likely mediated by their LPS (Fig. 3B). These results demonstrate that Glut1 upregulation requires SPI-1 T3SS-dependent bacterial invasion in RAW264.7 macrophages.

Given that *Salmonella* SPI-1 effectors (SopB/SopE/SopE2) promote bacterial invasion by hijacking host Rho GTPases for actin remodeling while simultaneously activating MAPK pathways [31], we hypothesized that these effectors might mediate Glut1 upregulation. Indeed, ectopic expression of individual effectors in HeLa cells significantly enhanced Glut1 expression while synchronously activating the Jnk/Erk-c-Jun axis (Fig. 3C-D). In line with these immunoblotting data, immunofluorescence staining shows that cells expressing GFP-tagged SopB, SopE, or SopE2 exhibited increased GLUT1 mean fluorescence intensity compared with neighboring non-transfected cells or cells expressing GFP alone (Supplementary Fig. 4), suggesting a potential contribution of these SPI-1 effectors in GLUT1 induction.

### Glut1 associates with intracellular SCVs and facilitates glucose acquisition by intravacuolar *S.* Typhimurium

**Fig. 4:**
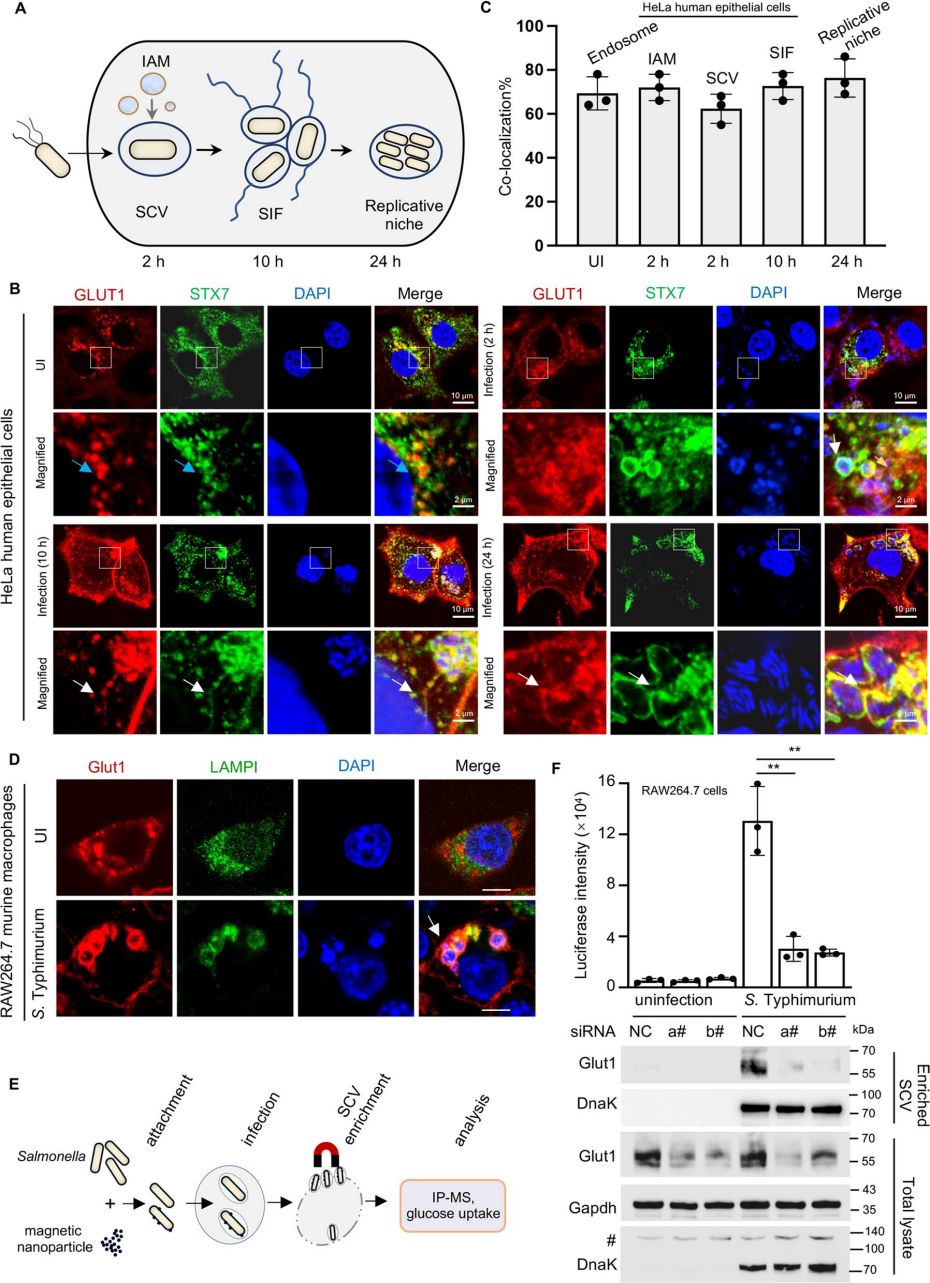
Glut1 co-localizes with SCVs and is important for glucose acquisition by intravacuolar ***S***. Typhimurium. **A**–**C** Co-localization of GLUT1 with *S*. Typhimurium intracellular vacuolar replicative structure in HeLa epithelial cells. **A** Schematic diagram of *Salmonella*-containing vacuolar maturation in host epithelial cells. **B**-**C** HeLa cells stably expressing EGFP-STX7 were infected with the *S*. Typhimurium for the indicated time. **B** Representative images showing the distribution of STX7 (green), endogenous GLUT1 (red), and the nucleus (blue). Scale bar, 10 µm. White boxes indicate magnified regions. Scale bar, 2 µm. Arrows indicated the co-localization of GLUT1 with endosome (blue), SCV/SIF (white), and IAM (yellow). **C** The co-localization rate of STX7 and GLUT1 for each sample. At least 100 cells were counted for samples from experiments done in triplicate. **D** Co-localization of Glut1 with *S*. Typhimurium in macrophages. RAW264.7 cells were infected with *S*. Typhimurium for 16 h. Representative images show endogenous Glut1 (red), LAMPI (green) and nuclei (blue). Scale bar, 10 µm. **E** Schematic illustration of SCV enrichment using magnetic nanobeads. Experimental details are provided in the Materials and Methods section. **F** Effects of *Glut1* knockdown on the glucose uptake of SCV. RAW264.7 cells were transfected with two distinct Glut1-targeting siRNAs or negative control (NC) siRNA for 48 h, followed by *S*. Typhimurium infection for 10 h. Uninfected RAW264.7 cells processed in parallel with blank magnetic beads served as the background control. SCVs were isolated from the infection group and subjected to glucose uptake measurement. Equivalent DnaK levels confirmed comparable bacterial abundance among groups, and Glut1 knockdown efficiency was verified by immunoblotting. Data represent mean ± SD from three independent experiments (**p < 0.01)

Glut1 undergoes continuous endocytosis from the plasma membrane and is subsequently recycled back to the cell surface through endosomal trafficking, constituting a dynamic cycle that maintains glucose uptake homeostasis [32]. Given that *Salmonella* establishes its intravacuolar niche and extensively remodels host membrane trafficking pathways (Fig. 4A), we asked whether infection influences the subcellular localization of Glut1. We employed the SNARE protein Syntaxin 7 (STX7) as a marker for SCV membranes, which has recently been identified as a component of SCV membranes [33, 34]. In uninfected HeLa cells, Glut1 predominantly colocalized with STX7-positive endosomes. Following infection, STX7 was aberrantly recruited to the expanding SCV, demonstrating bacterial capacity to hijack endosomal compartments. Notably, Glut1 followed a nearly identical trajectory, becoming markedly enriched on STX7-decorated IAM and SCV membranes at the time points examined and remaining detectable with all subsequent SIFs and replicative vacuolar structures in fixed samples (Figs. 4B-C). This localization pattern was consistently observed in RAW264.7 murine macrophages at late infection stages (16 h.p.i.), where Glut1 colocalized with endogenous LAMP1 and prominently encircled the intravacuolar bacteria (Fig. 4D).

We next investigated the functional roles of Glut1 for the intravacuolar *S*. Typhimurium. Given that SCV/SIF-localized proteins may influence vacuolar membrane integrity [33], we asked whether Glut1, which also localizes to these compartments (Fig. 4B), plays a similar structural role. However, Glut1 knockdown did not affect the integrity of these membrane structures in *S*. Typhimurium-infected HeLa cells, as evidenced by unchanged frequencies of STX7-coated bacteria (Supplementary Figs. 5A-B**)**. Additionally, contrary to its reported possible role in regulating autophagy [35], *Glut1* knockdown did not affect the bacterial xenophagy, as shown by the unchanged proportion of LC3-coated *S*. Typhimurium Δ*sopF* mutant in HeLa cells (Supplementary Figs. 5C-D**)**.

Since Glut1 mediates bidirectional glucose transport along concentration gradients [21, 36], we hypothesized that its SCV association supports intravacuolar bacterial glucose acquisition. To directly test this, we employed a nanoparticle-based magnetic isolation strategy to purify SCVs from infected cells (Fig. 4E). As a background control, uninfected cells were processed in parallel using the same magnetic separation procedure with empty beads. The isolated vesicles contain no bacteria and therefore lack bacterial glucose metabolic enzymes, producing only a minimal background signal. Compared with the negative control (NC), Glut1 knockdown in RAW264.7 murine macrophages led to a significant reduction in glucose uptake by the isolated SCV fraction, under conditions of comparable bacterial loads (as indicated by comparable DnaK levels) (Fig. 4F). These results indicate that Glut1 enrichment on the SCV membrane functions primarily as a critical glucose acquisition system for intracellular *S*. Typhimurium, rather than playing a structural role in vacuole biogenesis or participating in antibacterial autophagy.

### Glut1 association with the SCV partially depends on host vesicular trafficking machinery

**Fig. 5:**
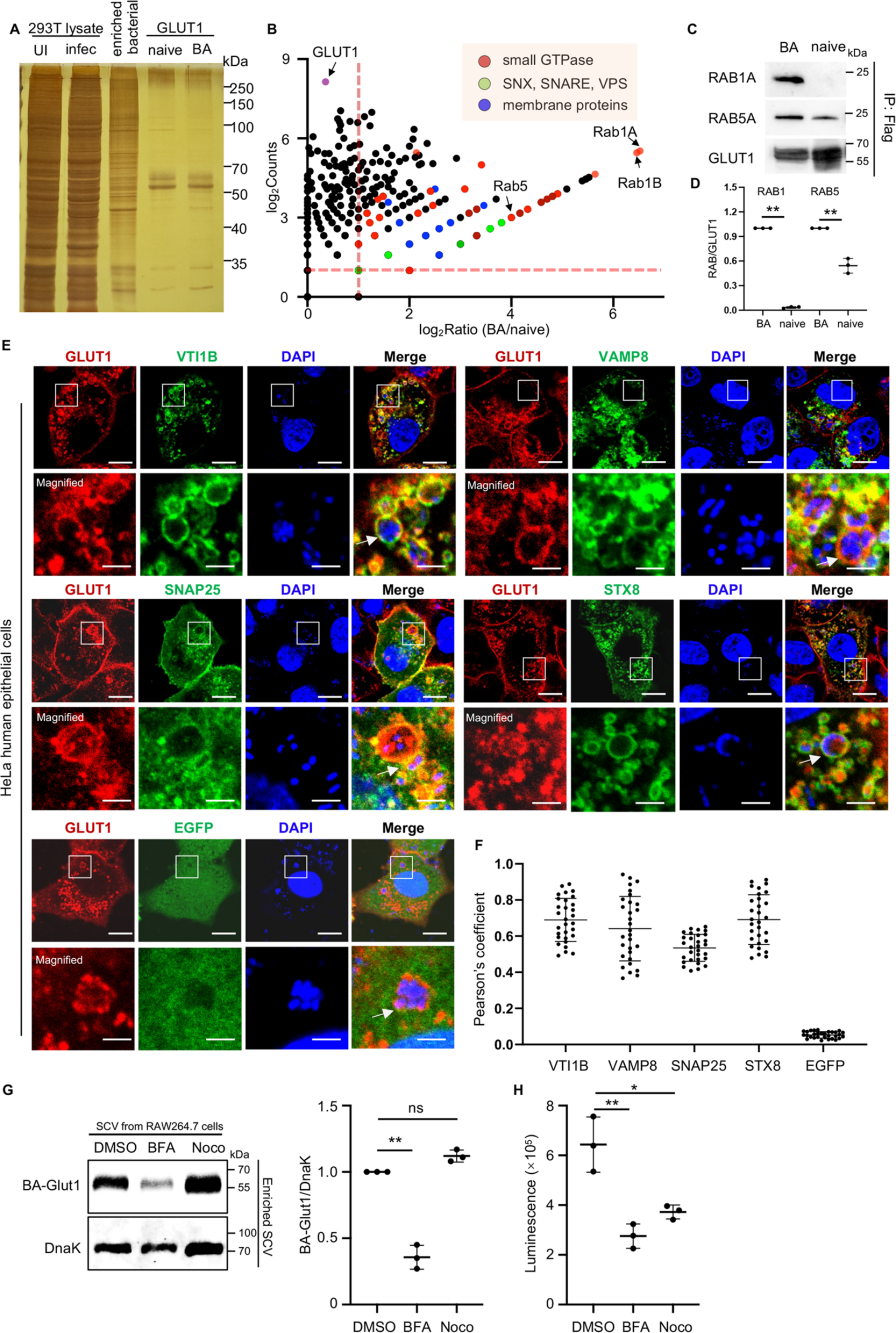
Glut1 association with SCVs depends on interaction with host vesicular trafficking proteins in a BFA-sensitive manner. **A**-**D** Identification of specific Glut1-associated proteins on SCVs. **A** Detection of enriched proteins by silver staining. Flag-Glut1-expressing 293 T cells were either infected with *S*. Typhimurium or left uninfected as controls for 10 h. Following magnetic bead-based enrichment of intracellular bacteria from infected cultures, anti-Flag immunoprecipitation was performed. Total lysates, enriched SCV fractions, and immunoprecipitated complexes from both groups were separated by SDS‑PAGE and visualized with silver staining. **B** Analysis of the Glut1 interactome by mass spectrometry. Immunoprecipitated proteins were processed for LC‑MS/MS. Scatter plot displaying protein abundance ratios (BA‑Glut1/naive Glut1 spectral counts) versus relative intensity. The dashed line indicates a twofold enrichment threshold. Proteins with ≥ 2 total spectral counts and a BA‑GLUT1/native GLUT1 ratio ≥ 2 were defined as preferentially associated with BA‑GLUT1. Elevated ratios reflect preferential enrichment in BA‑Glut1 samples. **C** Validation of Glut1-Rab1/Rab5 association by immunoblotting. **D** Densitometric quantification from (**C**). **E–F** Co-localization of Glut1 with SNARE proteins during *S*. Typhimurium infection.** E** HeLa cells stably expressing EGFP‑tagged SNARE proteins were infected with *S*. Typhimurium for 2 h and processed for immunofluorescence. Representative images show the distribution of the indicated SNARE (green), endogenous Glut1 (red), and nuclei (blue). Scale bars: 10 µm (overview), 2 µm (magnified regions). White arrows denote BA‑Glut1 signals. **F** Quantification of SNARE and GLUT1 co‑localization. Data are derived from ≥ 30 cells per condition in three independent experiments. The EGFP‑only group was used as a negative control. **G**-**H** Effects of BFA and nocodazole on Glut1 association with SCVs and glucose uptake activity of SCVs. **G** RAW264.7 cells were infected with *S.* Typhimurium for 16 h, with indicated inhibitors added 1 h post-infection. Following magnetic bead-based enrichment of intracellular SCV, Glut1 levels were assessed by immunoblotting (left) and quantified by densitometry (right). **H** Glucose uptake activity of isolated SCVs from infected cells. Data are expressed as fold change relative to the DMSO control (set as 1). For all panels, representative immunoblots from three independent experiments are shown. Bar graph data represent mean ± SD. Statistical significance was determined by Student's *t*-test (*p < 0.05, **p < 0.01)

To elucidate the molecular mechanism underlying Glut1 association with SCVs, we employed the same nanoparticle-coated magnetic strategy (Fig. 4E) and compared the interactomes of bacteria-associated GLUT1 (BA-GLUT1, i.e., Glut1 localized on SCVs) and native GLUT1, isolated from *S*. Typhimurium-infected and uninfected 293 T cells, respectively. After immunoaffinity enrichment, a range of protein bands was detected by silver staining (Fig. 5A). Based on spectral count ratios (BA-GLUT1/native GLUT1), MS analyses revealed quantitatively distinct interaction profiles for BA-GLUT1 (Fig. 5B and Supplementary Table 1). Using a threshold of ≥ twofold enrichment (BA-GLUT1/native GLUT1 spectral count ratio) and filtering for proteins with ≥ 2 total spectral counts, we found that BA-GLUT1 showed preferential association with multiple components of the host vesicular trafficking system, including small GTPases (particularly Rab family members), sorting nexins (SNXs), vacuolar protein sorting (VPS) factors, and various membrane-integrated proteins (Fig. 5B). Western blot validation confirmed significant enrichment of Rab5A and Rab1A in BA-GLUT1 complexes (Figs. 5C-D), while immunofluorescence demonstrated colocalization between GLUT1 and SNARE proteins VAMP8/Vti1b during infection (Figs. 5E-F).

To functionally validate the requirement of vesicular trafficking in Glut1 association with SCVs, we disrupted this pathway in RAW264.7 macrophages using brefeldin A (BFA) and nocodazole (Noco). While BFA inhibits anterograde transport from the ER to the Golgi and disrupts endosomal recycling, nocodazole primarily depolymerizes microtubules to inhibit long-distance vesicular transport. Interestingly, BFA treatment, but not nocodazole, substantially reduced Glut1 association with SCVs (Fig. 5G). However, both inhibitors concurrently impaired the glucose uptake capacity of the SCV compartment, as evidenced by significantly attenuated 2DG6P accumulation (Fig. 5H). The inhibition of glucose uptake by nocodazole is consistent with previous reports that it can directly suppress Glut transport activity through a microtubule-independent mechanism [37]. These results collectively demonstrate that BFA-sensitive vesicular trafficking contributes to mediating Glut1 association with the bacterial vacuole in murine macrophages.

### K29-linked ubiquitination of BA-GLUT1 enhances glucose acquisition by intravacuolar *S.* Typhimurium

**Fig. 6:**
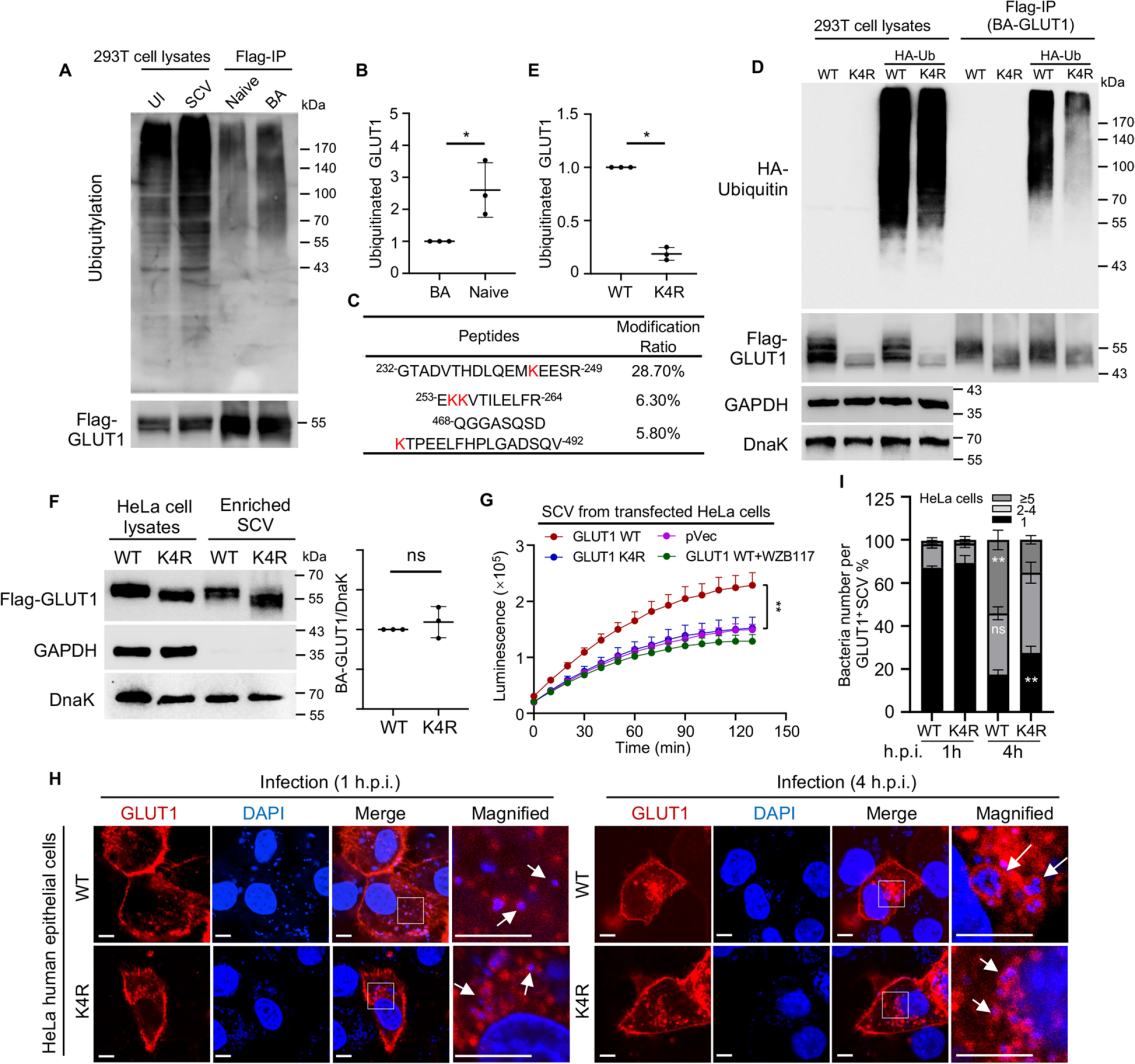
Ubiquitination of BA-Glut1 facilitates glucose acquisition by intravacuolar *S*. Typhimurium within host cells. **A**-**C** Ubiquitination of BA-GLUT1. **A** Immunoblot detection of BA-GLUT1 ubiquitination. **B** Densitometric quantification of ubiquitinated GLUT1 in (**A**). **C** Mass spectrometry identification of four lysine ubiquitination sites (K245, K255, K256, and K477) on BA-GLUT1. Relative modification levels are provided in Supplementary Fig. 6. **D**-**E** Mutation of the four conserved lysine residues suppresses BA-GLUT1 ubiquitination. 293 T cells were co-transfected with HA‑ubiquitin and Flag-tagged wild-type GLUT1 or the quadruple K4R mutant. After 18 h of transfection, cells were infected with *S*. Typhimurium for 10 h. BA-GLUT1 was magnetically enriched, immunoprecipitated with anti-Flag antibody, and subjected to ubiquitination analysis. **D** Representative immunoblots. **E** Densitometric quantification of relative ubiquitination levels. **F**-**G** The K4R GLUT1 mutation does not alter GLUT1 association with SCVs but impairs intravacuolar *S*. Typhimurium glucose uptake. HeLa cells expressing wild-type GLUT1 or the K4R mutant were infected with *S*. Typhimurium for 10 h, followed by magnetic purification of SCVs. **F** Immunoblotting and quantitative analysis of GLUT1 abundance in purified SCVs. **G** Glucose uptake activity was measured directly in isolated SCV fractions. SCVs purified from empty vector-transfected cells and WZB117‑treated cells were used as independent negative controls. **H-I** The K4R GLUT1 mutation reduces the bacterial numbers within individual SCVs. **H** HeLa cells expressing RFP-tagged WT GLUT1 or K4R mutant were infected with *S*. Typhimurium at MOI = 10 for 1 h or 4 h. Representative confocal images show the transfected GLUT1 (red) and nuclei (blue). White boxes highlight magnified regions, and arrows mark GLUT1-positive SCVs. Scale bar, 10 μm. **I** Quantification of bacterial numbers within each GLUT1-positive SCV. Data from three independent experiments (more than 50 cells per group) are expressed as a percentage of total GLUT1-positive SCVs. For all panels, representative immunoblots from three independent experiments are shown. In panels A and D, values represent the relative ubiquitination levels normalized to Flag-tagged BA-GLUT1.All quantitative data are presented as mean ± SD. Statistical analysis was performed using Student’s *t*-test (*p < 0.05, **p < 0.01)

Ubiquitination has been reported to play roles in the stability and subcellular localization of GLUT1 [32, 38, 39]. Immunoblot analysis revealed significantly enhanced ubiquitination of BA-GLUT1, suggesting *S*. Typhimurium infection induces additional ubiquitin modifications on GLUT1 (Fig. 6A-B). Quantitative mass spectrometry identified four novel ubiquitinated peptides in BA-GLUT1 that were absent in native GLUT1. Notably, the peptide ^232^GTADVTHDLQEMKEESR^249^ exhibited robust ubiquitination (28.7% modification rate), while other modified peptides showed less than 10% modification rates. MS/MS spectral analysis precisely mapped the ubiquitination sites to K245, K255, K256, and K477 (Fig. 6C and Supplementary Figs. 6A-E). Transfection experiments in 293 T cells revealed that the K245R/R255R/K256R/K477R mutation (K4R) showed significantly reduced ubiquitination signals of BA-GLUT1 (Fig. 6D-E). These lysines are located within the accessible cytoplasmic region of GLUT1 and are evolutionary conserved among mammalian hosts of *Salmonella* (human, mouse, pig, chicken) but not across the GLUT family (Supplementary Figs. 6F-G**)**.

We first examined whether the K4R mutation affects GLUT1 localization. In uninfected HeLa epithelial cells, the K4R mutant showed normal subcellular localization comparable to wild-type GLUT1 (Supplementary Fig. 7). During *S*. Typhimurium infection, the K4R mutant retained normal association with SCVs, as confirmed by immunoblot analysis of purified SCVs (Fig. 6F) and immunofluorescence staining (Fig. 6H). We next assessed the functional impact of the K4R mutation. The K4R mutant exhibited markedly reduced 2DG6P accumulation in purified SCV lumens compared to wild-type GLUT1 (Fig. 6G). At 1-h post-infection, bacteria per GLUT1-positive SCV was comparable between WT and K4R, indicating that the K4R mutation does not affect initial invasion. However, at 4-h post-infection, the K4R mutation reduced bacterial loads within SCVs, as shown by a shift toward fewer bacteria per GLUT1-positive SCV (Figs. 6H-I). Collectively, these results demonstrate that the K4R mutation impairs intravacuolar bacterial replication in HeLa epithelial cells without affecting initial bacterial uptake or SCV association of GLUT1.

**Fig. 7:**
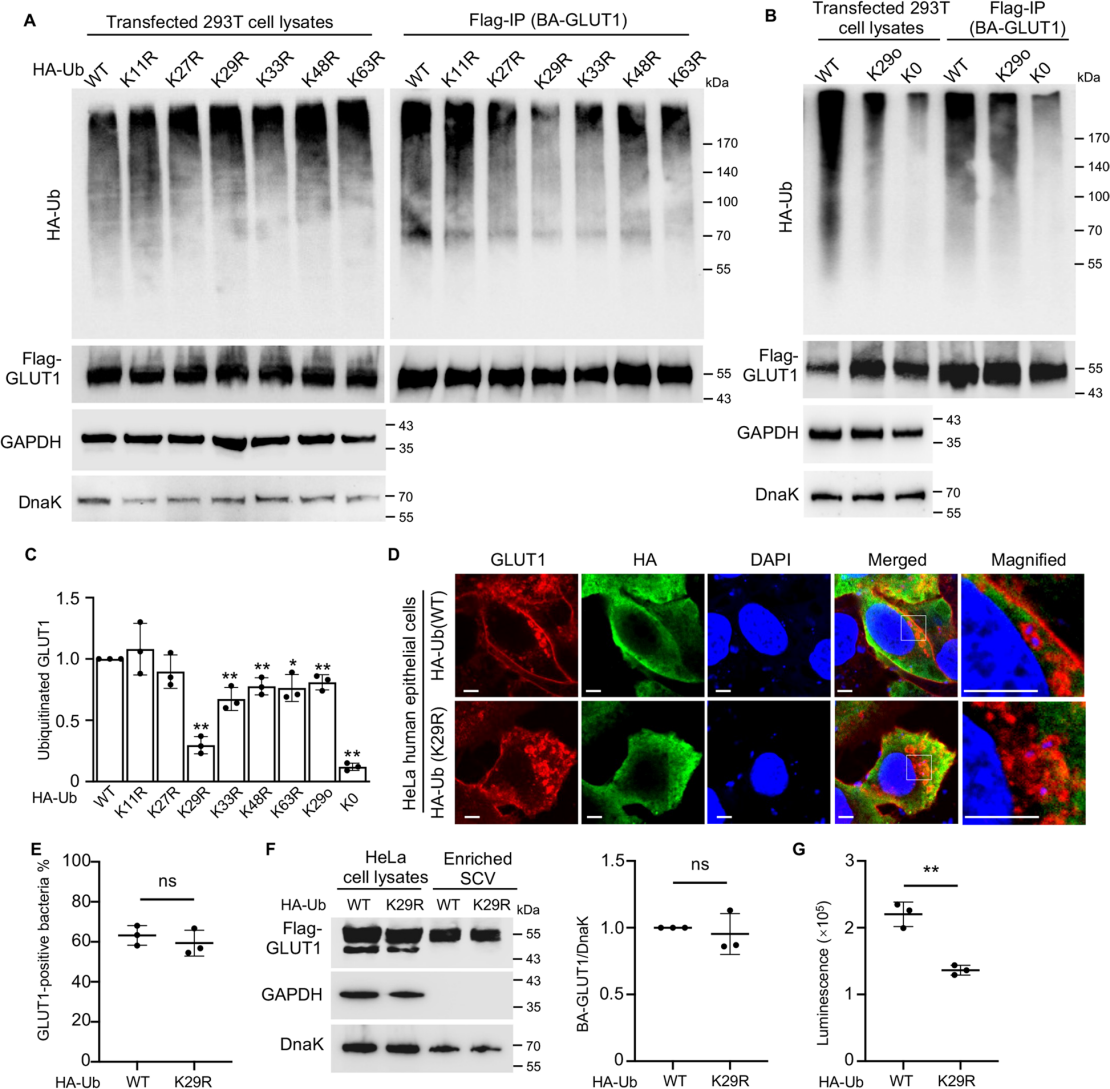
K29-linked ubiquitination of BA-Glut1 facilitates glucose acquisition by intravacuolar *S*. Typhimurium within host cells. **A**-**C** BA-GLUT1 undergoes preferential K29-linked ubiquitination. 293 T cells were transfected with Flag-GLUT1 together with WT HA-ubiquitin or a panel of ubiquitin variants, including single-lysine mutants (**A**), lysine-null K0, and K29-only mutant (**B**). At 18 h post-transfection, cells were infected with *S*. Typhimurium. Intravacuolar bacteria were magnetically enriched, followed by anti-Flag immunoprecipitation and immunoblotting with the indicated antibodies. **C** Densitometric quantification of relative GLUT1 ubiquitination levels normalized to the WT-Ub group. **D**-**E** The K29R Ub mutation does not alter GLUT1 localization with intracellular *S*. Typhimurium. HeLa cells expressing WT Ub or the K29R mutant were infected with *S*. Typhimurium at MOI = 10 for 2 h. (D) Representative confocal immunofluorescence images show endogenous GLUT1 (red), Ub (green), and cell nuclei (blue). (E) Quantification of the percentage of GLUT1-positive bacteria. Data from three independent experiments (more than 50 cells per group) are expressed as a percentage of total intracellular bacteria. Scale bar, 10 µm. **F-G** The K29R Ub mutation impairs intravacuolar *S*. Typhimurium glucose uptake. HeLa cells were co-transfected with Flag-GLUT1 together with WT Ub or the K29R Ub mutant, then infected with *S*. Typhimurium at MOI = 100 for 10 h, followed by magnetic purification of SCVs. **F** Immunoblotting and quantitative analysis of GLUT1 abundance in purified SCVs. **G** Glucose uptake activity was measured directly in isolated SCV fractions. For all panels, representative immunoblots from three independent experiments are shown. In panels A and B, values represent the relative ubiquitination levels normalized to Flag-tagged BA-GLUT1. Bar graphs display mean ± SD. Statistical significance was determined by Student's *t*-test (*p < 0.05, **p < 0.01)

To determine the ubiquitin chain linkage type, we performed transfection experiments in 293 T cells using a panel of lysine-to-arginine Ub mutants. The ubiquitination signal of BA-GLUT1 was markedly decreased in the presence of K29R or K0 (lysine-null) Ub mutants, whereas WT Ub and other single-lysine mutants (K11R, K27R, K48R, etc.) had no such significant effect. Strikingly, a K29-only (K29o) Ub mutant (retaining solely K29) largely restored ubiquitination, indicating that BA-GLUT1 undergoes selective K29-linked polyubiquitination (Figs. 7A-C). We next asked whether K29-linked ubiquitination enhances GLUT1 transport activity or promotes its association with SCVs. To address this, we expressed WT or K29R GLUT1 in HeLa cells and performed functional assays. Immunofluorescence analysis at 4 h post-infection showed that the K29R mutation did not alter GLUT1 localization to SCVs (Figs. 7D-E). Consistently, immunoblot analysis of purified SCVs at 4 h.p.i. confirmed that K29R did not significantly affect GLUT1 association with SCVs compared to wild-type GLUT1 (Fig. 7F). In contrast, when glucose uptake was measured directly in purified SCVs, the K29R mutation significantly reduced SCV glucose uptake activity (Fig. 7G). These results indicate that K29-linked ubiquitination enhances GLUT1 transport activity on SCVs rather than altering its association with SCVs. Collectively, our data suggest that GLUT1 on SCVs facilitates glucose acquisition by the intravacuolar bacteria within host cells via its K29-conjugated ubiquitination.

### Glut1 inhibition restricts the intracellular replication of *S.* Typhimurium alongside enhanced host immune responses and reprogrammed bacterial metabolism

**Fig. 8:**
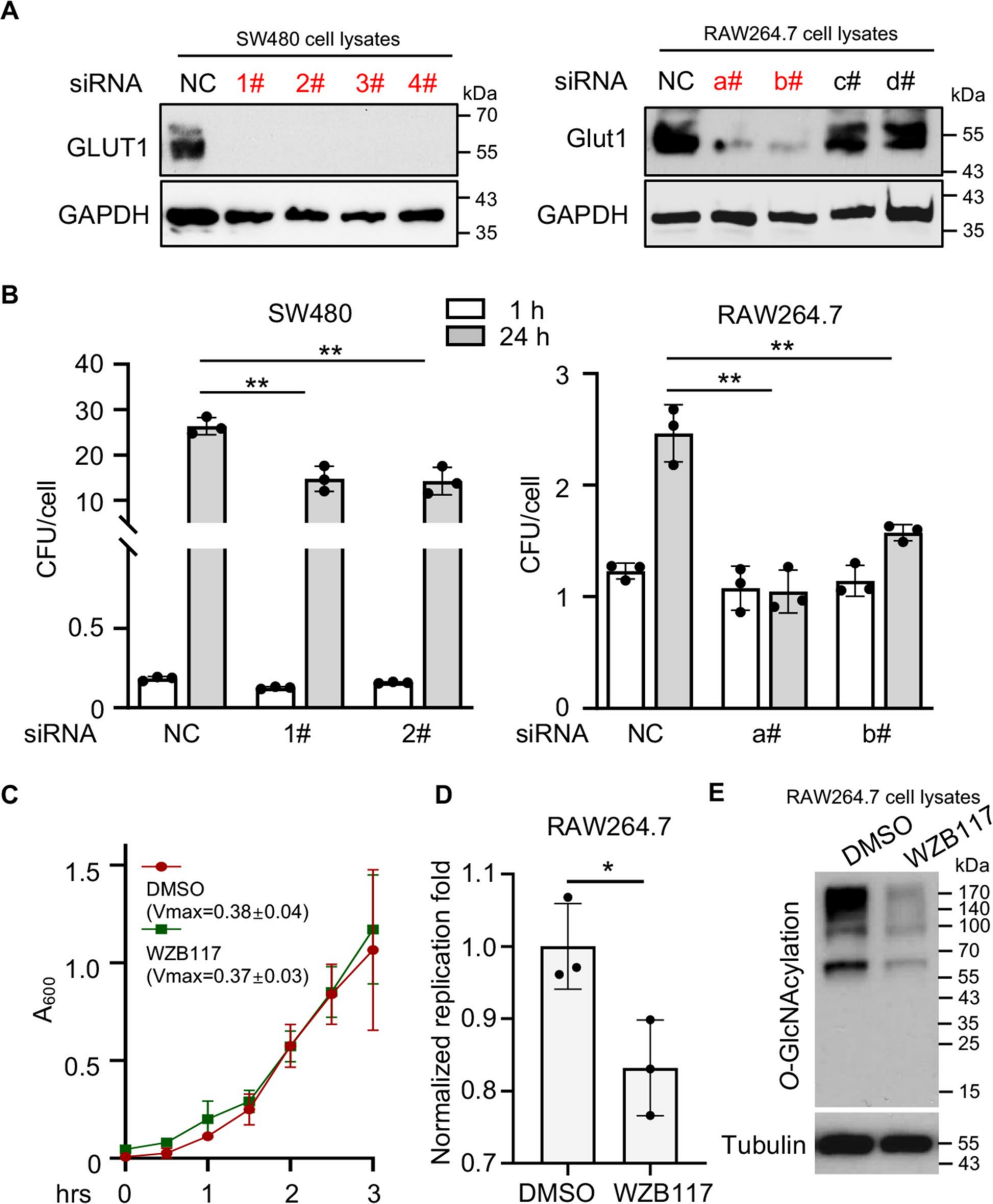
Glut1 contributes to *S*. Typhimurium infection in host cells. **A**-**B** Effects of Glut1 knockdown on intracellular replication of *S.* Typhimurium. RAW264.7 and SW480 cells were transfected with Glut1-targeting siRNA (mouse-specific siRNAs #a-d for RAW264.7, human-specific siRNAs #1–4 for SW480) for 48 h and subsequently infected with *S.* Typhimurium at an MOI of 10. **A** Immunoblot validation of Glut1 knockdown efficiency. **B** Quantification of intracellular bacteria by serial dilution and plating. Intracellular bacterial loads were assessed by comparing CFUs at 1 and 24 h post-infection, normalized to host cell number. **C** Effect of WZB117 treatment on *S*. Typhimurium growth kinetics in LB broth. Overnight cultures of *S.* Typhimurium were diluted 1:33 in LB broth containing either DMSO (control) or WZB117, and bacterial growth was monitored by measuring optical density. The maximum growth rate (Vmax) is indicated. **D**-**E** Effects of WZB117 treatment on the replication of *S*. Typhimurium in macrophages. **D** RAW264.7 cells were infected with *S*. Typhimurium for 1 h, followed by treatment with WZB117. After 24 h of infection, cells were lysed, and intracellular bacteria were enumerated. Fold replication was calculated as the ratio of bacterial counts at 24 h to those at 1 h post-infection, normalized to host cell number, and then normalized to the DMSO control (set as 1). **E** Immunoblot analysis of global *O*-GlcNAcylation levels confirms the efficacy of WZB117. Results shown are mean values ± SD from three independent experiments. Statistical significance was determined by Student's *t*-test (*p < 0.05, **p < 0.01)

To investigate the role of Glut1 in *S*. Typhimurium replication, we first silenced Glut1 expression using two effective siRNA pairs in SW480 intestinal epithelial cells and RAW264.7 macrophages (Fig. 8A). Given that Glut1 inhibition induced a slight reduction in host cell proliferation (Supplementary Fig. 8), we normalized all intracellular bacterial counts to host cell number to ensure quantification. While *Glut1* knockdown did not affect bacterial invasion (1 h post-infection), it significantly decreased *S*. Typhimurium replication at 24 h in both cell types (Fig. 8B). This phenotype was recapitulated using the Glut1-specific inhibitor WZB117, which suppressed bacterial proliferation in murine macrophages without affecting growth in LB medium (Fig. 8C-D), indicating that Glut1 is a host factor that supports *S*. Typhimurium intracellular replication. The functionality of WZB117 was confirmed by detecting total *O*-GlcNAcylation in cell lysates (Fig. 8E).

**Fig. 9:**
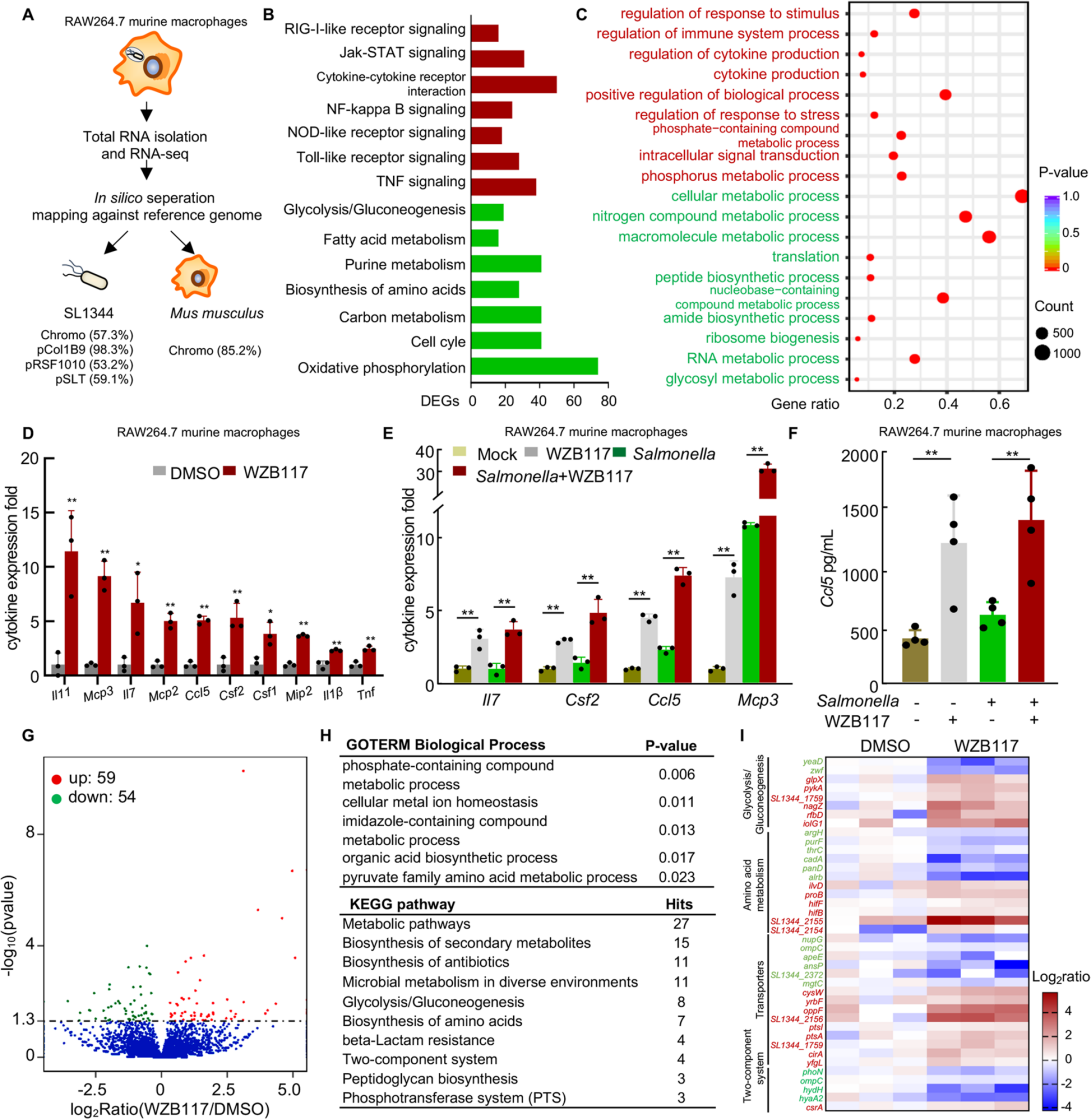
Glut1 inhibition enhanced host immune responses and reprogrammed bacterial metabolism. RAW264.7 macrophages were infected with *S*. Typhimurium for 1 h, followed by treatment with WZB117 for an additional 10 h prior to analyses. **A** Schematic of the dual RNA-seq strategy in *S*. Typhimurium-infected macrophages. Read mapping statistics (expressed as percentages) show the genomic and plasmid coverage distribution. **B**-**C** Host transcriptomic responses to Glut1 inhibition during infection. Pathway enrichment analysis of differentially expressed genes (DEGs, WZB117 vs DMSO) was performed through (**B**) Gene Ontology and (**C**) KEGG using the DAVID online software. Bar length and circle size represent the number of DEGs enriched in each pathway. Pathways in red indicate upregulation, while those in green indicate downregulation in response to WZB117 treatment. **D**-**F** Effects of Glut1 inhibition on cytokine production during *S*. Typhimurium infection of RAW264.7 macrophages. **D** Transcriptomic profiling of cytokine expression during *S*. Typhimurium infection. **E** qPCR validation of cytokine mRNA levels. **F** ELISA quantification of CCL5 secretion. Results shown are mean values ± SD from at least three independent experiments. Statistical significance was determined by Student's *t*-test (*p < 0.05, **p < 0.01). **G**-**I** Effects of Glut1 inhibition on *S*. Typhimurium SL1344 gene expression. **G** Volcano plot analysis of differentially expressed bacterial genes (WZB117 vs DMSO). **H** Functional enrichment analysis of transcriptional changes of *S*. Typhimurium. **I** Heatmap of differentially expressed bacterial genes involved in the major biological processes. Red labels correspond to the upregulated DEGs, and green labels correspond to the downregulated DEGs

To investigate the role of Glut1 in host-*S*. Typhimurium interactions, we employed the WZB117 combined with dual RNA-seq to simultaneously profile transcriptomic changes in both host and bacterial cellular compartments in RAW264.7 murine macrophages (Fig. 9A). Host transcriptome sequencing (85.2% chromosomal coverage) revealed that WZB117-mediated Glut1 inhibition broadly altered host cell processes. Pathway analysis showed that WZB117 treatment significantly suppressed multiple metabolic pathways, including glycolysis/gluconeogenesis, fatty acid metabolism, and amino acid metabolism, while concurrently activating innate immune signaling pathways such as RIG-I-like receptor, NF-κB, NOD-like receptor, and TNF signaling (Figs. 9B-C). This immune activation was accompanied by substantial upregulation of pro-inflammatory cytokine transcripts (Fig. 9D). We selected key mediators from this signature and confirmed their enhanced expression in RAW264.7 murine macrophages through orthogonal methods, validating increased mRNA levels by qPCR and elevated protein secretion by ELISA (Figs. 9E-F).

For the bacterial transcriptome, sequencing achieved coverages of 57.3% for the chromosome, 98.3% for pCol1B9, 53.2% for pRSF1010, and 59.1% for pSLT, identifying 59 upregulated and 54 downregulated genes (Fig. 9A and G). Parallel bacterial profiling revealed that WZB117 treatment induced significant alterations in *S*. Typhimurium metabolic pathways, particularly affecting central carbon metabolism, amino acid metabolism, membrane transport, and two-component regulatory systems. The gene expression profile reflected characteristic adaptations to glucose deprivation, including upregulation of gluconeogenesis genes *glpX* and *pykA* (Figs. 9H-I). These findings demonstrate that Glut1 serves as both a critical metabolic hub supporting *S*. Typhimurium intracellular replication and a key suppressor of host anti-*S*. Typhimurium immunity.

### Glut1 inhibition enhances *S.* Typhimurium-induced immune responses and reduces bacterial burden in vivo

**Fig. 10:**
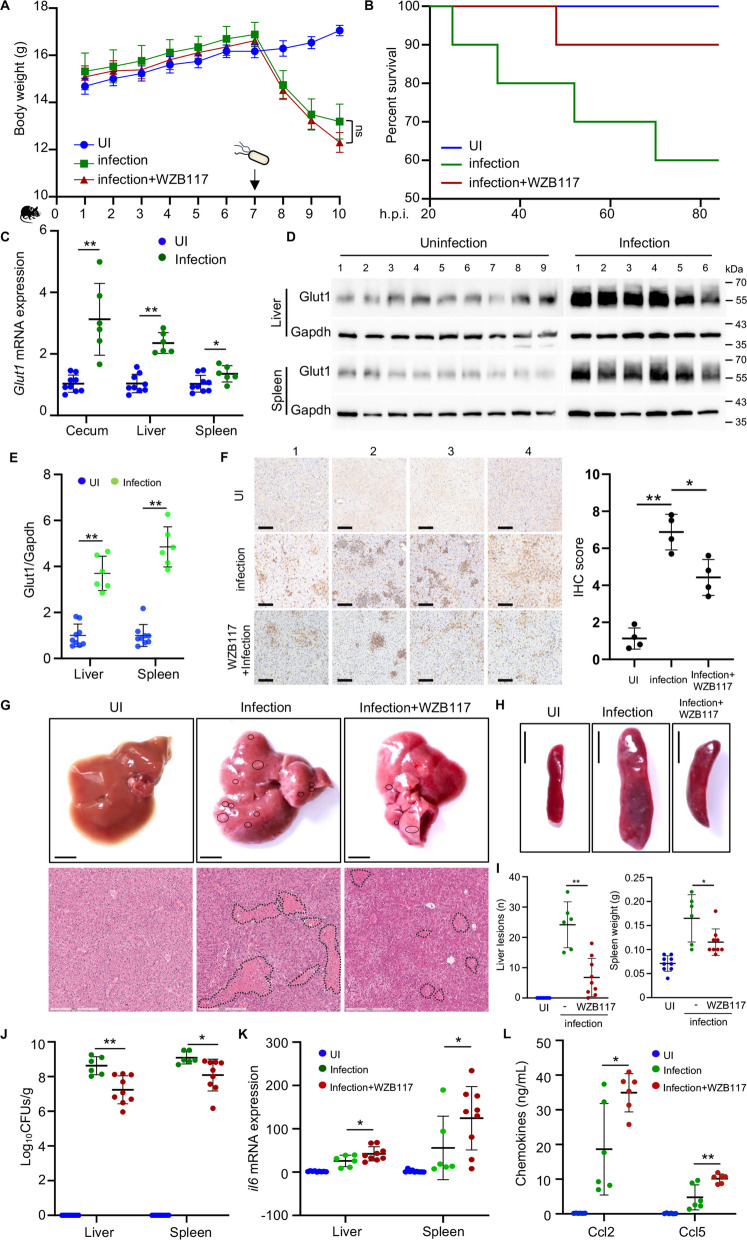
Glut1 inhibition enhances immune responses and reduces bacterial burden during *S.* Typhimurium infection in vivo. **A** Experimental procedure of *S*. Typhimurium infection and pharmacological intervention in vivo. C57BL/6 mice (*n* = 10 per group) were administered daily intraperitoneal injections of WZB117 for 7 consecutive days prior to infection with *S*. Typhimurium. Body weight was monitored daily throughout the study. Mice surviving to 82 h post-infection were euthanized for subsequent tissue collection and analysis. **B** Survival curves of *S*. Typhimurium -infected mice for 82 h post-infection. **C**-**F** The effects of *S*. Typhimurium infection on the Glut1 expression. **C** *Glut1* mRNA levels in the cecum, liver, and spleen were measured by qPCR. Each data point represents the mean value from three technical replicates per mouse. All data are expressed as fold change relative to the uninfected control group. **D** Glut1 protein levels in the liver and spleen were analyzed by immunoblotting. During immunoblotting, the infected and uninfected samples were processed in parallel in the same run. Both membranes were processed simultaneously under identical conditions (blocking, washing, antibody incubation, and development) to ensure comparability. **E** Densitometric quantification of GLUT1 protein levels in (**D**), with the uninfected control group normalized to 1. **F** Immunohistochemical analysis of Glut1 expression in the liver from four randomly selected mice. Representative IHC images of Glut1 were shown (left), and the IHC scores were calculated (right). Scale bar, 100 µm. **G**-**I** The effects of Glut1-inhibition on the *S*. Typhimurium-induced hepatic and splenic pathology. **G** Shown are the representative images of liver gross morphology (top, scale bar, 2 mm) and corresponding H&E staining (bottom, scale bar, 300 µm). Boxed areas indicate necrotic lesions. **H** Shown are representative images of spleen gross morphology. **I** Quantification of the hepatic and splenic pathological damage. **J** The effects of Glut1 inhibition on bacterial colonization. Bacterial counts in the liver and spleen were calculated as CFUs per gram of tissue. **K**-**L** The effects of Glut1-inhibition on the inflammatory response in *S*. Typhimurium-infected mice. **I** *Il6* mRNA levels were quantified by qPCR. Serum concentrations of Ccl2 and Ccl5 were measured by ELISA. All data are presented as mean ± SD from at least four biological replicates. Statistical significance was determined using Student's t-test (*p < 0.05, **p < 0.01)

To investigate the role of Glut1 during *S*. Typhimurium infection in vivo, we established a murine model pretreated intraperitoneally with the Glut1 inhibitor WZB117 for seven days before infection. Body weight changes and survival rates were monitored over 82 h post-infection (Figs. 10A-B). Infection with *S*. Typhimurium significantly increased *Glut1* mRNA levels in intestinal cecum tissues, liver, and spleen (Fig. 10C). Consistently, immunoblotting and immunohistochemistry confirmed a substantial upregulation of Glut1 protein expression in the infected liver and spleen (Figs. 10D-E). WZB117 pretreatment improved survival outcomes (Fig. 10B), attenuated infection-induced Glut1 upregulation in the liver (Figs. 10F), and was associated with reduced *S*. Typhimurium-induced tissue damage, reflected by reduced necrotic lesions in the liver and attenuated splenomegaly (Figs. 10G-I). These protective outcomes were associated with a significant decrease in bacterial load, demonstrated by reduced *Salmonella* colonization in the liver and spleen (Fig. 10J).

Consistent with our in vitro observations, Glut1 inhibition enhanced host immune responses in vivo, as shown by elevated *Il6* mRNA levels in infected tissues (Fig. 10K) and increased serum concentrations of chemokines (Fig. 10L). Taken together, these results demonstrate that Glut1 inhibition restricts bacterial burden in vivo and is associated with enhanced host immune responses and reduced tissue damage.

## Discussion

**Fig. 11:**
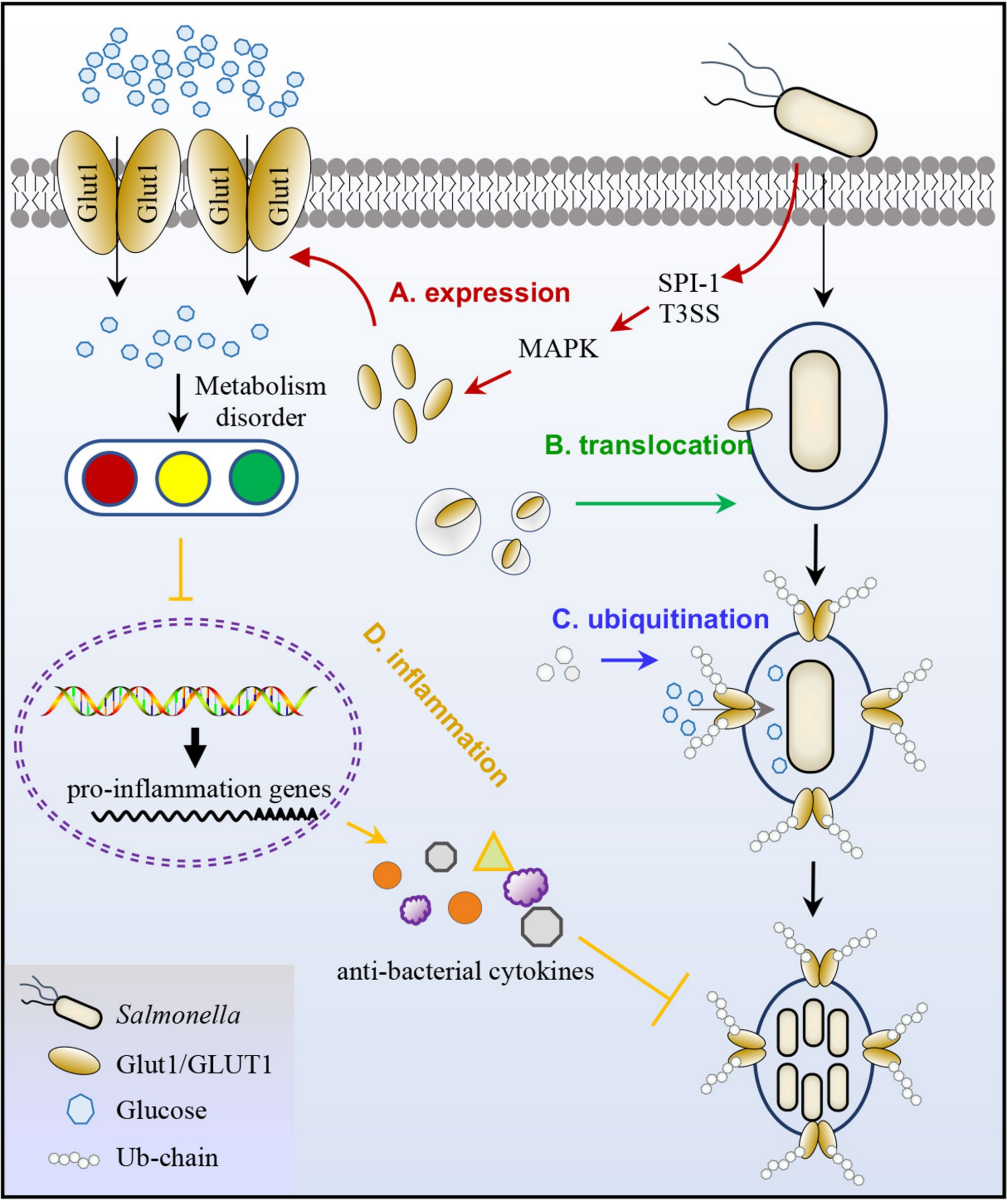
The schematic diagram of this work. This model unveils how *S*. Typhimurium synchronizes host manipulation of Glut1 across transcriptional, spatial, and post-translational levels to optimize its intracellular niche. **A** Transcriptional upregulation (red). The SPI-1 T3SS upregulates GLUT1 expression via MAPK-mediated transcriptional activation, enhancing glucose uptake of host cells. **B** Spatial localization (green). Glut1 is associated with the SCV via cellular trafficking, enabling intravacuolar *S*. Typhimurium to access host-derived glucose. **C** PTM-mediated activation (blue). *S*. Typhimurium induces K29-linked ubiquitination of Glut1, boosting its glucose transport activity. **D** Immune suppression (orange). Manipulation of Glut1 by *S*. Typhimurium reprograms host glucose metabolism, potentially dampening immune response through mechanisms yet to be defined

The strategy whereby intracellular pathogens co-opt host nutrient receptors for survival epitomizes the evolutionary arms race between microbes and their hosts. Our study uncovers an unrecognized mechanism by which *S*. Typhimurium ensures intracellular survival through coordinated regulation of host Glut1. First, the SPI-1 T3SS upregulates Glut1 expression via MAPK signaling, enhancing glucose uptake to fuel bacterial replication. Second, *S*. Typhimurium facilitates GLUT1 localization to the SCV membrane via BFA-sensitive host trafficking machinery. Concurrently, *S*. Typhimurium induces K29-linked ubiquitination of Glut1, further enhancing its glucose transport activity. GLUT1 inhibition not only disrupts *S*. Typhimurium metabolic adaptation but also potently enhances anti-*S*. Typhimurium innate immune responses, highlighting Glut1 as both a metabolic vulnerability and an immunoregulatory node during infection. These findings provide novel mechanistic insights into microbial pathogenesis, illustrating how an intracellular pathogen can simultaneously manipulate host biology across multiple levels (transcriptional regulation, spatial protein trafficking, and activity modulation through post-translational modifications) to support its intracellular niche establishment (Fig. 11).

### An SPI-1-MAPK regulatory mechanism of Glut1 expression during *S*. Typhimurium infection

Glut1 expression is finely tuned by various environmental stressors, including nutrient deprivation, hypoxia, and LPS stimulation [40–42]. This regulation is controlled by a complex molecular network, encompassing transcriptional activation via binding of factors (e.g., NF-κB and HIF-1α) to the Glut1 promoter [43, 44], post-transcriptional modulation through miRNA-mediated mRNA destabilization (e.g., miR-320a/miR-328-3p interaction with the UTR region via RNA-induced silencing complex (RISC)) [45, 46], and post-translational control of protein turnover via ubiquitination-mediated proteolysis facilitated by specific E3 ligases [38, 39]. In this study, we show that Glut1 upregulation during *S*. Typhimurium infection occurs through a mechanism distinct from these canonical pathways. In RAW264.7 murine macrophages, this upregulation occurs independently of changes in mRNA stability or protein degradation. Moreover, it is not directly mediated by HIF-1α/NF-κB signaling or LPS stimulation, as LPS alone induces only modest Glut1 expression compared to intact bacterial infection. Instead, this process depends on the SPI-1 T3SS and involves a previously uncharacterized MAPK-dependent regulatory mechanism. Overexpression of the SPI-1 T3SS effectors SopB/E/E2 enhances Glut1 levels while concurrently activating MAPK signaling cascades, indicating that these effectors function beyond cytoskeletal manipulation to modulate host metabolic regulation.

Interestingly, while our data show that *S*. Typhimurium can activate MAPK signaling to enhance Glut1 expression, the bacterial effector SpvC is known to irreversibly inactivate MAPKs [47]. We therefore propose a temporal coordination model to reconcile these seemingly opposing functions. Immediately following bacterial invasion, host pattern recognition receptors or SPI-1 T3SS effectors initiate an early wave of MAPK activation [31], which promotes Glut1 expression and metabolic reprogramming to support initial bacterial survival. However, sustained MAPK activation could lead to excessive inflammation or host cell death. In contrast, at later stages, the SPI-2 T3SS effector SpvC acts as a phosphothreonine lyase to directly inactivate MAPKs [47, 48]. This attenuation represses pro-apoptotic and pro-inflammatory gene expression, limiting host cell death and inflammatory responses and thereby fostering a permissive niche for bacterial replication [49–51]. Thus, the net effect on MAPK signaling varies over time: early activation followed by SpvC-mediated suppression. Our western blot results support this model, showing higher JNK and ERK activation at 2 h than at 6 h and 10 h post-infection (Figs. 2B-C). This spatiotemporal coordination of opposing effector functions may represent a refined strategy by which pathogens fine-tune host responses to favor their survival.

### Glut1 association with SCVs and its ubiquitination induced by *S*. Typhimurium for vacuolar nutrient supply

Although pathogens residing within membrane-bound vacuoles can evade cytosolic antimicrobial peptides and immune responses, this isolation also cuts off their access to host nutrients. Pathogens therefore require co-option of host nutrient transport systems to sustain their survival and replication. While extensive research has focused on pathogen interactions with the host endolysosomal system (e.g., RAB proteins, SNAREs) to establish and stabilize replication niches [52, 53], the mechanisms by which intravacuolar pathogens selectively recruit host nutrient transporters remain poorly understood.

To our knowledge, only a limited number of examples have been reported. *Legionella* recruits the host amino acid transporter SLC1A5 to the *Legionella*-containing vacuole (LCV) to facilitate amino acid acquisition [54], and *Chlamydia* co-opts the nucleotide-sugar transporter SLC35D2 to import UDP-glucose for bacterial glycogen synthesis [55]. Although previous work has shown that *Salmonella* recruits host solute carriers SLC1A5, SLC3A2, and SLC7A5 to the SCV membrane, where they help mobilize mTOR signaling and evade host autophagy [56], it remains unclear whether these transporters directly contribute to nutrient acquisition by the intravacuolar bacteria. In this study, we identify a previously uncharacterized mechanism through which *S*. Typhimurium specifically promotes the association of the glucose transporter Glut1 with the SCV. We show that *Glut1* knockdown severely impairs glucose import into the SCV, supporting that Glut1 on SCV contributes to bacterial intravacuolar nutrient acquisition. The localization of Glut1 to the SCV appears to depend on host vesicular trafficking machinery, as demonstrated by significantly impaired Glut1 localization upon BFA treatment. Consistent with this finding, our interactome analysis reveals that Glut1 specifically associates with multiple components of the vesicular trafficking system during infection. We thus propose that Glut1 becomes incorporated into the SCV membrane via the host endosomal trafficking pathways that *S*. Typhimurium hijacks for SCV biogenesis and maintenance, including Rab GTPase-mediated vesicular transport and SNARE-dependent membrane fusion events [52, 53].

Although the precise topological orientation of Glut1 within the SCV membrane remains technically challenging to resolve, its fundamental transport mechanism provides valuable functional insight. As a facilitative glucose transporter, Glut1 mediates bidirectional, ATP-independent glucose movement down concentration gradients [21]. Consequently, irrespective of its membrane orientation, the SCV can utilize Glut1 to acquire glucose from the host cytosol. This model is further supported by our observations that Glut1 expression is upregulated during infection and coincides with increased glucose uptake into the host cell..

In addition to transporter associations, we observed that *S*. Typhimurium induces K29-linked ubiquitination of SCV-localized Glut1. While Glut1 ubiquitination has previously been implicated in modulating protein stability or intracellular trafficking [32], we find a distinct pathogen-driven ubiquitination event that targets lysine residues in cytosolic loops and enhances glucose transport activity without altering protein turnover or subcellular localization. The precise mechanism remains unclear. One possibility is that *Salmonella* promotes Glut1 ubiquitination via its own E3 ligases (e.g., SspH, SopA, SlrP) or by activating host E3 ligases. Another possibility is that *Salmonella* suppresses host deubiquitinases (DUBs) to limit Glut1 deubiquitylation, stabilizing its ubiquitinated state. Notably, *Legionella pneumophila* inhibits the host DUB OTUB1 via effectors [57], and *Salmonella* effectors such as SopE/E2 manipulate host DUB activity to remodel vacuolar dynamics [58]. It is therefore plausible that *S*. Typhimurium may employ a dual strategy (enhancing ubiquitination while inhibiting deubiquitination) to promote Glut1 retention and function at the SCV membrane, although this hypothesis awaits experimental validation.

*Salmonella* can occupy other intracellular compartments, including the cytosol of epithelial cells and a dormant/persister state in macrophages [59, 60]. Although we examined the effect of *Salmonella* on Glut1 expression and the role of Glut1 in bacterial replication and virulence at the population level, our study focused on the localization and ubiquitination of Glut1 for intravacuolar *S*. Typhimurium. Future studies investigating the role of Glut1 across different infection stages and cell types are needed to further clarify these potential niche-specific differences.

### *S*. Typhimurium hijacks Glut1 to evade host immune defenses

Glut1 is well-established as a proinflammatory regulator that modulates both innate and adaptive immune responses. In innate immunity, it promotes macrophage polarization toward the M1 phenotype, activates NF-κB signaling, and stimulates the production of proinflammatory cytokines, including TNF-α, IL-6, and IL-1β [61]. Within adaptive immunity, Glut1 enhances T cell activation and effector functions (such as IFN-γ and TNF-α secretion) while facilitating B cell differentiation into antibody-producing plasma cells [62, 63].

The precise mechanisms by which Glut1 regulates anti-pathogen immunity, especially against intracellular pathogens, remain elusive due to the intricate nature of host–pathogen interactions. Glut1 exacerbates lung fibrosis through AIM2 inflammasome activation during *Streptococcus pneumoniae* infection and is essential for monocyte inflammatory responses via HIF-1α-mediated glycolysis in *Treponema pallidum* infection [64]. In contrast to these findings, our study reveals an anti-inflammatory role of Glut1 during *S*. Typhimurium infection, where its inhibition in both macrophages and mice unexpectedly enhances inflammatory responses with increased cytokine expression and secretion. We propose that Glut1-driven glycolysis generates key immunometabolites that suppress innate immunity during infection. Specifically, elevated glucose levels may promote UDP-GlcNAc production via the hexosamine biosynthetic pathway (HBP), potentially contributing to immune suppression. Host O-GlcNAc transferase (OGT) utilizes UDP-GlcNAc to modify critical immune regulators like NF-κB subunits and RIPK3 through O-GlcNAcylation, thereby suppressing inflammatory signaling [65]. Concurrently, *S*. Typhimurium effector proteins (SseKs) exploit host UDP-GlcNAc to glycosylate death receptors (TNFR1 and Fas) via Arg-GlcNAcylation, further blocking immune activation [66, 67]. Additionally, infection-induced accumulation of pyruvate and lactate stimulates expression of the SPI-2 virulence system required for the secretion of effector proteins that impair host innate immunity [13]. In this regard, *S*. Typhimurium infection upregulates Glut1 to create an immunosuppressive microenvironment that favors bacterial survival and proliferation.

### Limitations of the study

While our study uncovers a three-pronged strategy by *S*. Typhimurium to exploit Glut1, the specific effector(s) mediating these processes remain unresolved. Due to functional redundancy among T3SS effectors, genetic dissection, such as using single or multiple effector deletion strains, will be essential to pinpoint the drivers of Glut1 induction, recruitment, and ubiquitination.

Additionally, the subcellular localization of Glut1 observed in this study is based on fixed imaging. While our data clearly show that Glut1 becomes associated with SCV/SIF during infection, fixed imaging cannot distinguish between active recruitment versus passive acquisition through ongoing endosomal trafficking. Given the highly dynamic nature of SCV/SIFs, live-cell imaging will be required in future studies to resolve Glut1 trafficking dynamics.

Moreover, while our data demonstrate that K29-linked ubiquitination enhances Glut1 transport activity on SCVs without altering its SCV association (Fig. 7F), we cannot rule out potential effects on Glut1 membrane dynamics or recycling. Future studies using FRAP analysis of GFP-Glut1 on SCVs or in vitro transport assays in proteoliposomes will be required to further distinguish between enhanced intrinsic activity and altered membrane retention.

Finally, while Glut1 inhibition enhanced anti-*S*. Typhimurium inflammatory responses in vitro and in vivo, the mechanistic links between Glut1-mediated glucose metabolism and immune activation remain unclear. Key pathways, such as mTOR-HIF-1α crosstalk or inflammasome signaling, may orchestrate this regulation but require deeper investigation.

## Supplementary Information

Supplementary Material 1.

Supplementary Material 2.

Supplementary Material 3.

## Data Availability

The raw transcriptome data reported in this paper have been deposited in the Genome Sequence Archive in BIG Data Center, Beijing Institute of Genomics (BIG) (https:/bigd.big.ac.cn/gsa) under the accession number CRA026604. The mass spectrometry proteomics data have been deposited in the ProteomeXchange Consortium (http:/proteomecentral.proteomexchange.org) via the iProX partner repository with the data set identifier PXD064768. The metabolomic data have been deposited in MetaboLights (https:/www.ebi.ac.uk/metabolights) under the accession number MTBLS12589. Full uncropped blot images supporting these findings have been deposited in Zenodo (https://doi.org/10.5281/zenodo.17646548). Other data supporting the findings of this study and source data are included in the supplementary data and available upon reasonable request.

## Abbreviations

AP-1: Activator Protein 1
BA-GLUT1: Bacteria-Associated GLUT1
BFA: Brefeldin A
CFU: Colony Forming Unit
CHX: Cycloheximide
DEG: Differentially Expressed Gene
ECAR: Extracellular Acidification Rate
EPEC: *Enteropathogenic Escherichia coli*
FPKM: Fragments Per Kilobase of transcript per Million mapped reads
GO: Gene Ontology
HBP: Hexosamine Biosynthetic Pathway
HCD: Higher-Energy Collisional Dissociation
IAM: Infection-Associated Macropinosome
IHC: Immunohistochemistry
IRS: Immunoreactive Score
KEGG: Kyoto Encyclopedia of Genes and Genomes
LC–MS/MS: Liquid Chromatography with Tandem Mass Spectrometry
MAPK: Mitogen-Activated Protein Kinase
MOI: Multiplicity of Infection
ORF: Open Reading Frame
RISC: RNA-Induced Silencing Complex
SCV: *Salmonella*-Containing Vacuole
SIF: *Salmonella*-Induced Filament
SNARE: Soluble N-ethylmaleimide-sensitive Factor Attachment Protein Receptor
SPI: *Salmonella* Pathogenicity Island
T3SS: Type III Secretion System
Ub: Ubiquitin
v-ATPase: Vacuolar-type ATPase
2DG6P: 2-Deoxyglucose-6-Phosphate
3PG: 3-Phosphoglycerate
