# Supplementary material for: Salmonella Typhimurium hijacks a host glucose transporter for intravacuolar proliferation
Source: Cell Commun Signal. 2026 Jun 5;24:438. doi: 10.1186/s12964-026-02981-2 (PMC13455383; doi:10.1186/s12964-026-02981-2)
Supplement: Supplementary file 1 [file 12964_2026_2981_MOESM1_ESM.pdf]

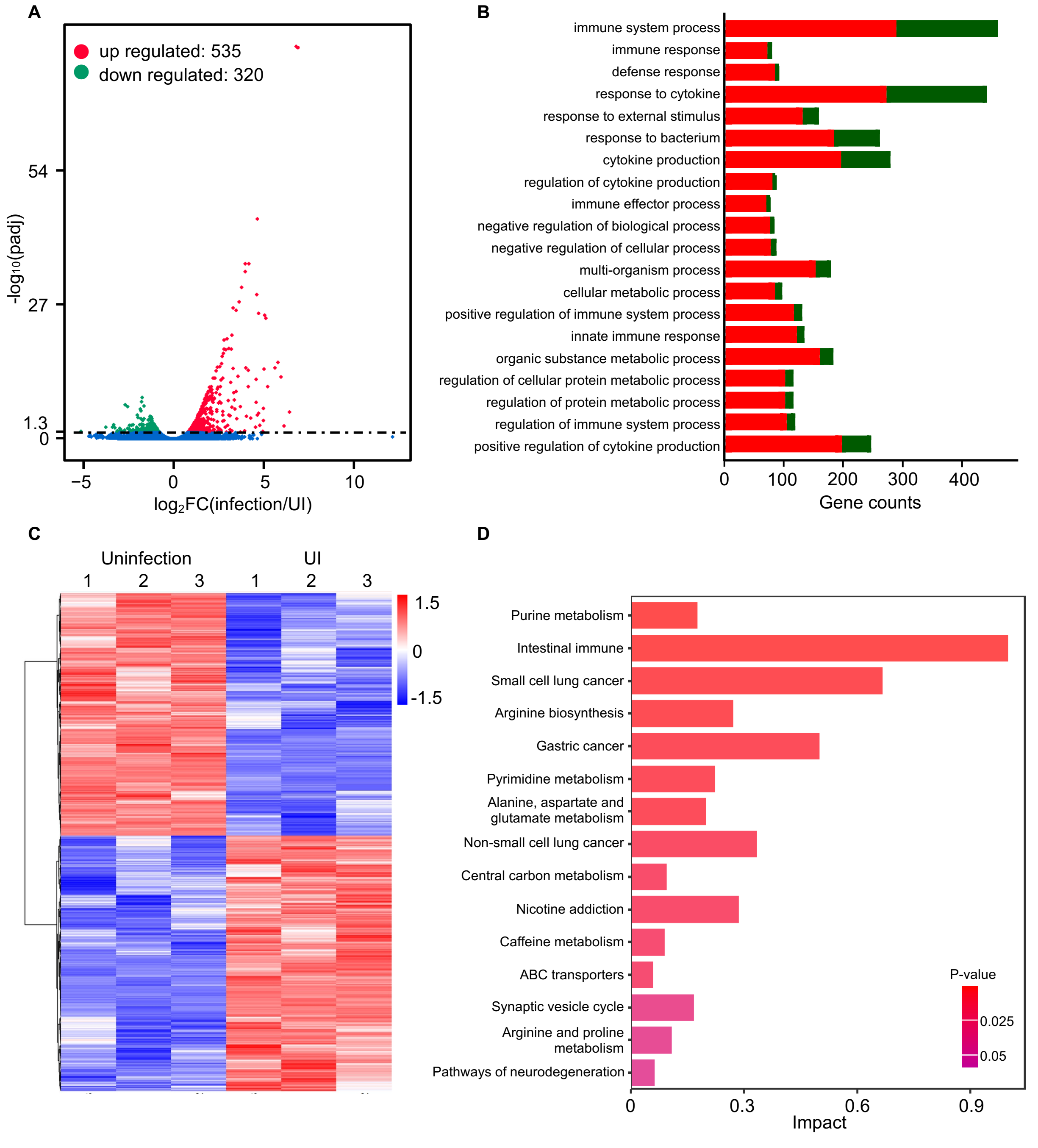


**Supplementary Figure 1. Integrated transcriptomic and metabolomic analysis of RAW264.7 macrophages at 10 hours post-infection with *S.* Typhimurium.**

**(A) Genome-wide transcriptomic alterations induced by *S.* Typhimurium infection.** Volcano plot displays DEGs between infected and uninfected macrophages. Significantly upregulated genes (FDR < 0.05, $\log_2FC$ (fold change) > 1) are shown in red; significantly downregulated genes (FDR < 0.05, $\log_2FC < -1$) are shown in green. Genes without significant expression changes are shown in blue.

**(B) Functional enrichment analysis of transcriptional responses.** GO pathway analysis reveals biological pathways significantly enriched among DEGs (FDR < 0.05). Red bars represent pathways enriched with upregulated genes, while green bars denote pathways enriched with downregulated genes. Bar length corresponds to the number of DEGs mapped to each pathway.

**(C) Metabolomic reprogramming in infected macrophages.** Hierarchical clustering analysis of significantly altered metabolites ($p < 0.05$) shows clear separation between infected and control groups. Metabolite abundance values are z-score normalized across samples, with red indicating higher expression and blue indicating lower expression relative to the mean.

**(D) Metabolic pathway impact analysis.** KEGG pathway enrichment results for differential metabolites ($p < 0.05$). Bar height represents the pathway impact score derived from topological analysis, indicating the relative importance of each pathway in the metabolic perturbation induced by infection.

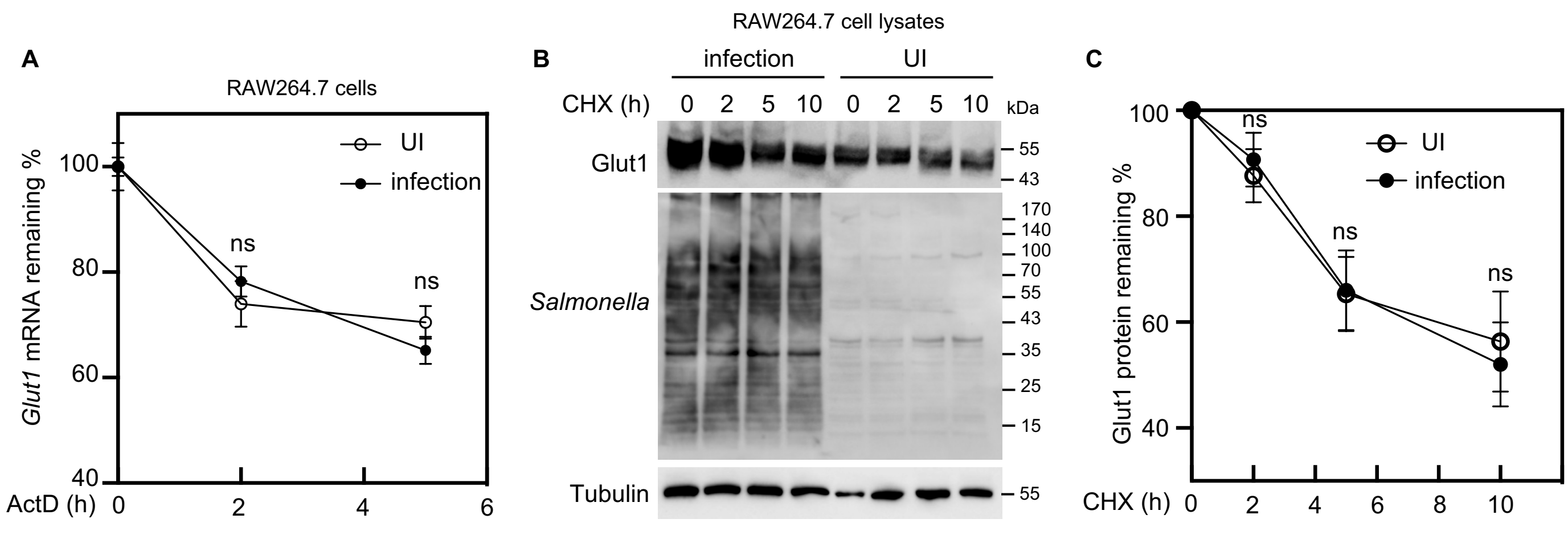


**Supplementary Figure 2. *S.* Typhimurium infection does not affect the Glut1 stability at transcriptional and translational levels in RAW264.7 cells.**

**(A)** The stability of *Glut1* mRNA probed by the ActD chase assay. RAW264.7 cells were infected with *S*. Typhimurium for 10 h or mock-infected and were treated with ActD for the indicated time points. The mRNA levels were quantified by qPCR and expressed relative to the 0 h time point within each group.

**(B-C)** The stability of Glut1 protein was probed by the CHX pulse chase assay. RAW264.7 cells were infected with *S*. Typhimurium strains for 10 h and were treated with CHX at the indicated time points. **(B)** Total lysates were isolated and immunoblotted with the corresponding antibodies. **(C)** The band intensity of Glut1 was quantified using ImageJ software, normalized to tubulin, and expressed relative to the 0 h time point within each group. The curve represents a non-linear regression of protein decay. Results shown are mean values ± SD from three independent experiments. Statistical significance was determined by Student's *t*-test. ns, not significant.

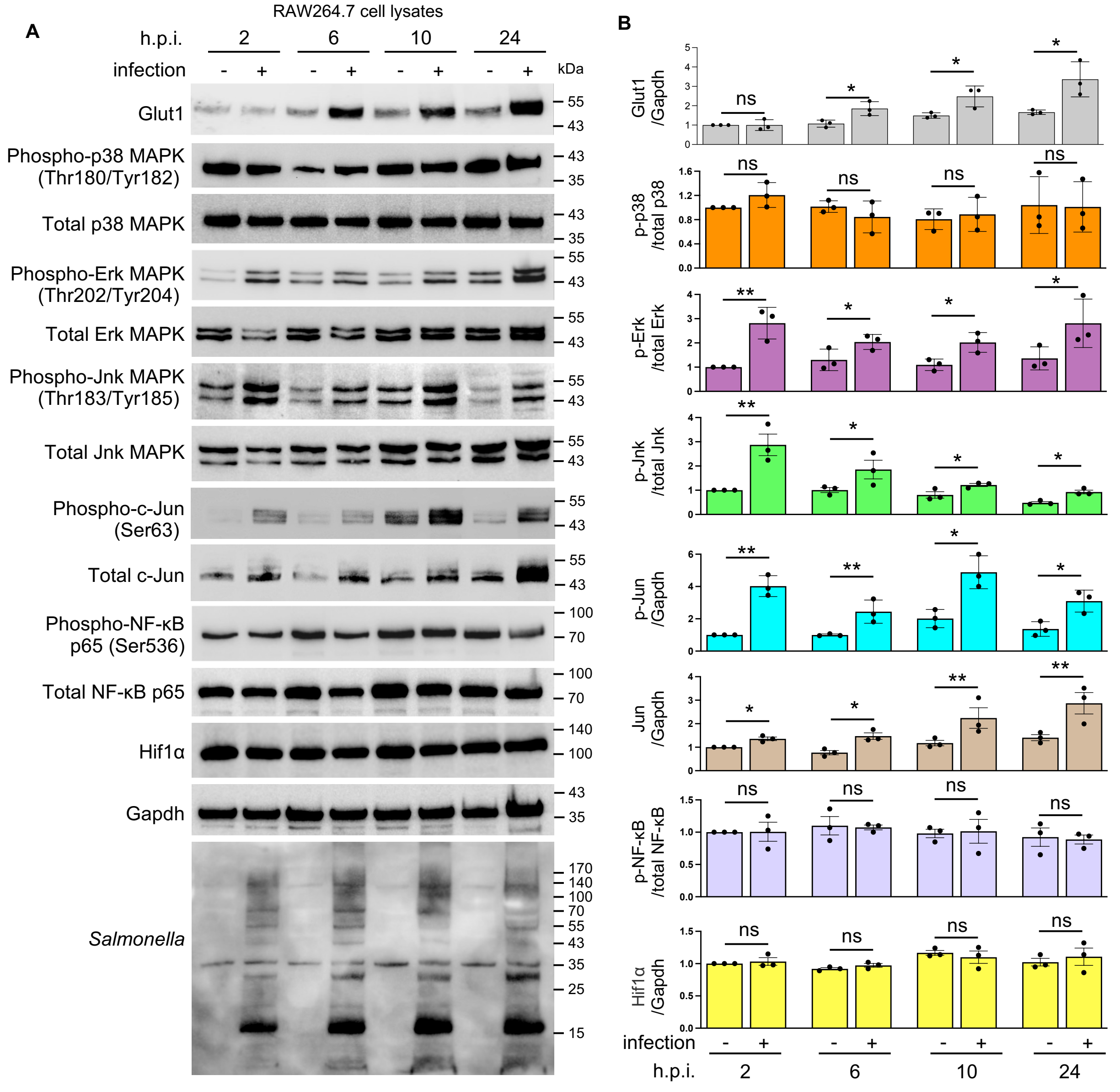


**Supplementary Figure 3. The effects of *S.* Typhimurium infection on MAPK pathway activation and transcription factor expression in RAW264.7 cells.**

**(A) Time-course immunoblot analysis of MAPK signaling activation and transcription factor expression.** Phosphorylated and total levels of Erk1/2, Jnk, p38, c-Jun, NF-κB p65, and HIF1α were assessed in RAW264.7 cells from 2 to 24 hours post-infection with *S.* Typhimurium. Gapdh served as loading control, and *Salmonella*-specific antibody confirmed infection. Blots shown are representative of three independent experiments.

**(B) Quantitative analysis of signaling molecule activation.** Phosphorylated NF-κB and MAPK protein levels were normalized to their corresponding total proteins, while c-Jun and HIF1α levels were normalized to Gapdh. Data are expressed as fold change relative to the 2-hour uninfected control (set as 1). All values represent mean ± SD from three independent experiments. *$p < 0.05$, **$p < 0.01$ (Student's *t*-test). This panel corresponds to the immunoblots shown in Figure 1F and Figure 2B.

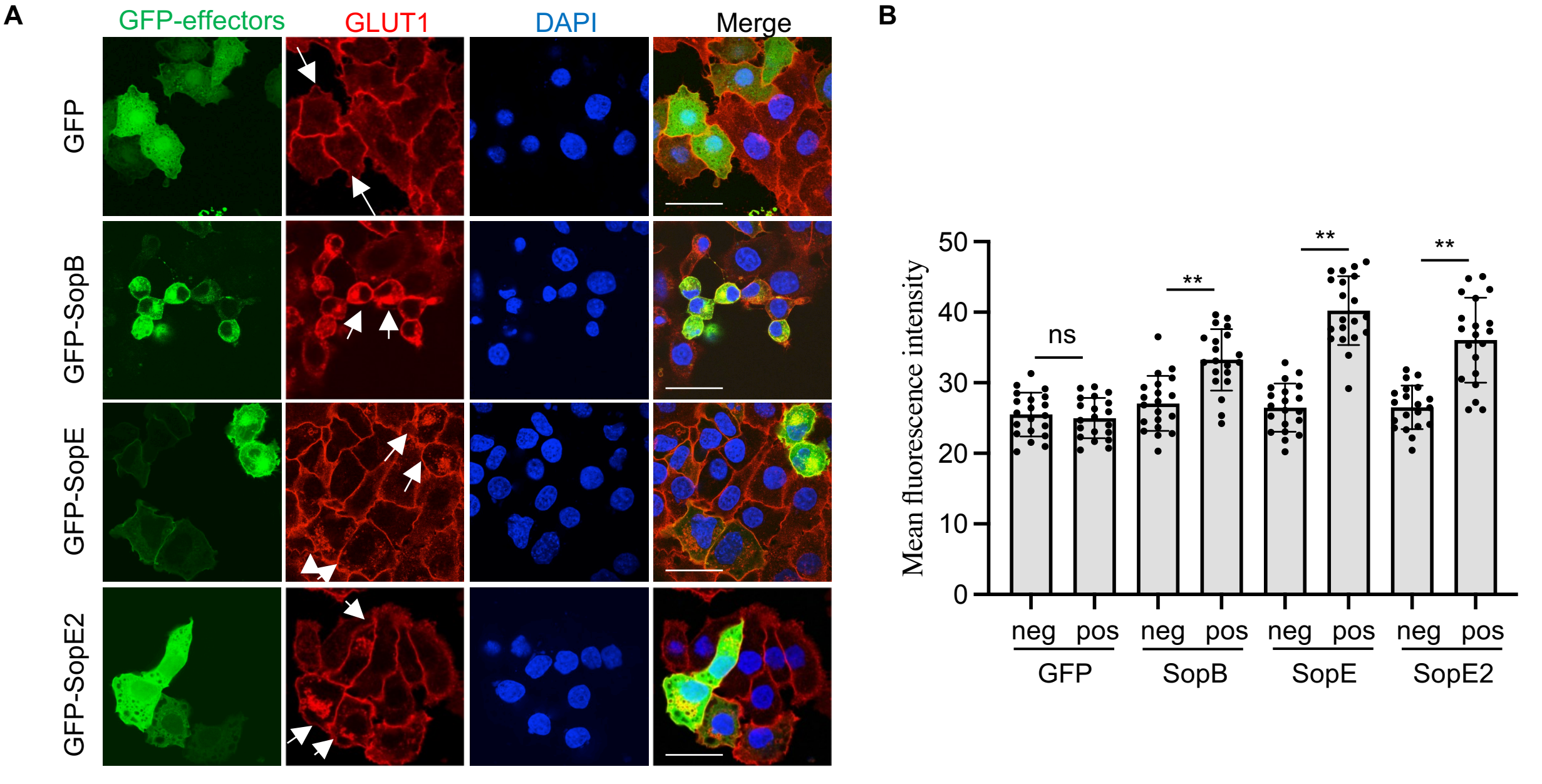


**Supplementary Figure 4. Effects of SopB, SopE, or SopE2 transfection on endogenous GLUT1 expression in HeLa epithelial cells by immunofluorescence.** HeLa cells were transfected with GFP-tagged expression constructs encoding SopB, SopE, or SopE2 (green), or with GFP alone as a control. Endogenous GLUT1 was visualized by immunofluorescence (red), and nuclei were counterstained with DAPI (blue).

**(A)** Representative immunofluorescence images. Arrows indicate representative transfected-positive cells. Scale bar, 50 µm.

**(B)** Quantification of GLUT1 mean fluorescence intensity (MFI) after normalizing to cell area from at least 20 cells per condition. Data are presented as mean ± SD; $**p < 0.01$ compared to the transfected-negative control.

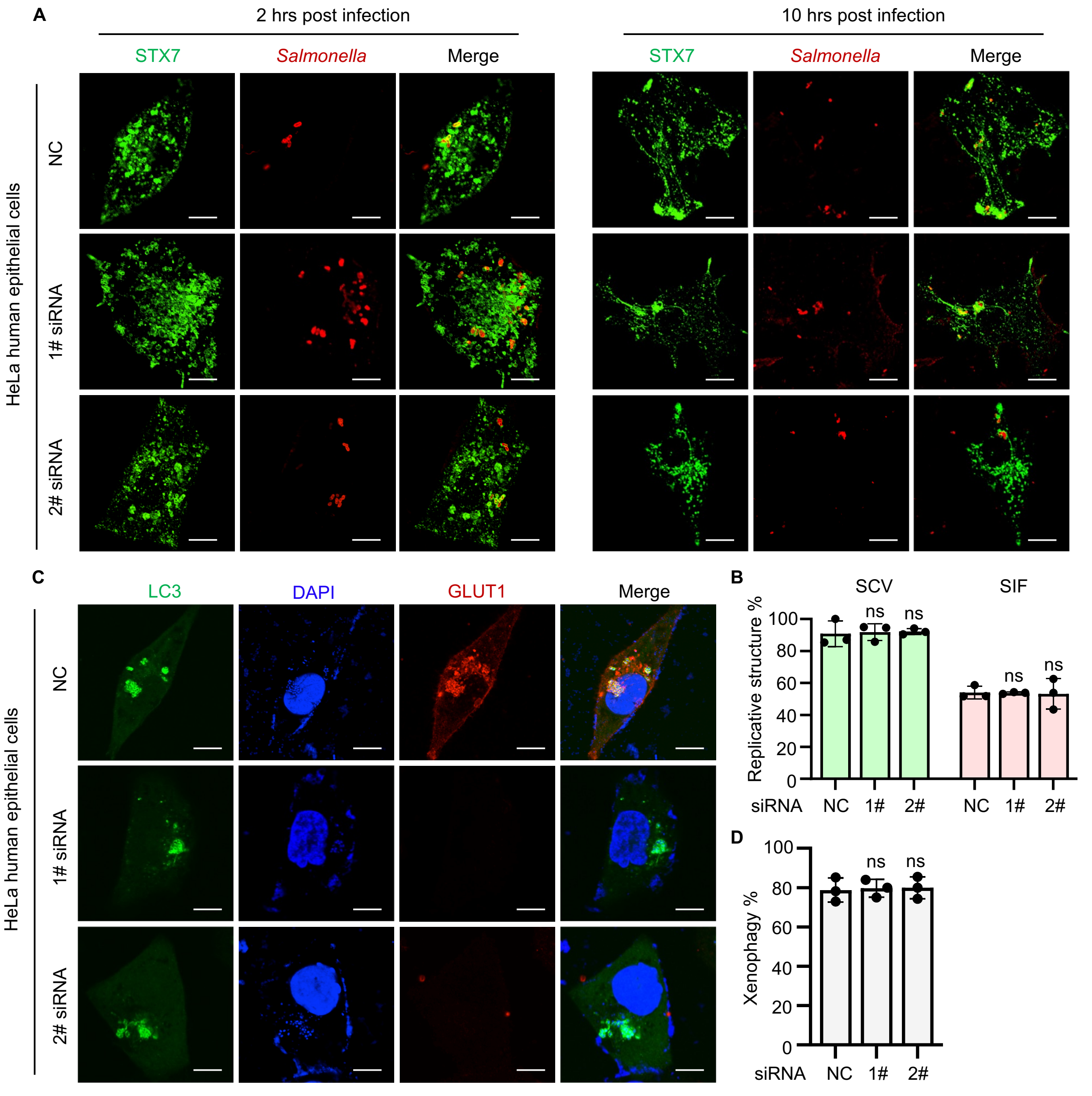


**Supplementary Figure 5. Effects of GLUT1 knockdown on *S.* Typhimurium replicative structures and xenophagy in HeLa cells.**

**(A-B)** Effects of GLUT1 knockdown on the *S.* Typhimurium replicative structures. STX7 stably expressing cells were transfected with two distinct GLUT1-targeting siRNA pairs for 48 h, followed by *S.* Typhimurium infection for 2 h and 10 h, and analyzed for SCVs and SIFs. (**A**) Representative images showing the distribution of STX7 (green) and *S.* Typhimurium (red). Scale bar, 10 µm. (**B**) Quantification of STX7-positive bacteria (SCVs) and tubules (SIFs). At least 50 cells were counted for samples from experiments done in triplicate.

**(C-D)** Effects of GLUT1 knockdown on xenophagy. LC3 stably expressing cells were transfected with two distinct GLUT-targeting siRNA pairs for 48 h, followed by *S.* Typhimurium Δ*sopF* mutant for 1 h. **(C)** Representative images showing the distribution of LC3 (green), endogenous GLUT1 (red), and nucleus (blue). Scale bar, 10 µm. **(D)** Quantification of LC3-bacteria colocalization, representing xenophagic activity. Data were obtained from ≥100 cells per condition across three independent experiments. Values represent mean ± SD. Statistical significance was determined by Student's *t*-test compared to the NC group. ns, not significant.

**A**

**Flag-GLUT1 (63% Coverage)**

MDYKDHGDYKDHDIDYKDDDDKLAAANSGRPMEPSSKKLTGRLMLAVGGAVLGSLQFGYNTGVINAPQKVIEEFYNQ
TWVHRYGESILPTTLTTLWSLSVAIFSVGGMIGSFSVGLFVNRFGRRNSMLMMNLLAFVSAVLMGFSKLGK**SFEMLILG
R**FIIGVYCGLTTGFVPMYVGEVSPTALRGALGTLHQLGIVVGILIAQVFGLDSIMGNKDLWPLLLSIIFIPALLQCIVLPFCP
ESPRFLLINRNEENRAKSVLKKLR**GTADVTHDLQEMKEESR**QMMR**EKKVTILELFR**SPAYRQPILIAVVLQLSQQLSGI
NAVFYYSTSIFEKAGVQQPVYATIGSGIVNTAFTVVSLFVVERAGRRTLHLIGLAGMAGCAILMTIALALLEQLPWMSYLS
IVAIFGFVAFFEVGPGPIPWFIVAELFSQGPRPAAIAVAGFSNWTSNFIVGMCFQYVEQLCGPYVFIIFTVLLVLFFIFTYFKVP
ETKGRTFDEIASGFR**QGGASQSDKTPEELFHPLGADSQV**

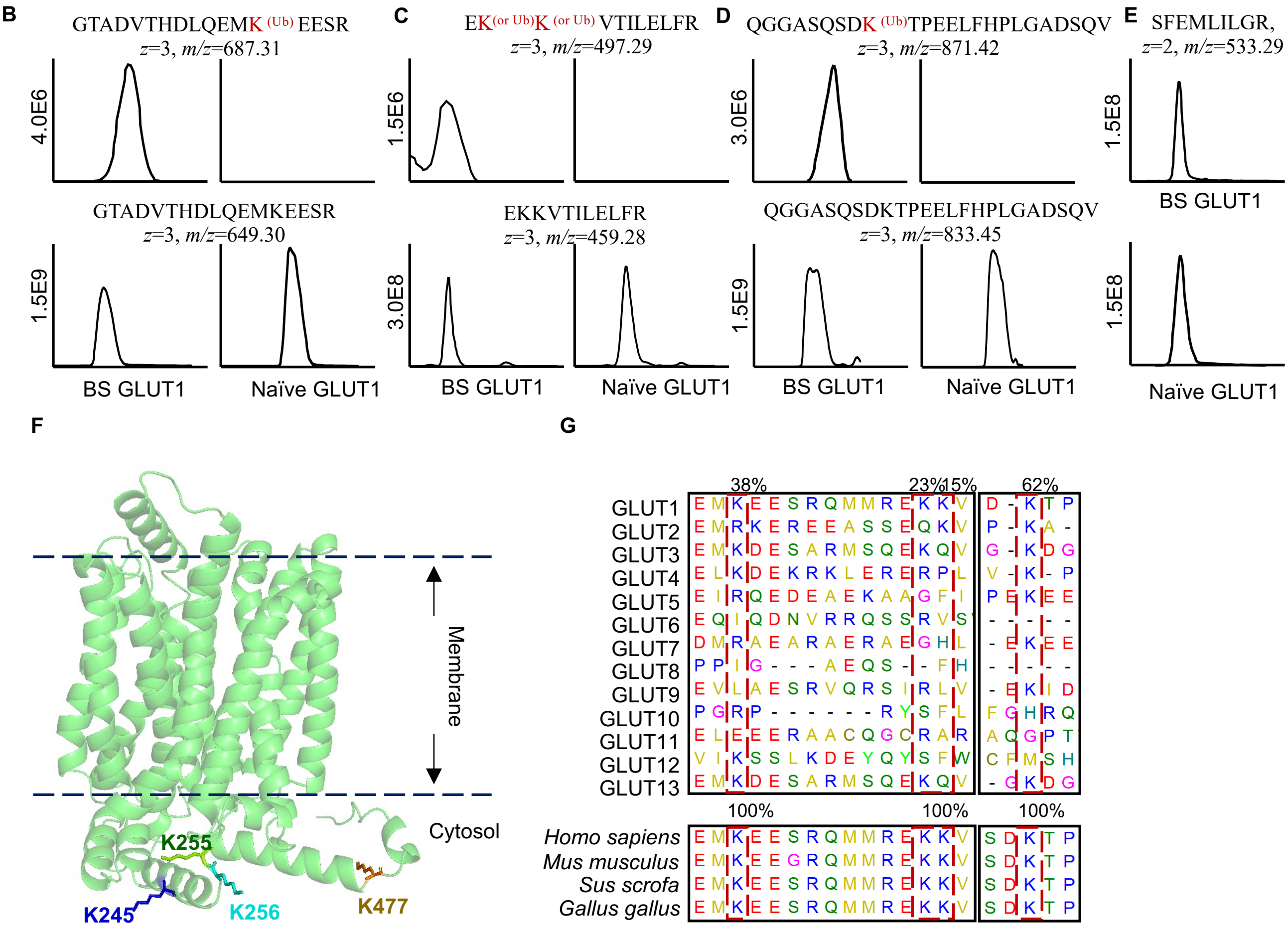


**Supplementary Figure 6. MS detection and sequence analysis of GLUT1.**

**(A-E)** MS detection of GLUT1 peptide intensities. Immunoprecipitated Flag-GLUT1 (naïve GLUT1 and BA-GLUT1) in Figure 5A was digested with trypsin and analyzed by LC-MS/MS. **(A)** The detected GLUT1 sequence is shown in grey in LC-MS experiments. The ubiquitinated peptide sequences detected in BA-GLUT1 are underlined, and the modification sites are in green. The peptides $^{232-}$GTADVTHDLQEMKEESR$^{-249}$ **(B)**, $^{253-}$EKKVTILELFR$^{-264}$**(C)**, and $^{468-}$QGGASQSDKTPEELFHPLGADSQV$^{-492}$ **(D)** covalently modified with the Ub molecule are shown, and the modified lysines are labeled in red. The control peptides are displayed in **(E)**. These data support the ubiquitination sites identified in Figure 6C.

**(F)** Visualization of these ubiquitination sites (shown as stick models) in the GLUT1 structure (PDB: 4PYP).

**(G)** Evolutionary conservation analysis of K245/255/256/477 sites. Top: Multiple sequence alignment of the K245/255/256/477-containing region across human GLUT family members (GLUT1-13). Bottom: Cross-species conservation of these lysine residues in GLUT1 orthologs from mammalian hosts of *Salmonella*.

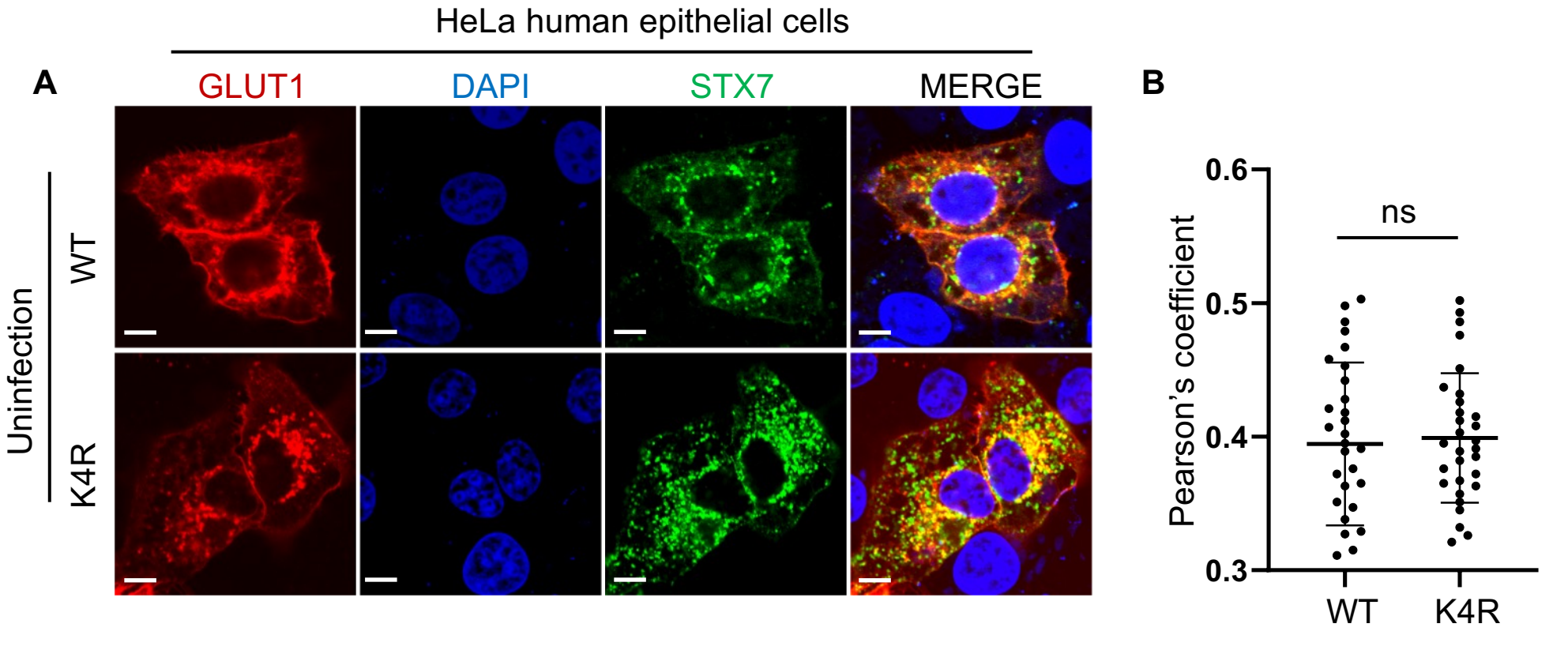


**Supplementary Figure 7. Subcellular distribution of GLUT1 K4R mutant under uninfection conditions.** STX7-stably expressing HeLa cells were transfected with RFP-tagged WT GLUT1 or K4R mutant. **(A)** Confocal microscopy analysis. Representative images showing STX7 (green), RFP-GLUT1 (red), and nuclei (blue). Scale bar: 10 µm. **(B)** Quantification of STX7-GLUT1 localization. At least 30 cells were counted for samples from experiments done in triplicate. Values represent mean ± SD. Statistical significance was determined by Student's *t*-test compared to the WT group. ns, not significant.

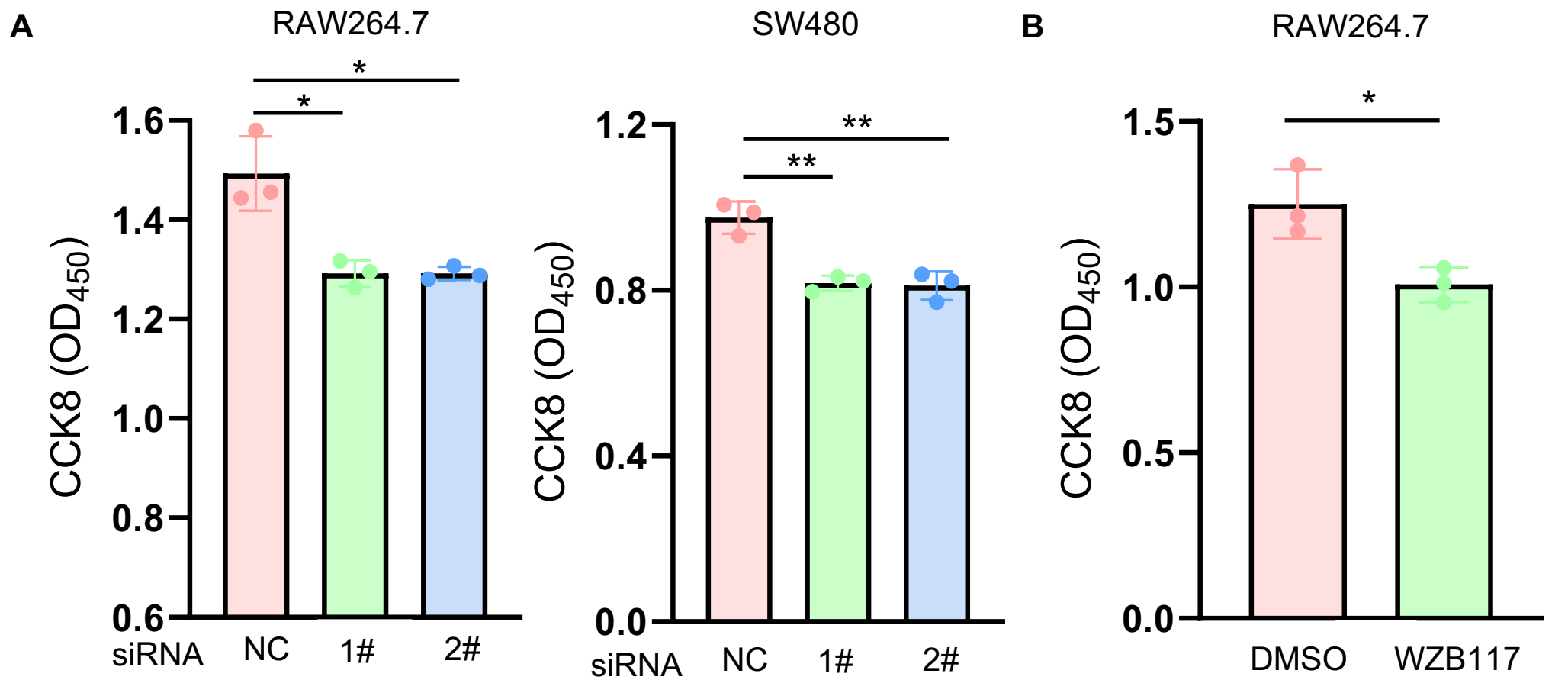


**Supplementary Figure 8. Effects of Glut1 inhibition on host cell viability.**
(A-B) Cell proliferation was assessed using the CCK-8 assay following Glut1 knockdown by siRNA (A) or treatment with the Glut1 inhibitor WZB117 (B). Data are presented as mean ± SD from three independent experiments (*$p < 0.05$, **$p < 0.01$).
