# Supplementary material for: Salmonella Typhimurium hijacks a host glucose transporter for intravacuolar proliferation
Source: Cell Commun Signal. 2026 Jun 5;24:438. doi: 10.1186/s12964-026-02981-2 (PMC13455383; doi:10.1186/s12964-026-02981-2)

Figure 1

Figure 1F

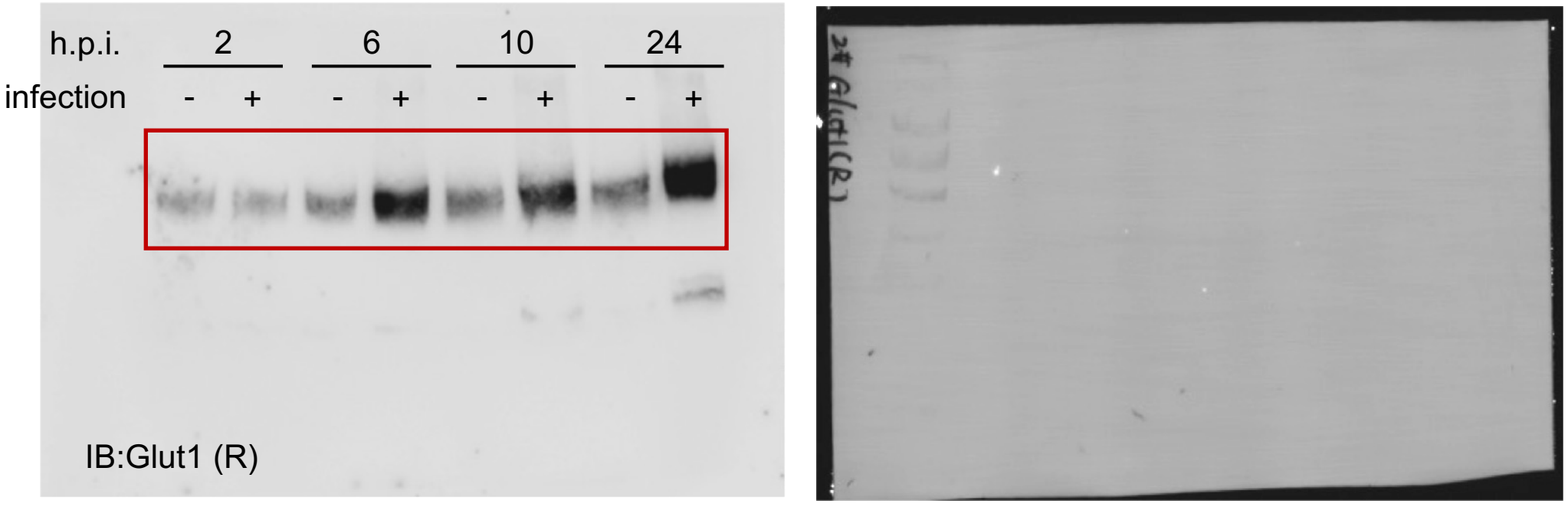

Figure 1G (left)

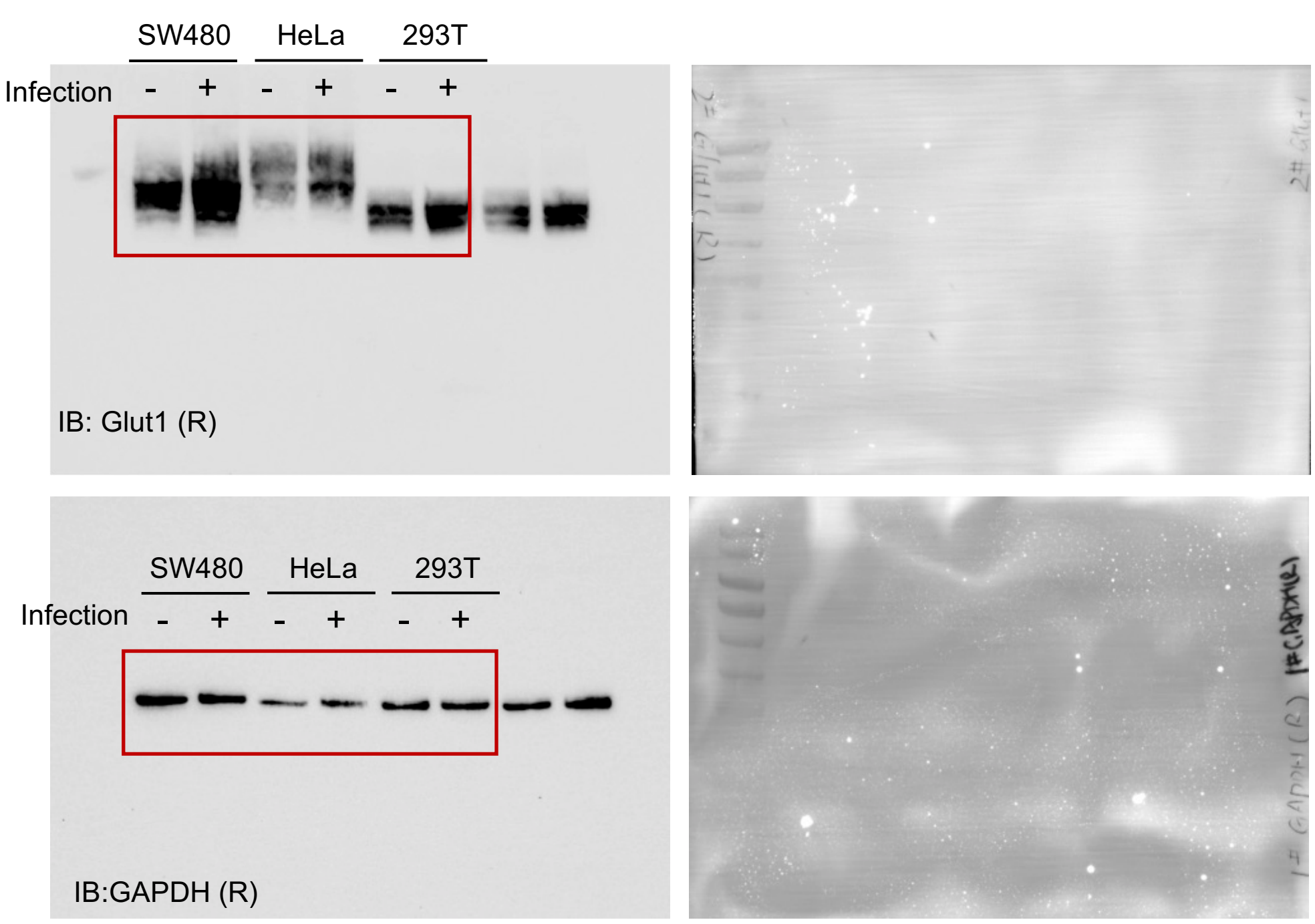

Figure 1G (right)

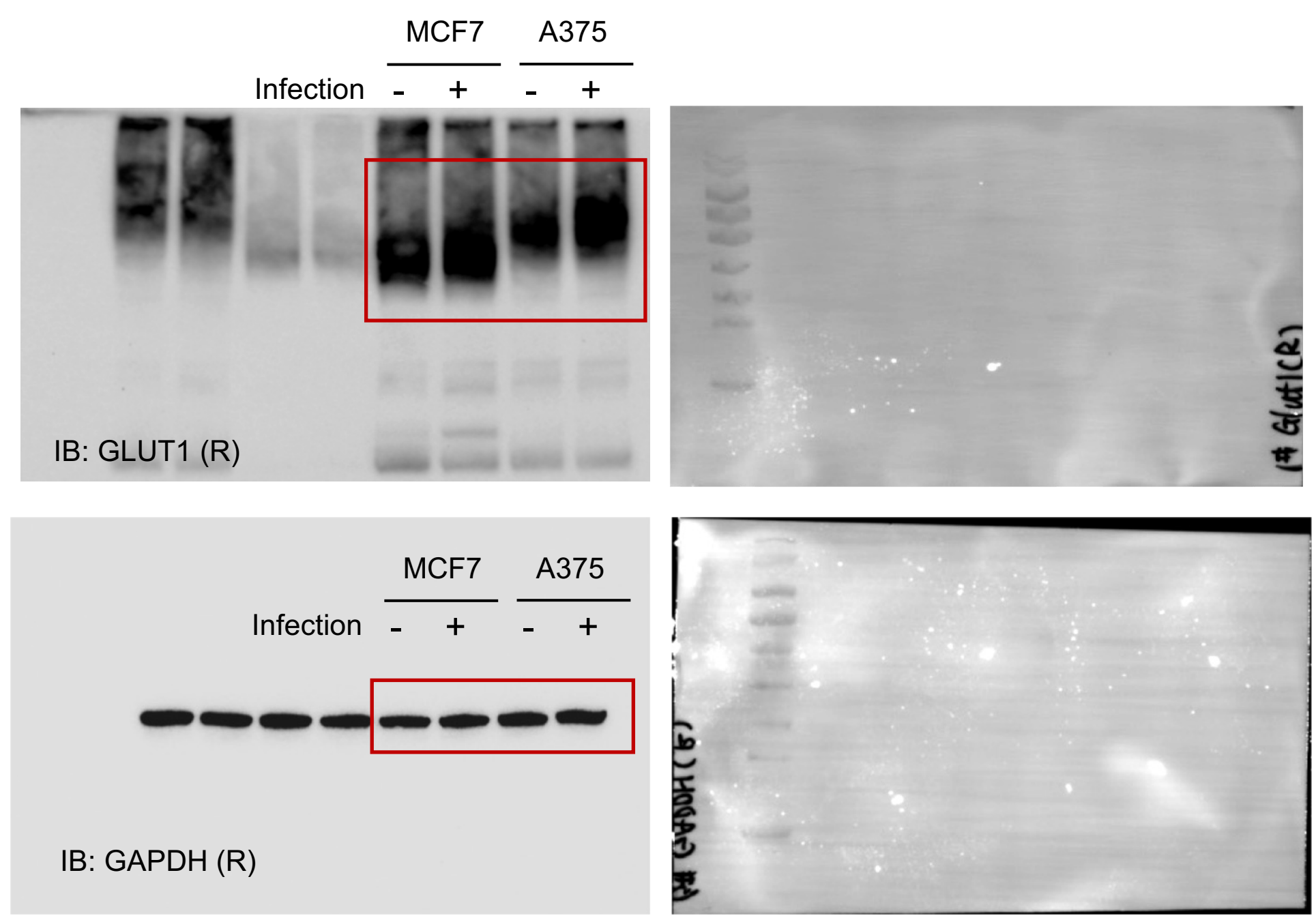

Figure 2B and Supplementary Figure 3A (1)

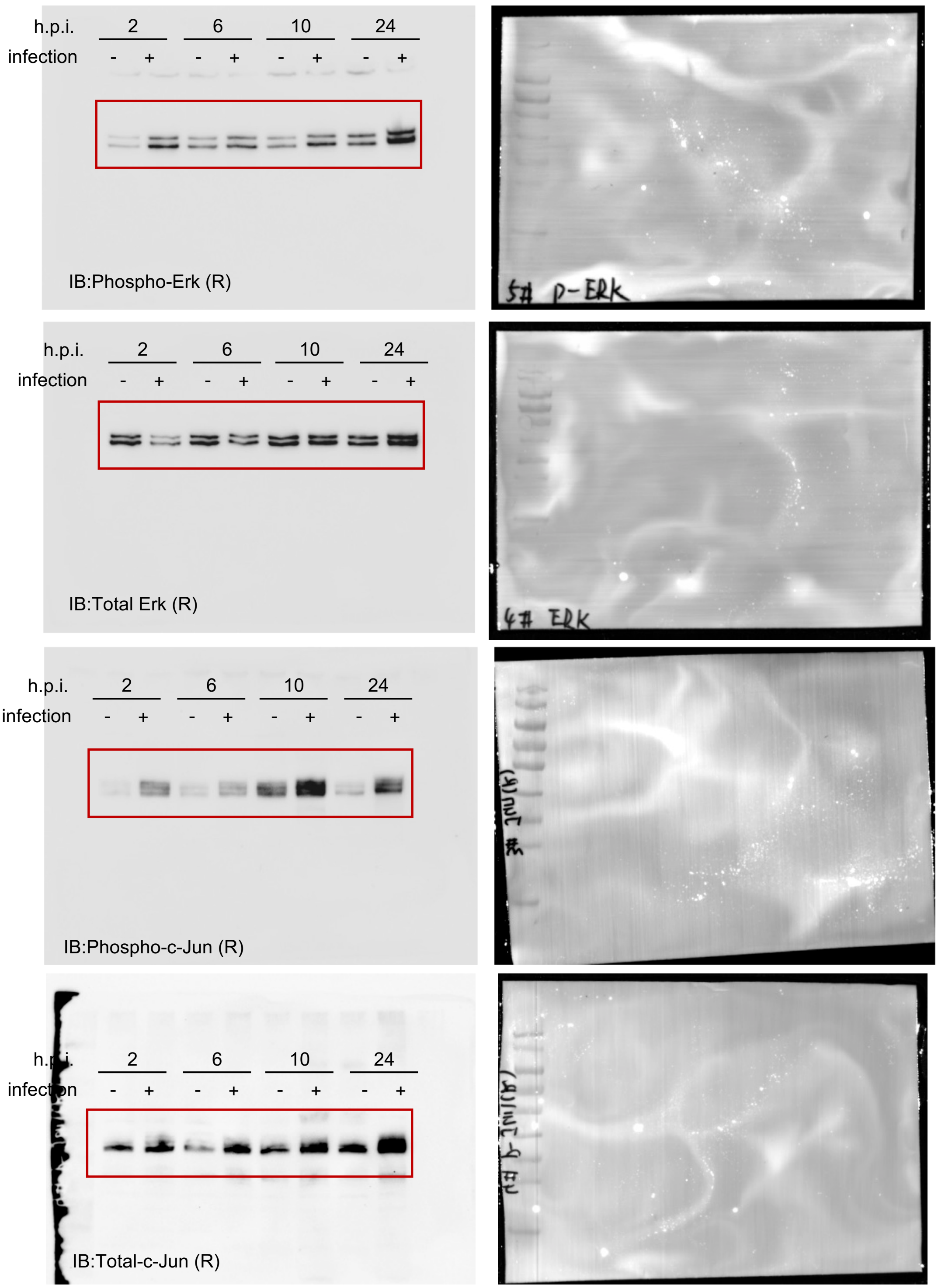

Figure 2B and Supplementary Figure 3A (2)

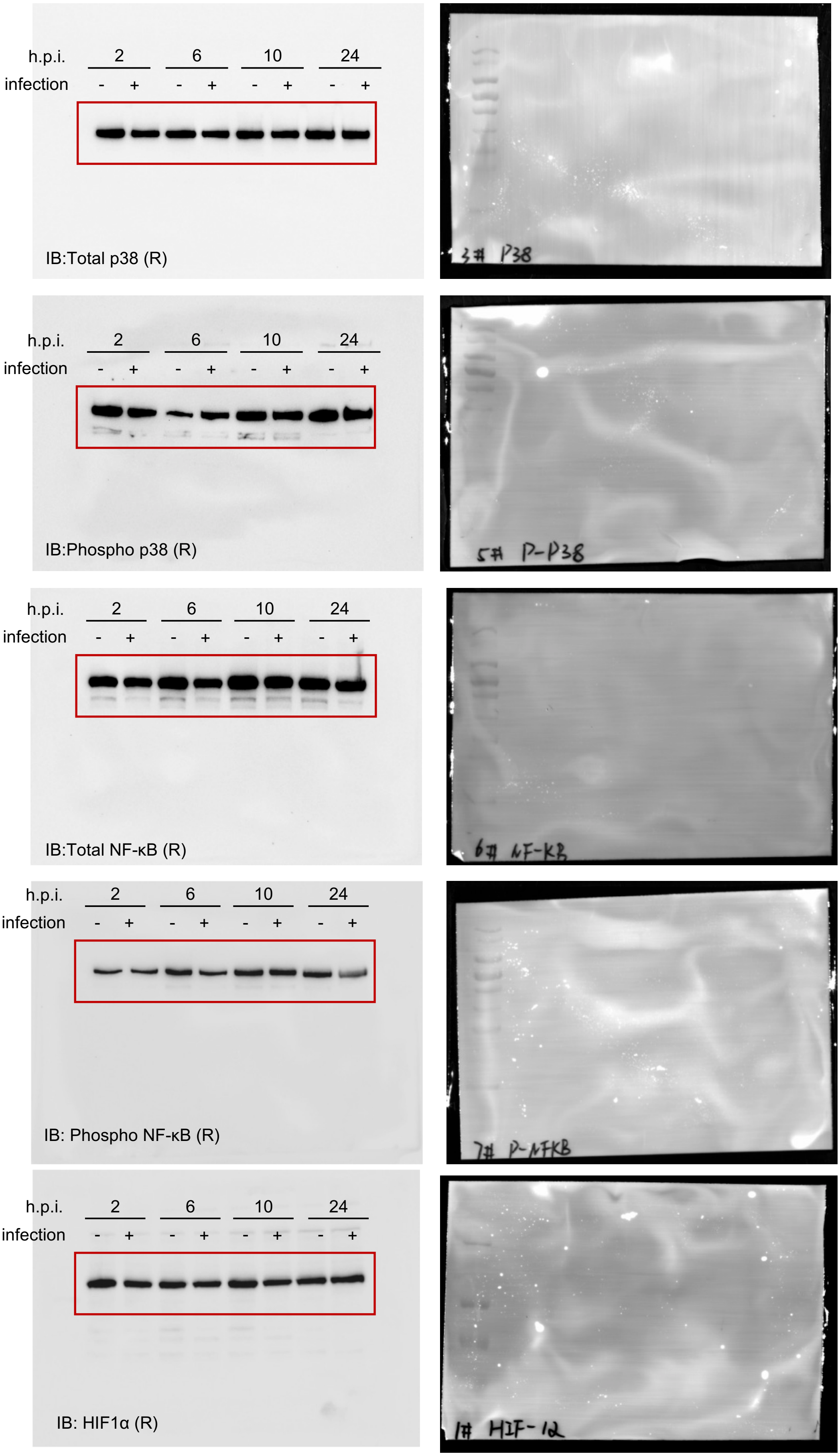

Figure 2B and Supplementary Figure 3A (3)

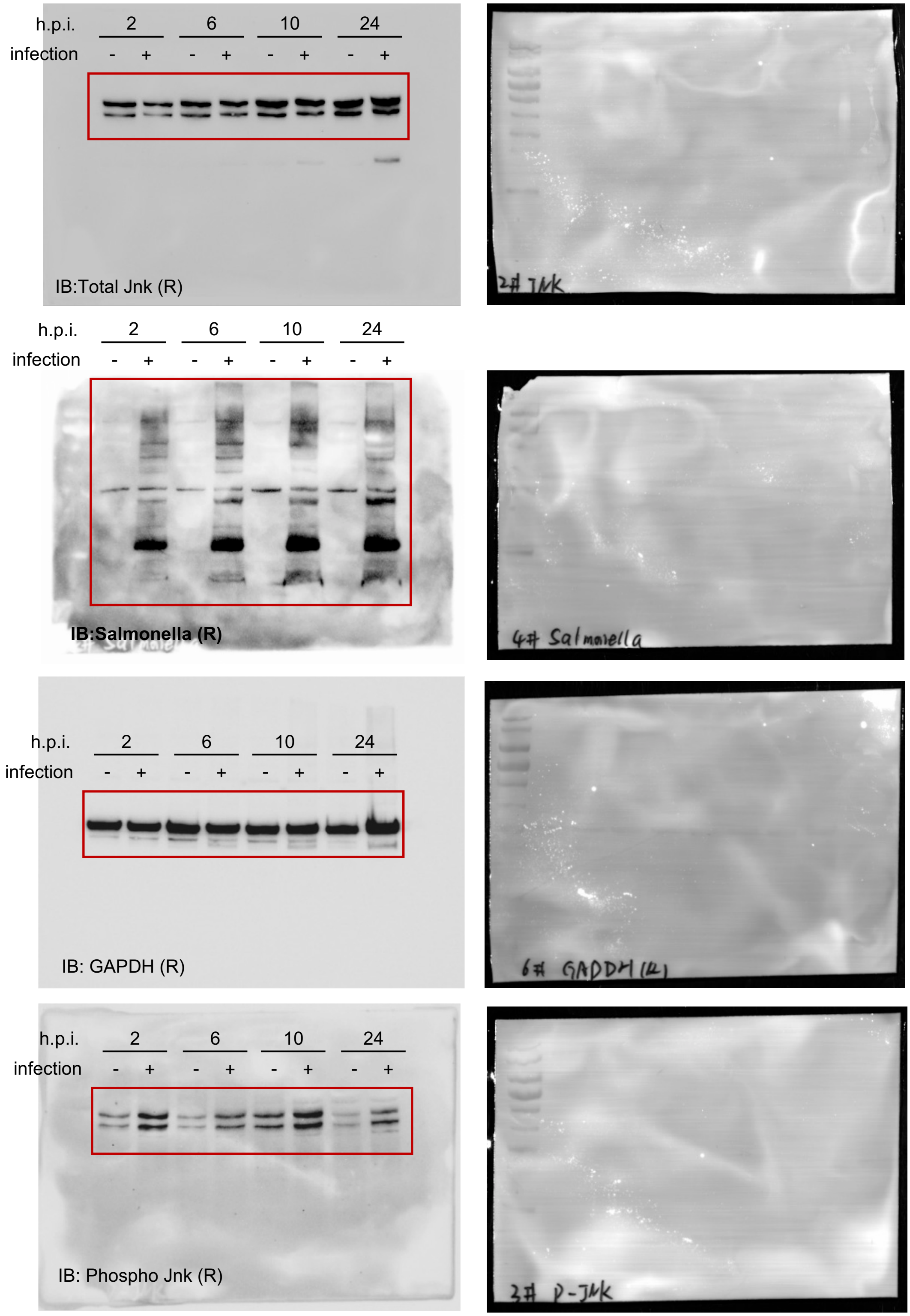

Figure 2D

Figure 2D (left)

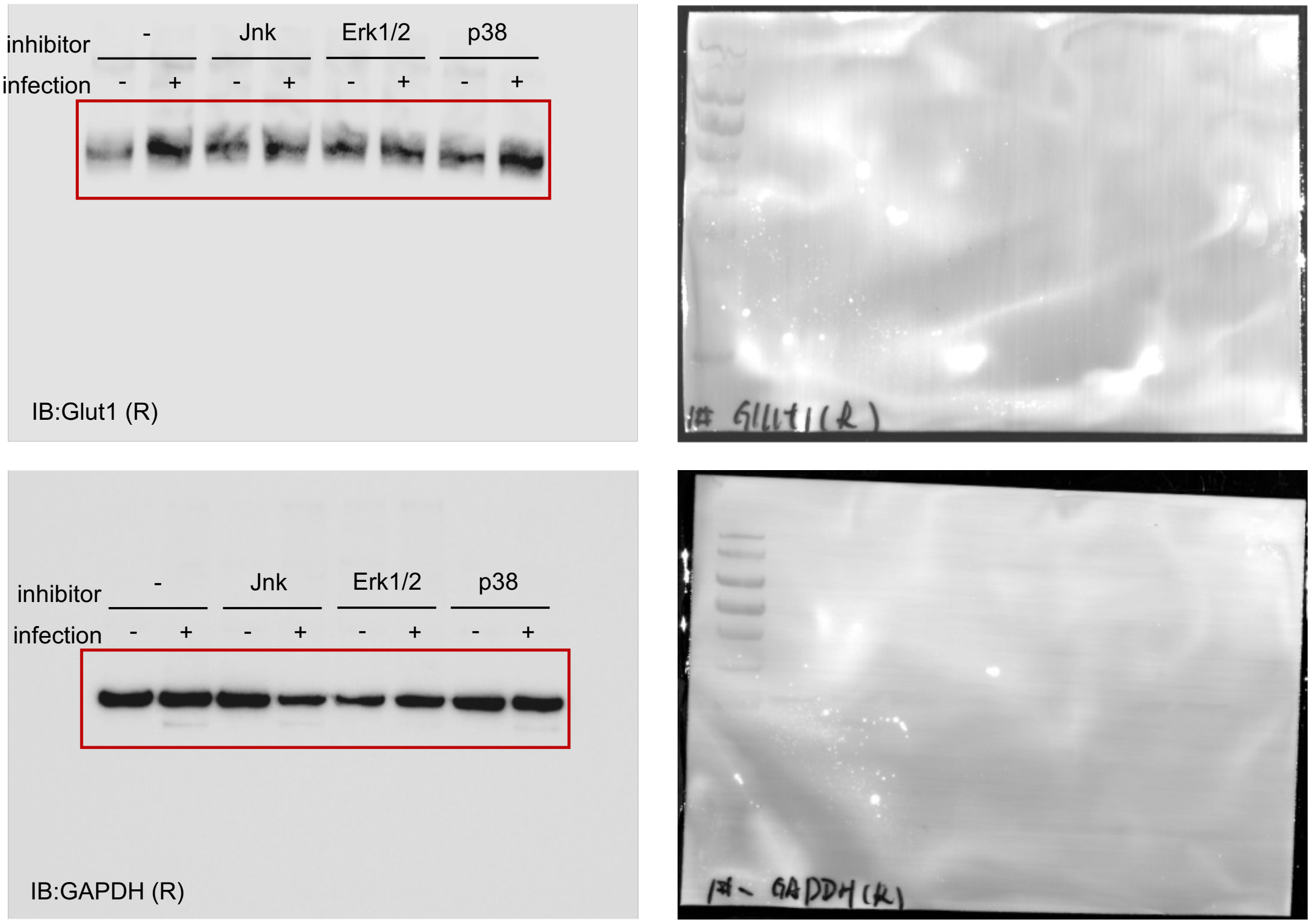

Figure 2D (right)

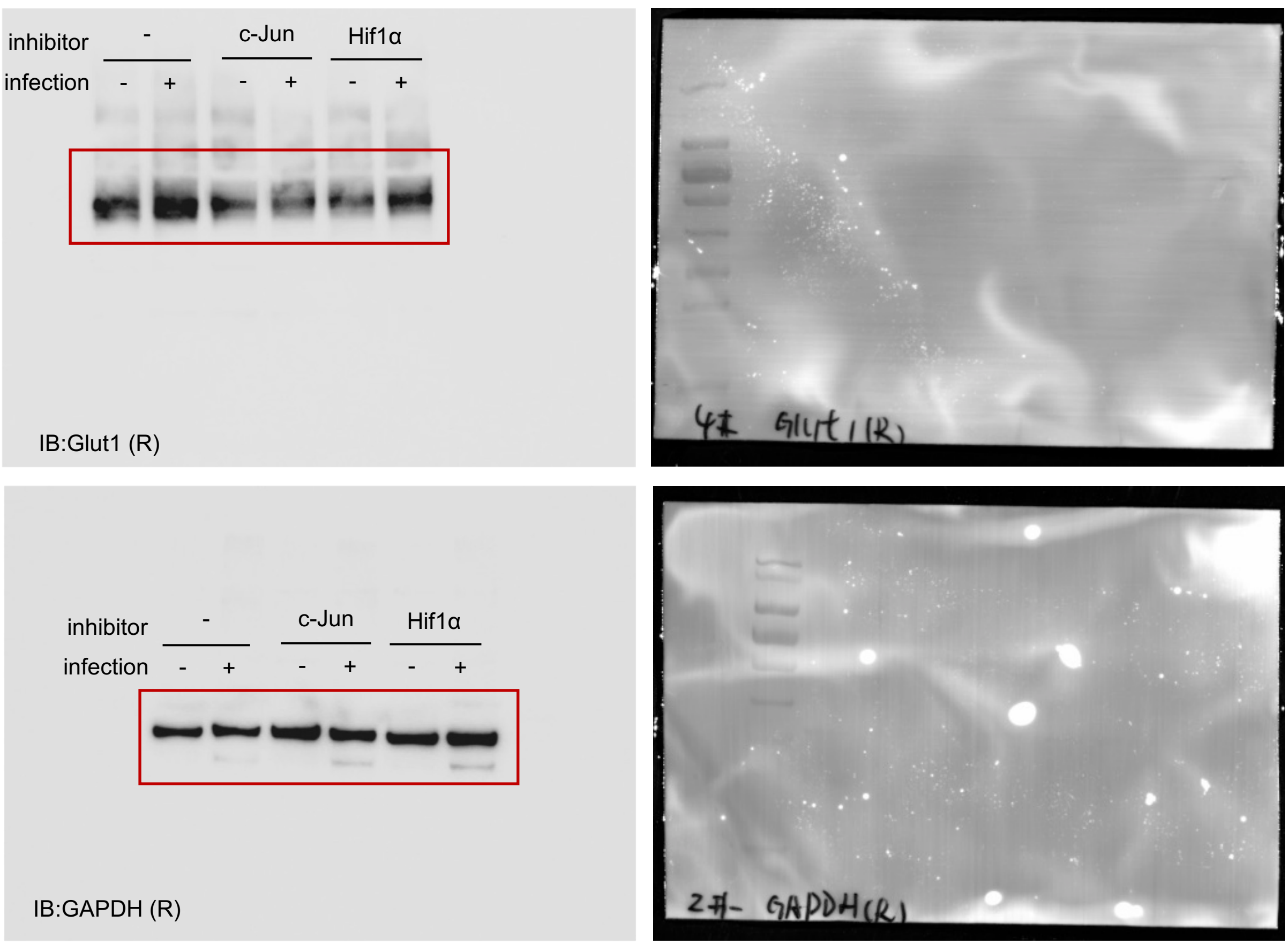

Figure 2H

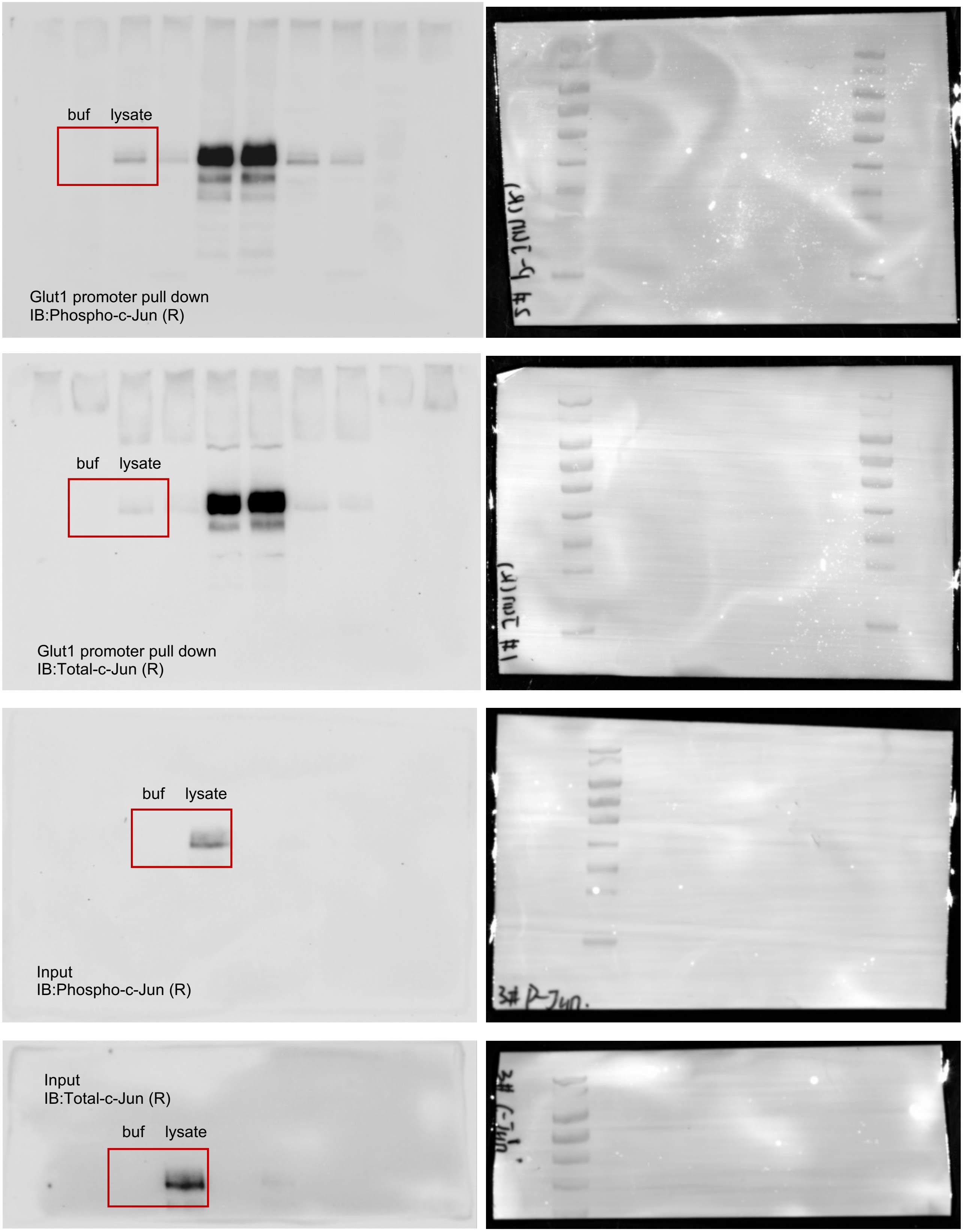
buf
lysate
Glut1 promoter pull down
IB:Phospho-c-Jun (R)
buf
lysate
Glut1 promoter pull down
IB:Total-c-Jun (R)
buf
lysate
Input
IB:Phospho-c-Jun (R)
Input
IB:Total-c-Jun (R)
buf
lysate

Figure 3

Figure 3B

Figure 3A

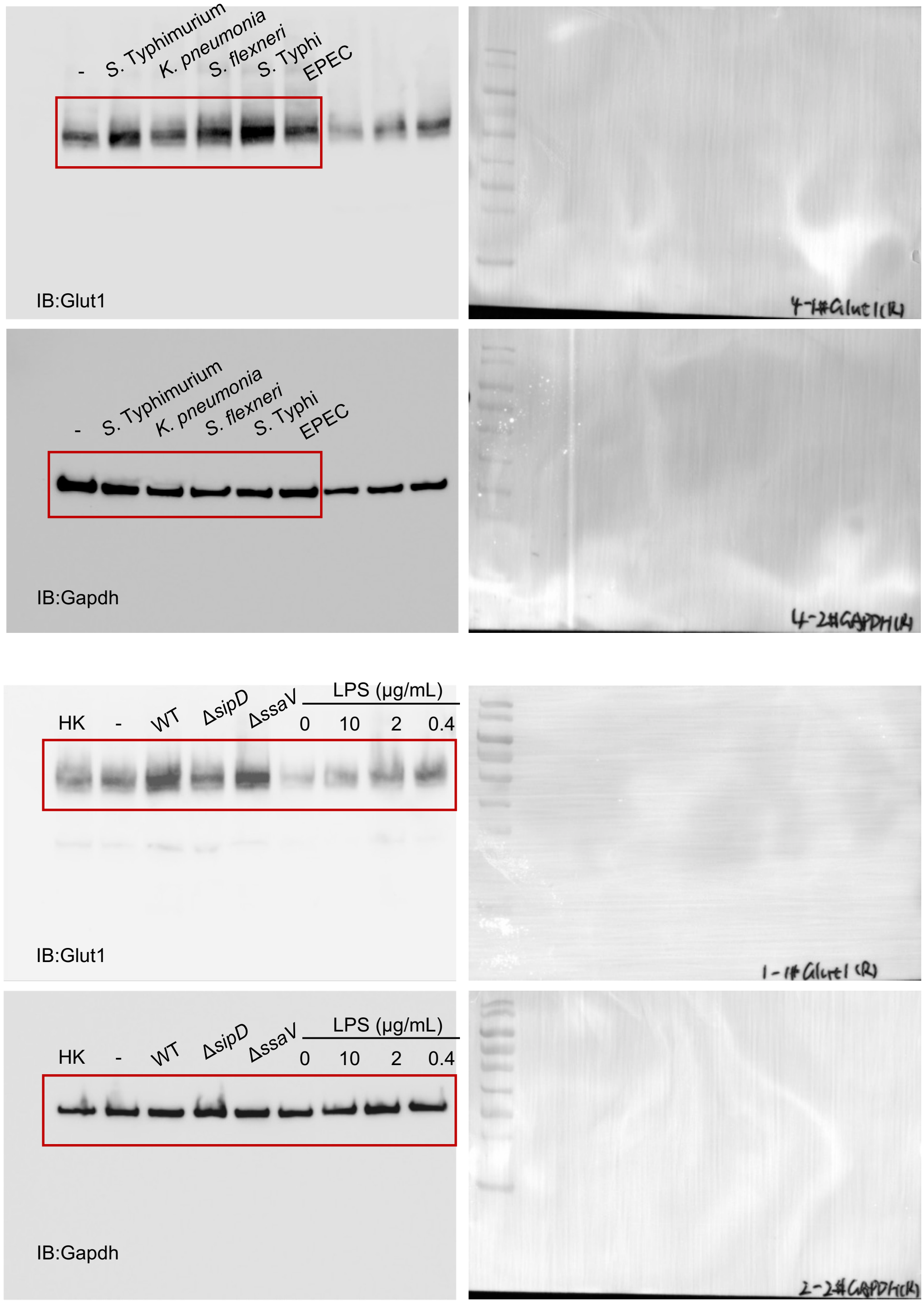

Figure 3C left

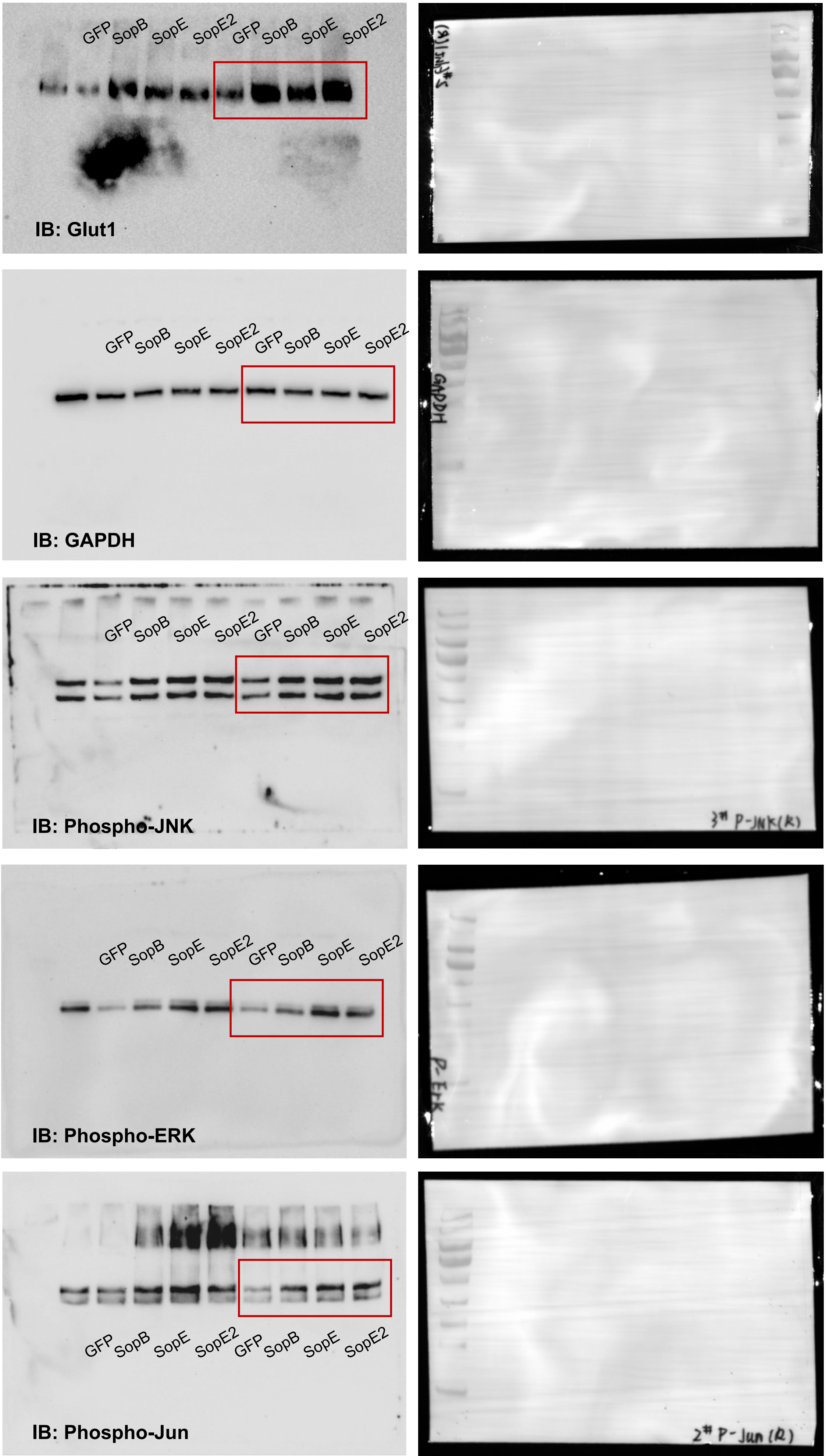

Figure 3C right

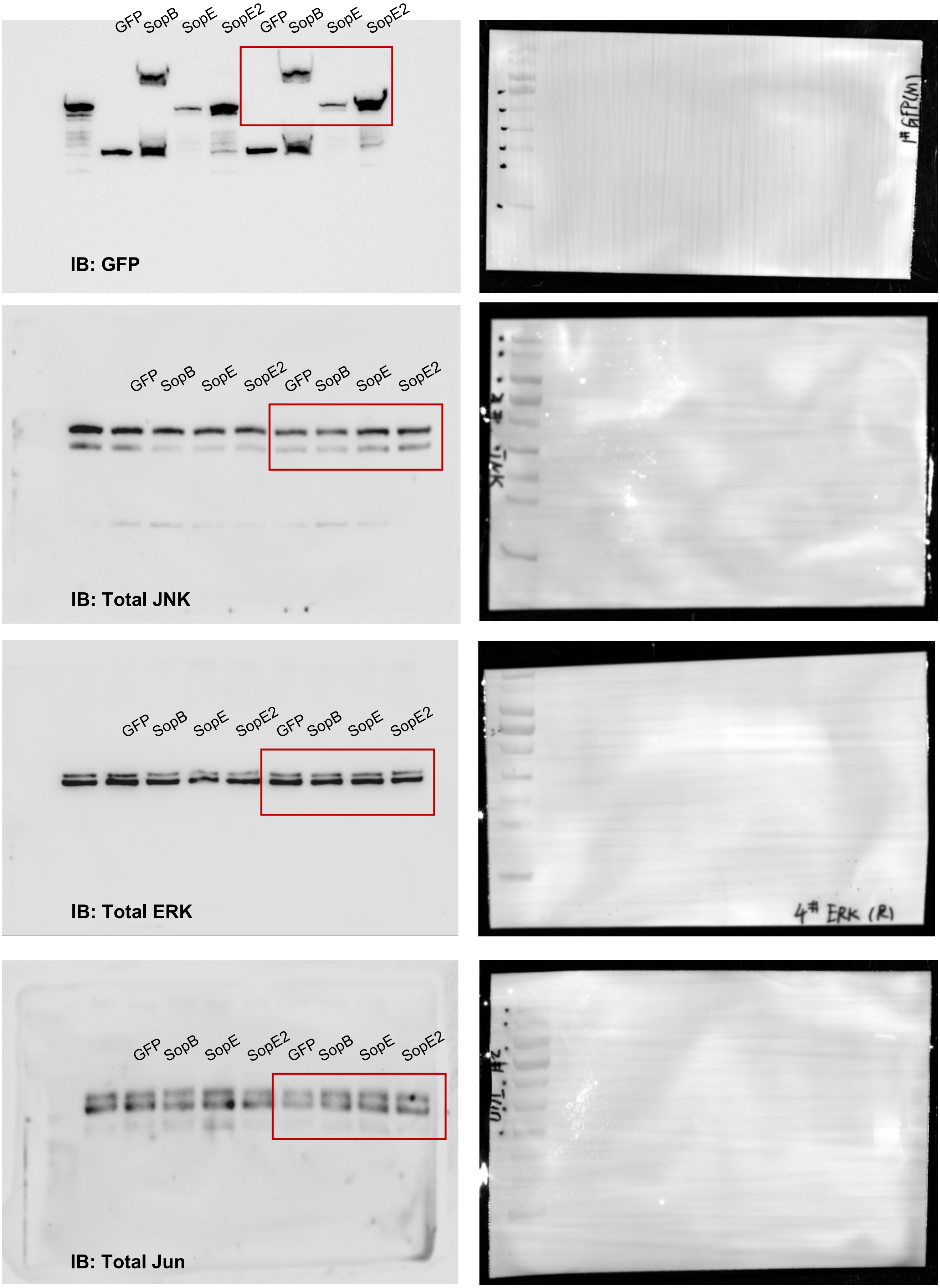

Figure 4F

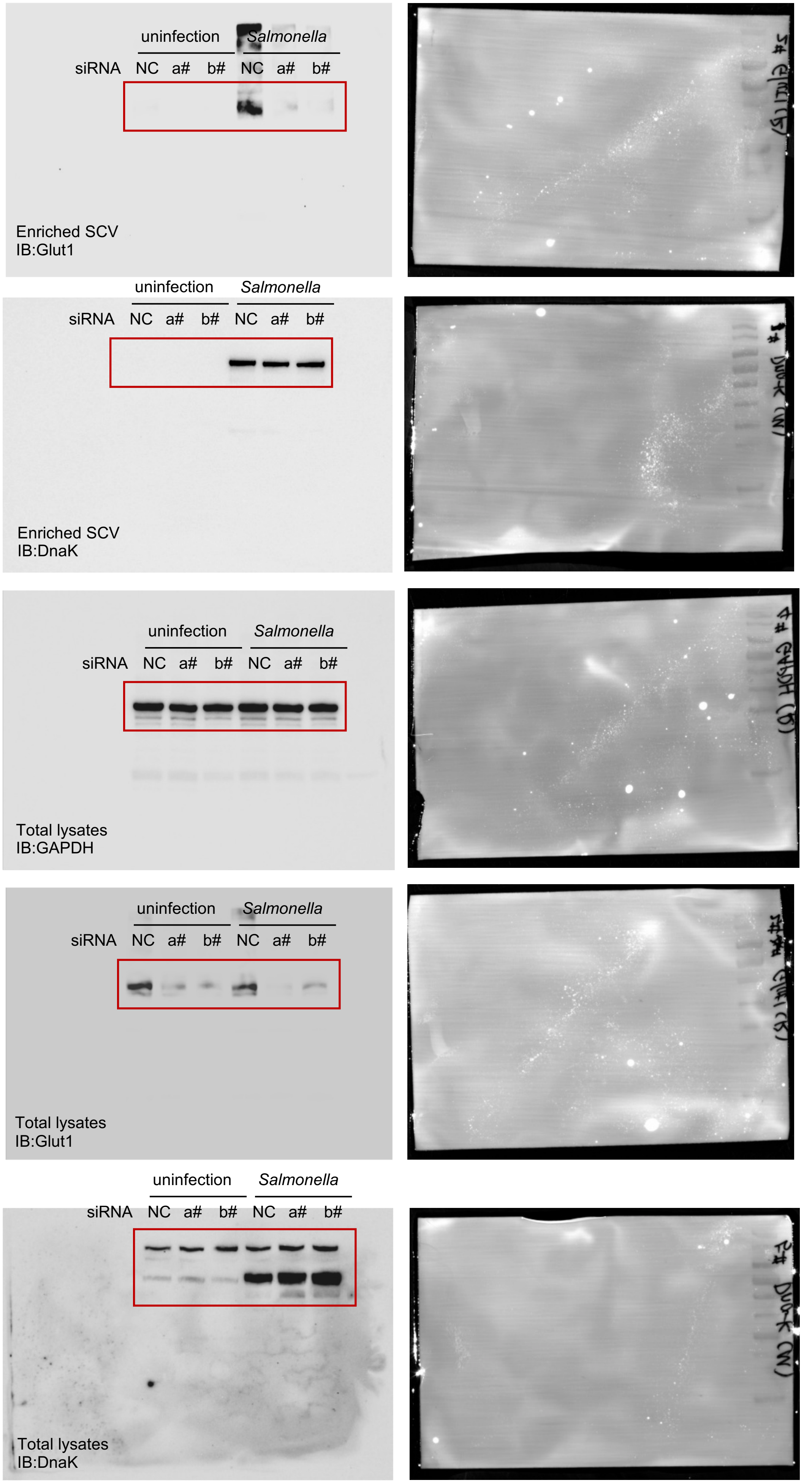
uninfection
Salmonella
siRNA NC a# b# NC a# b#
Enriched SCV
IB:Glut1
uninfection
Salmonella
siRNA NC a# b# NC a# b#
Enriched SCV
IB:DnaK
uninfection
Salmonella
siRNA NC a# b# NC a# b#
Total lysates
IB:GAPDH
uninfection
Salmonella
siRNA NC a# b# NC a# b#
Total lysates
IB:Glut1
uninfection
Salmonella
siRNA NC a# b# NC a# b#
Total lysates
IB:DnaK

Figure 5A

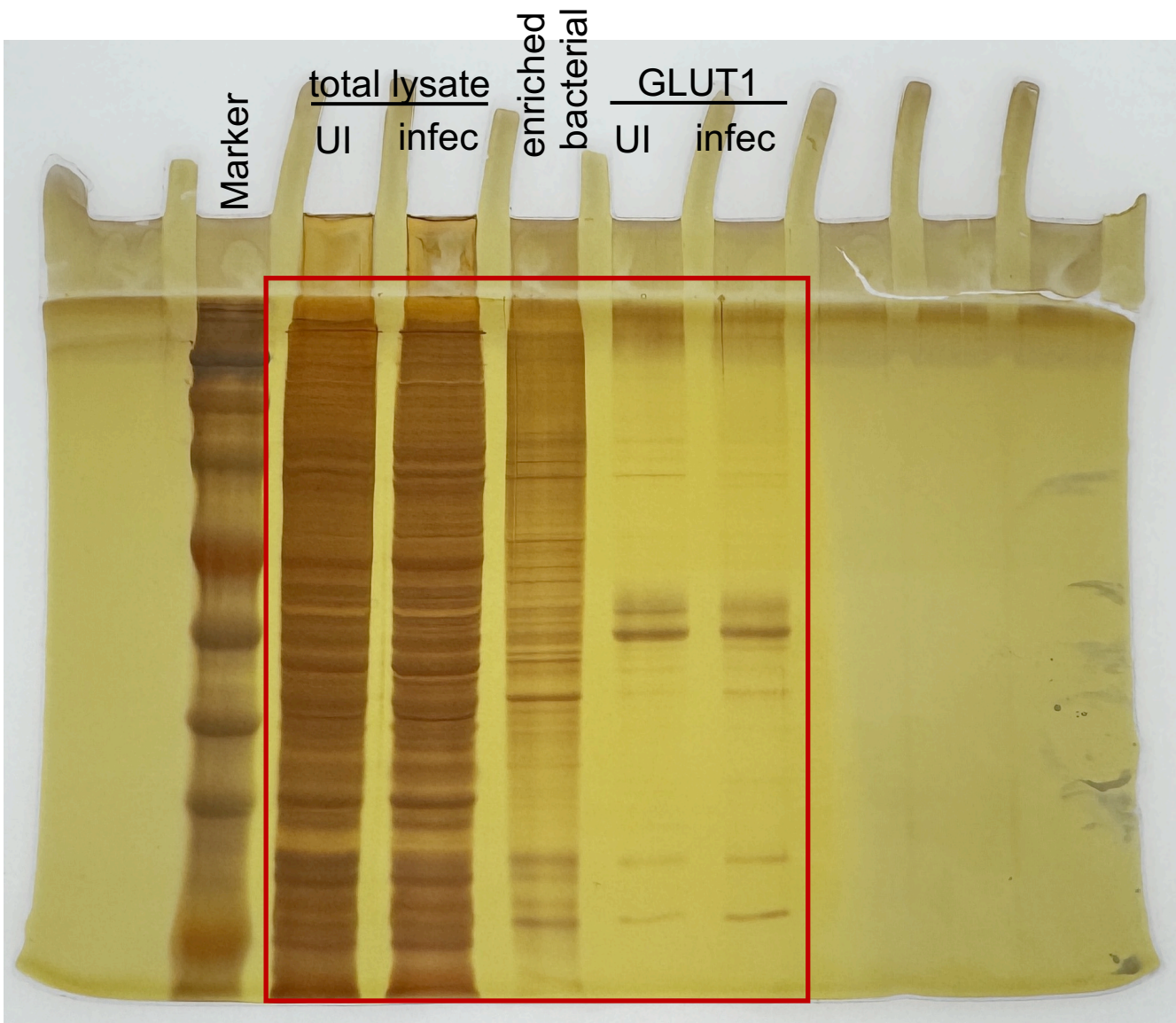

Figure 5C-1

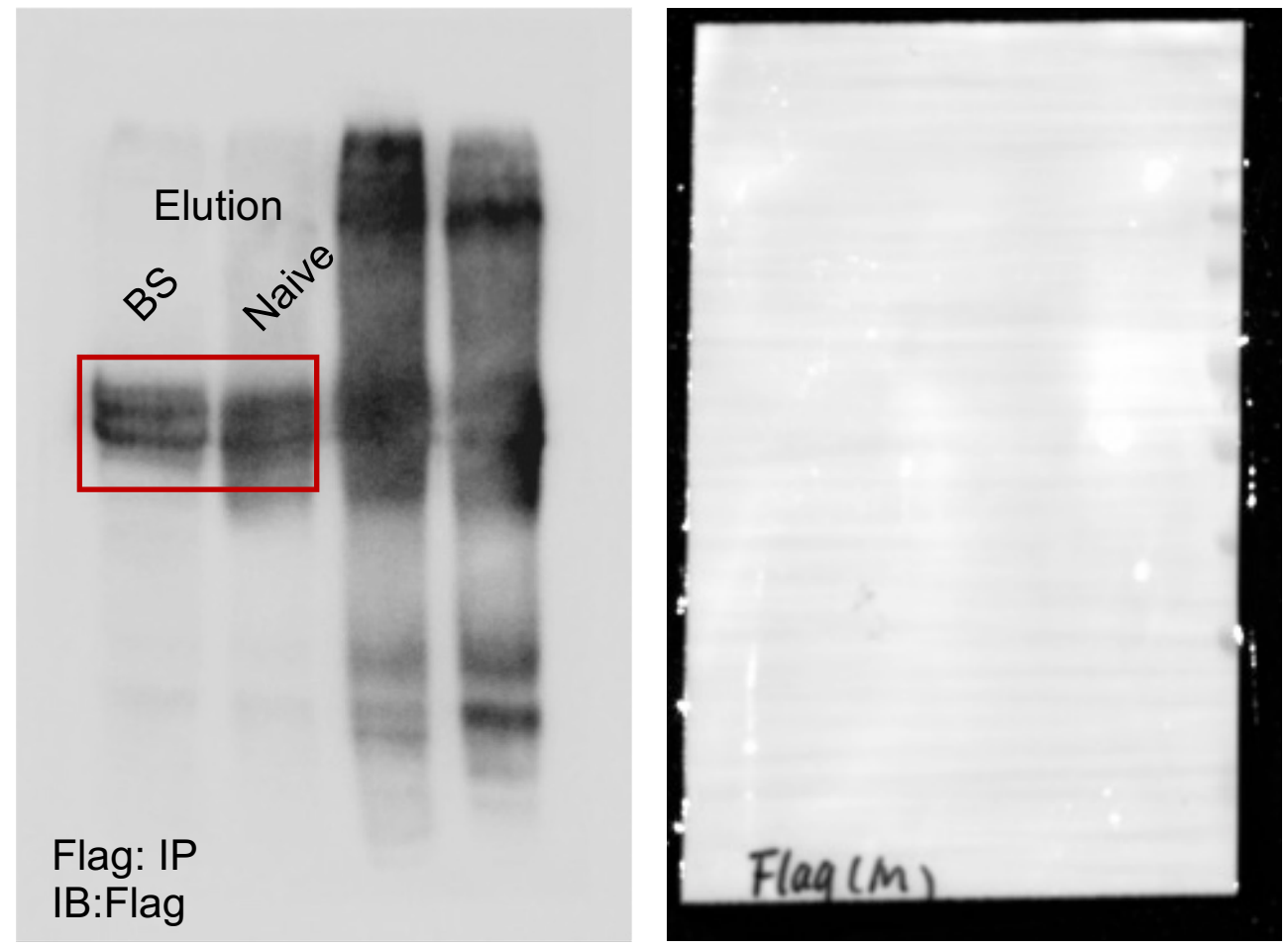

Figure 5C-2

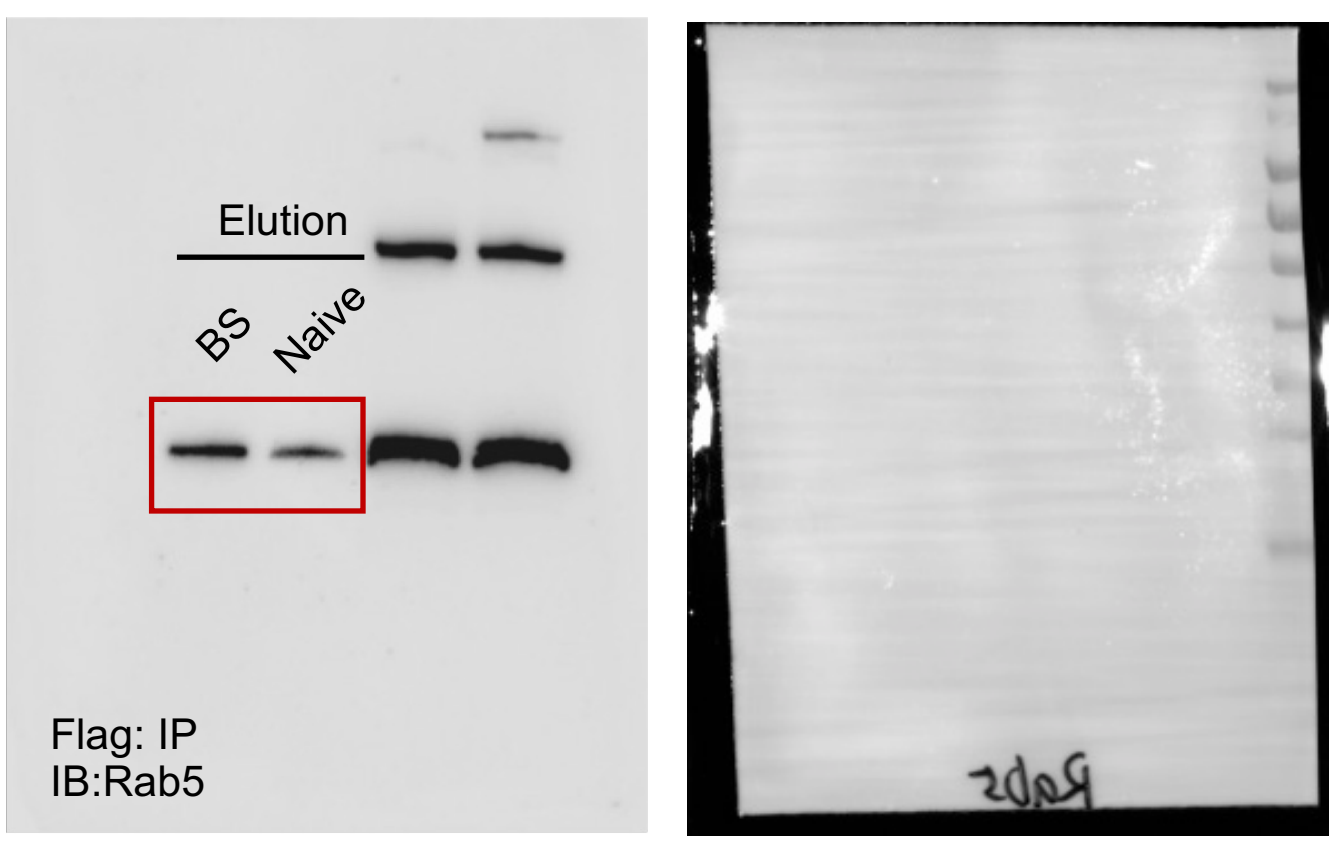

Figure 5C-3

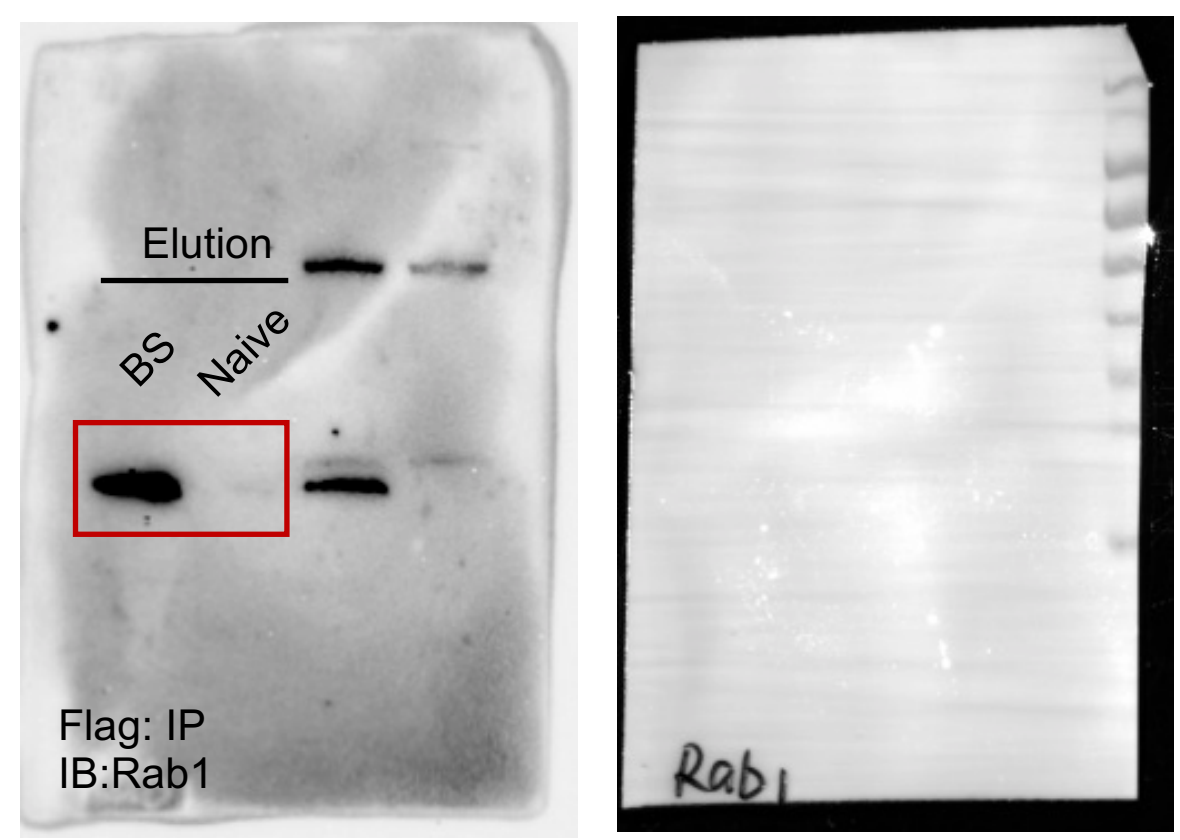

Figure 5G

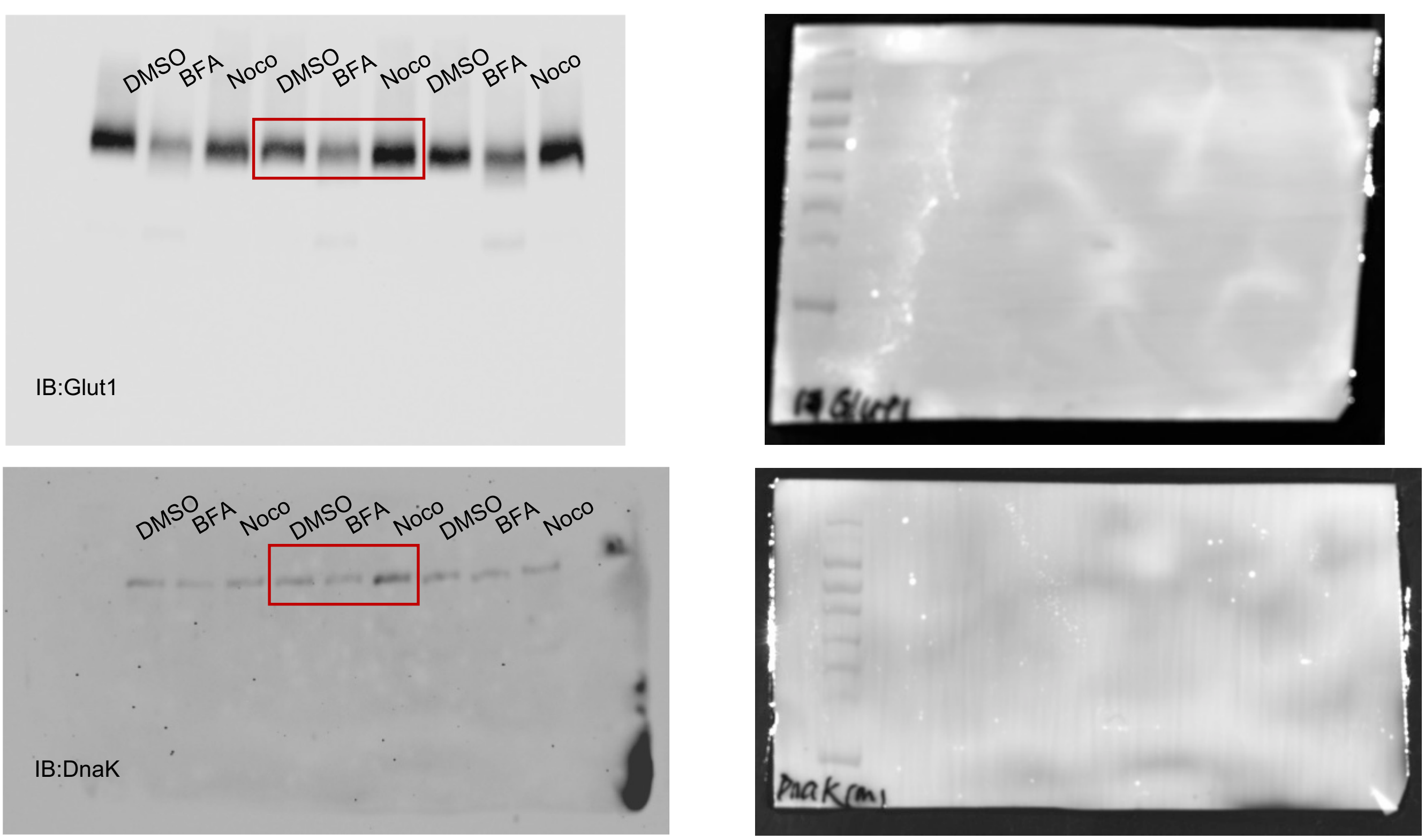

Figure 6A

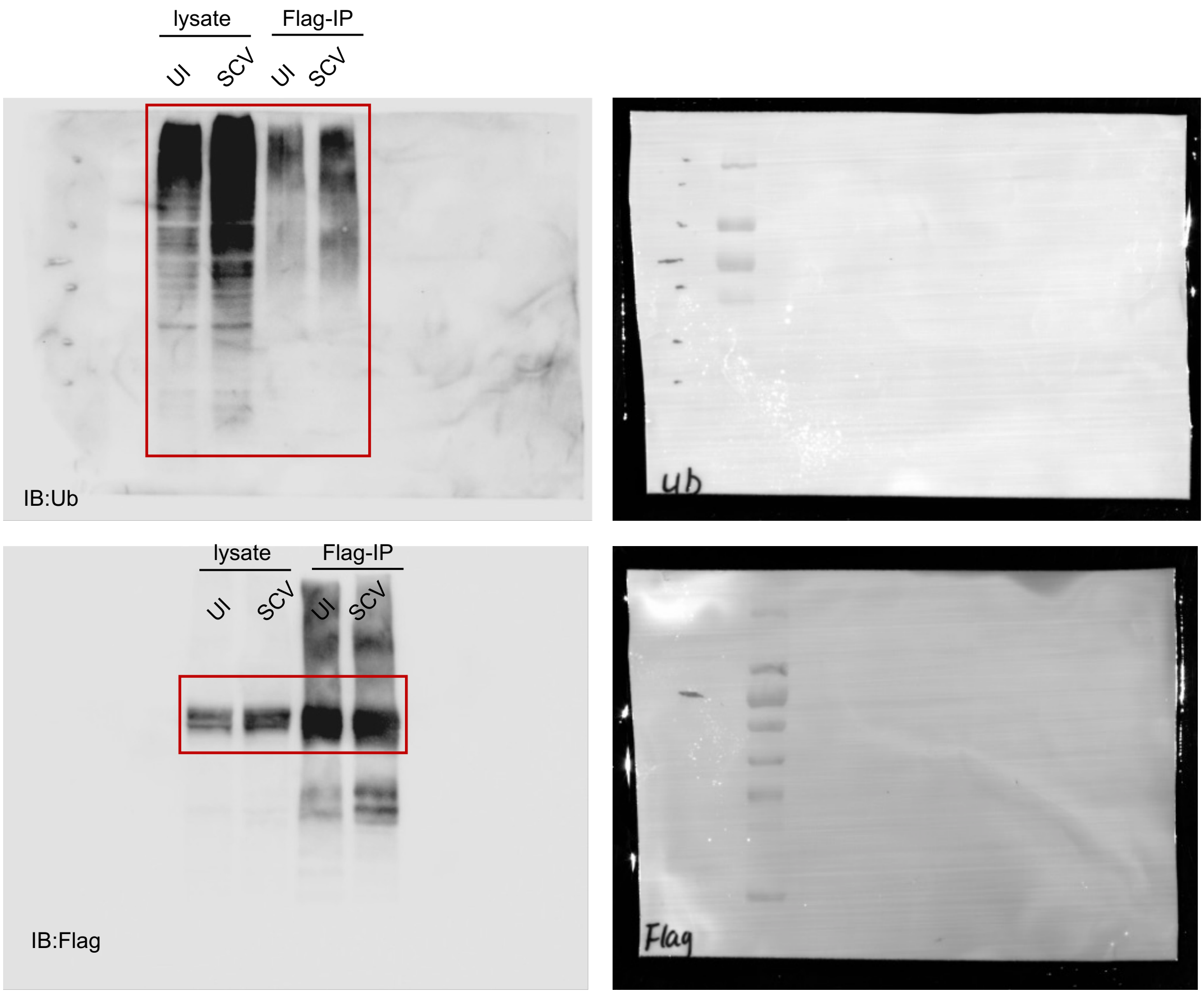

Figure 6F

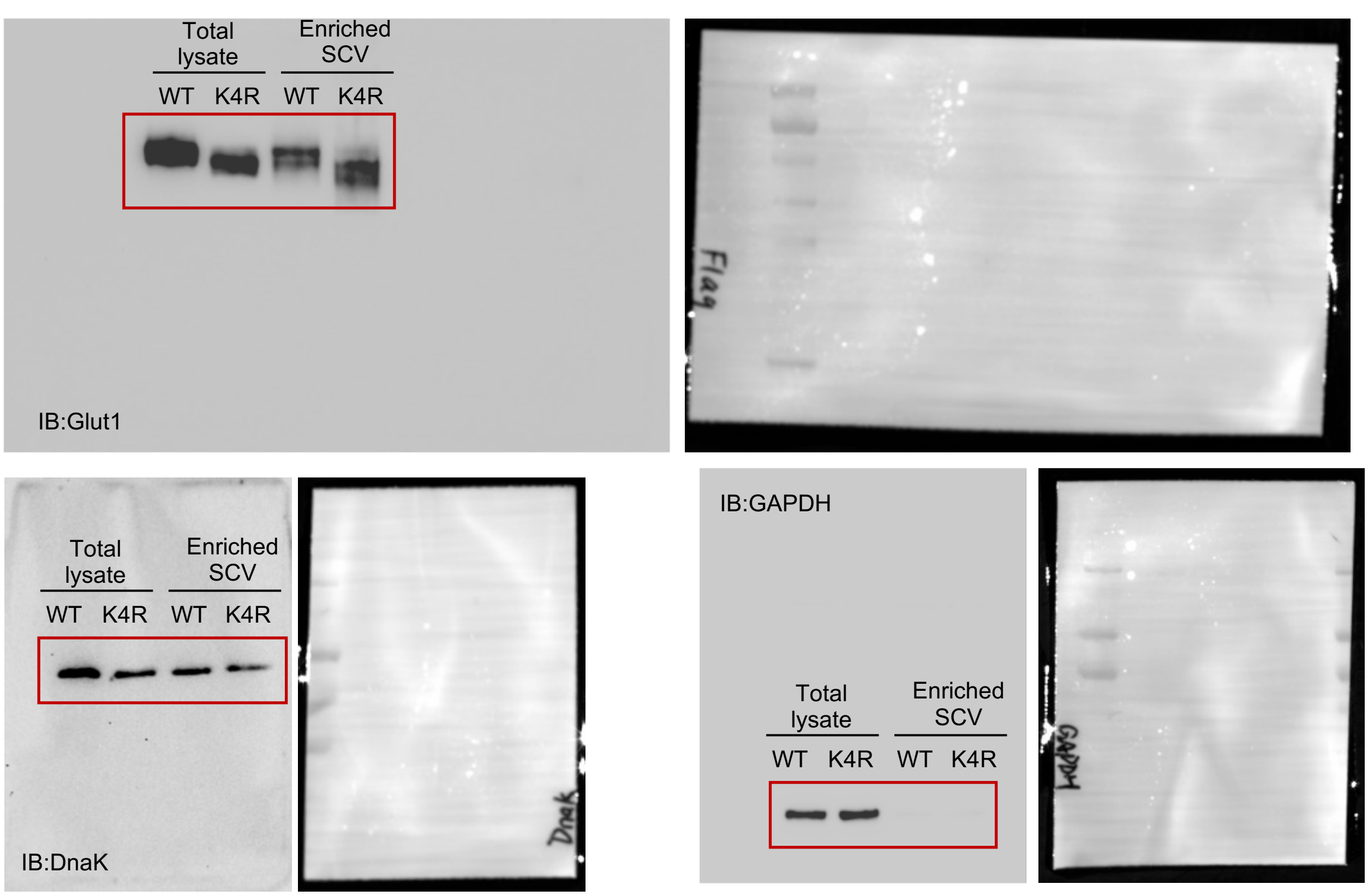

Figure 6D

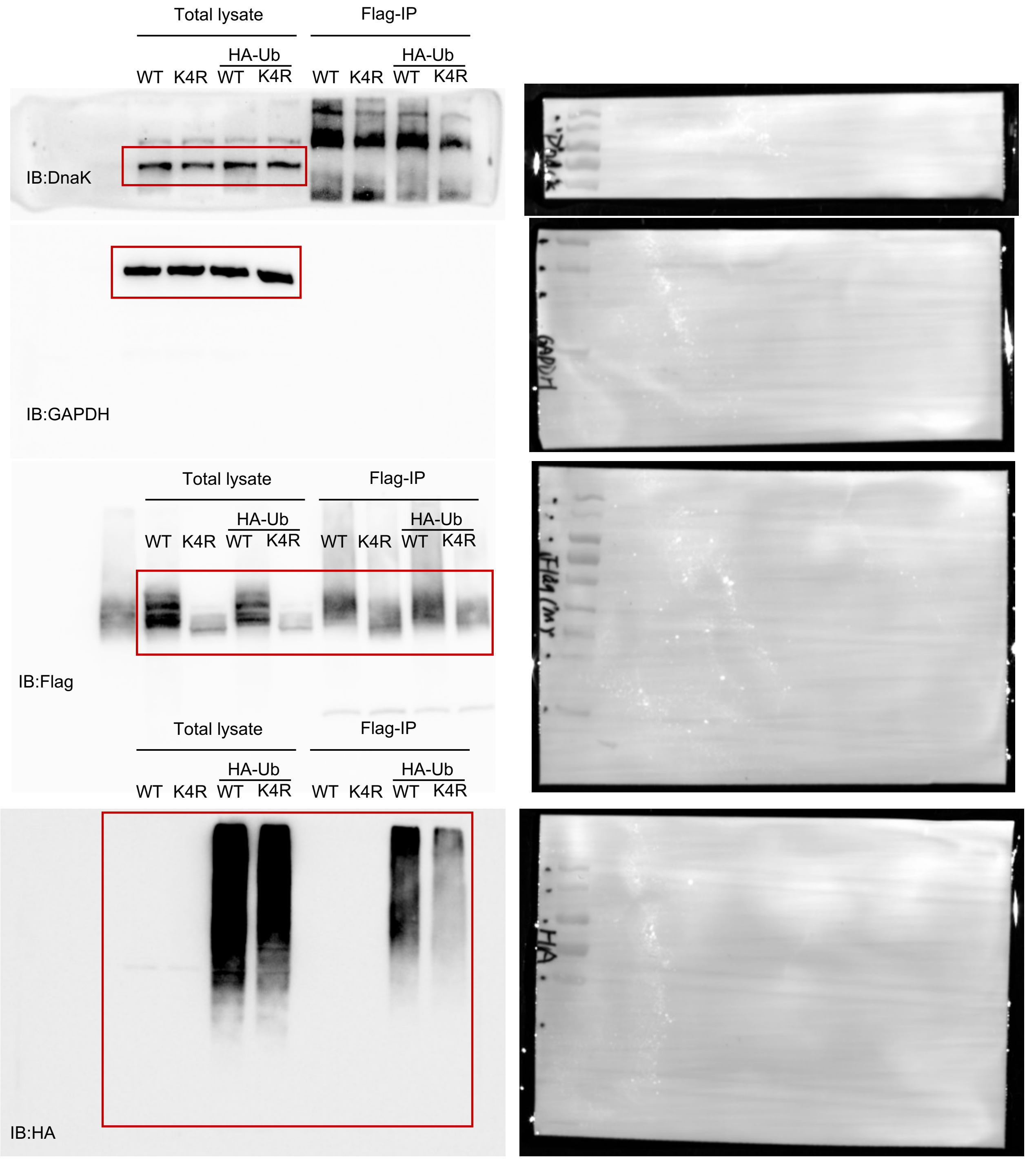

Total lysate
Flag-IP
HA-Ub
HA-Ub
WT K4R WT K4R WT K4R WT K4R
IB:DnaK
IB:GAPDH
Total lysate
Flag-IP
HA-Ub
HA-Ub
WT K4R WT K4R WT K4R WT K4R
IB:Flag
Total lysate
Flag-IP
HA-Ub
HA-Ub
WT K4R WT K4R WT K4R WT K4R
IB:HA

Figure 7A (Total lysate)

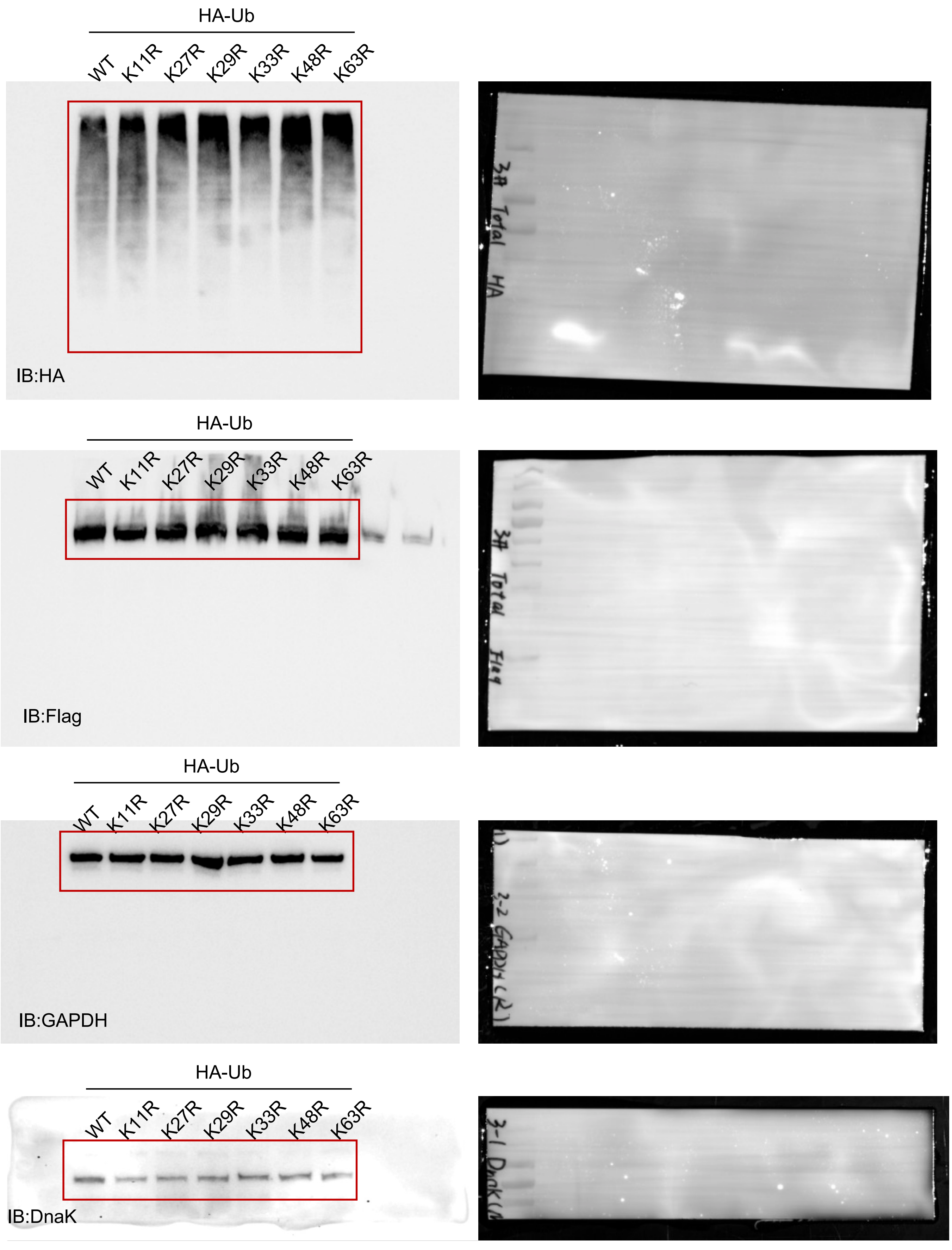

Figure 7A (Flag-IP)

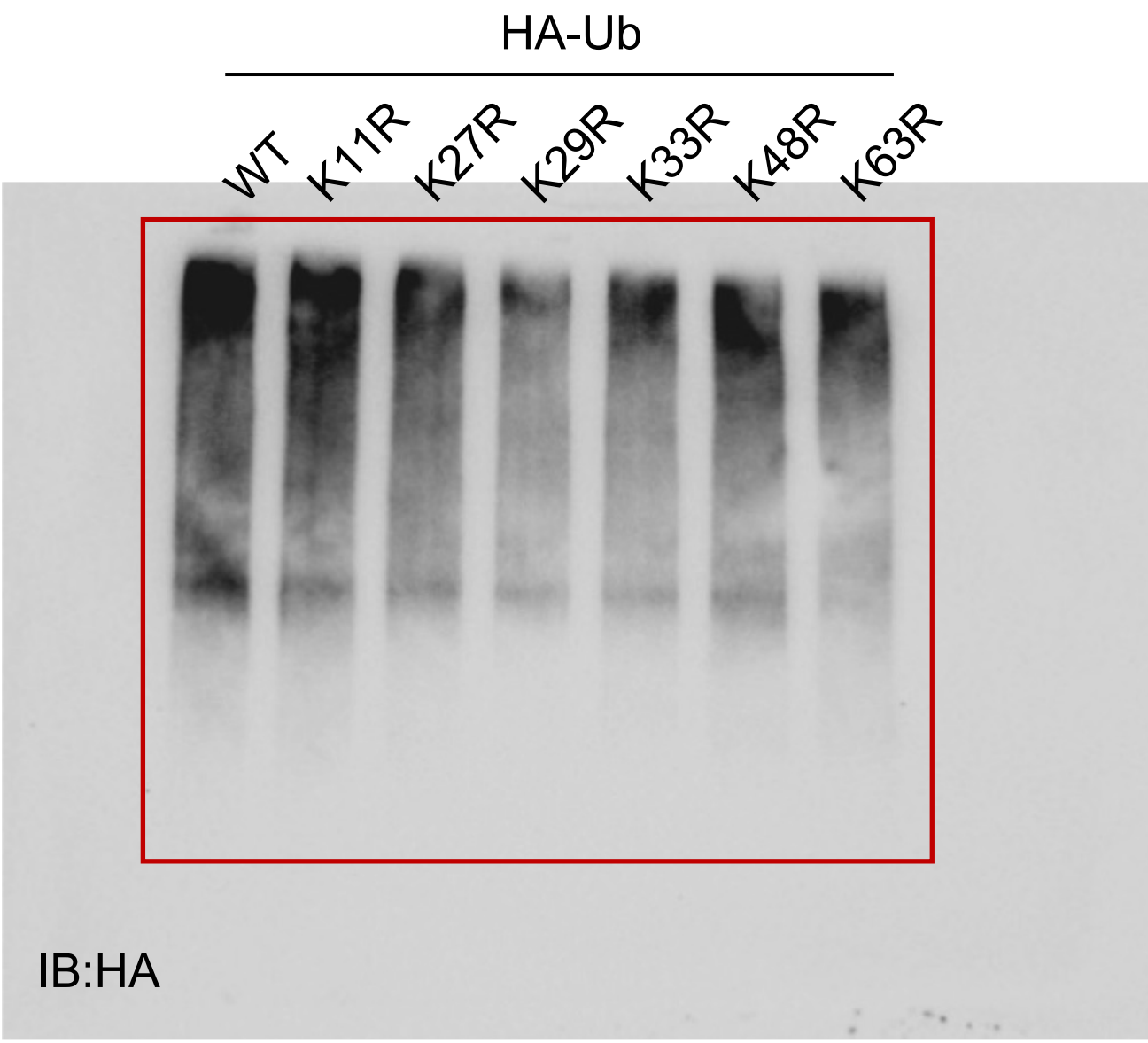

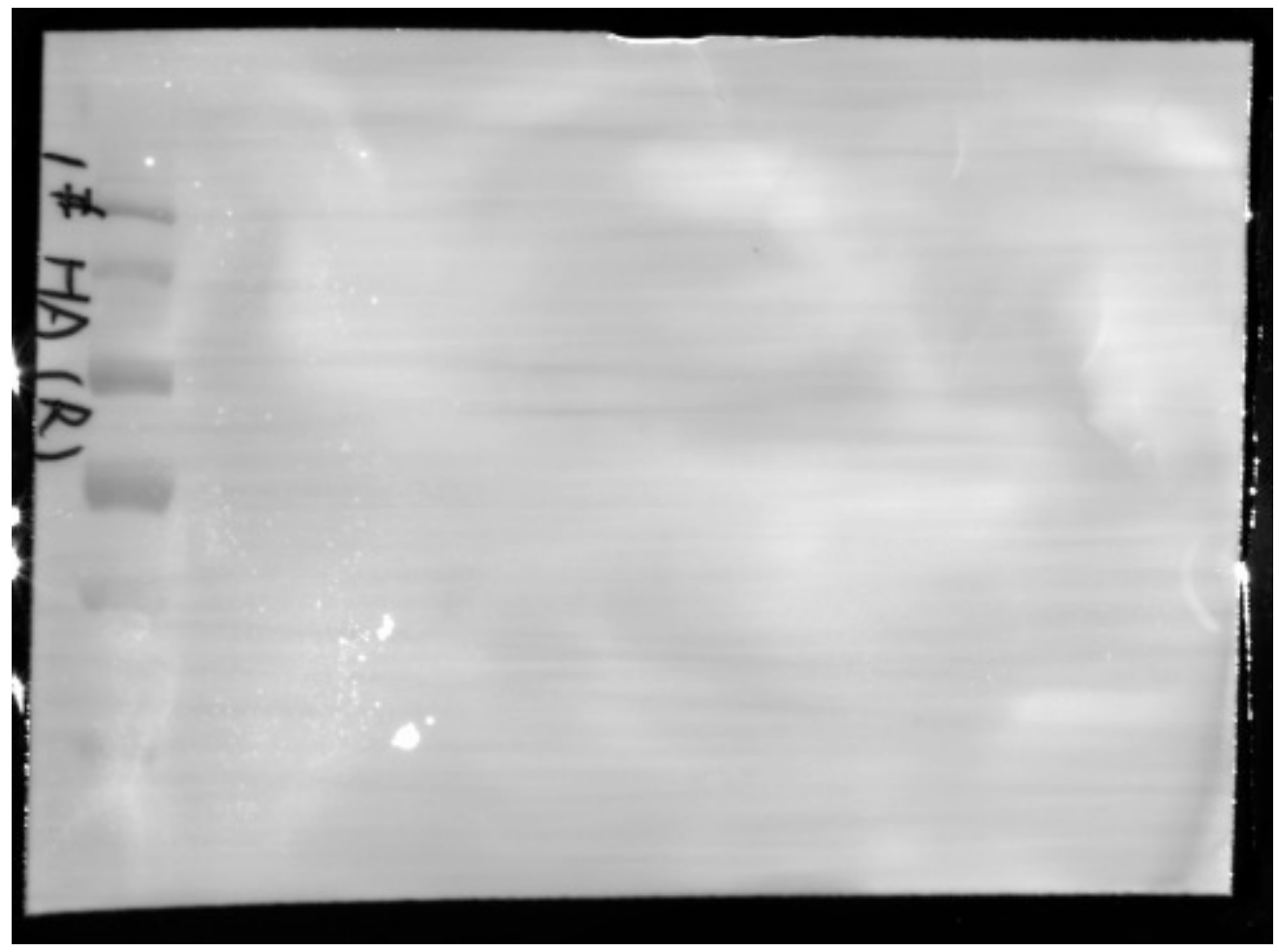

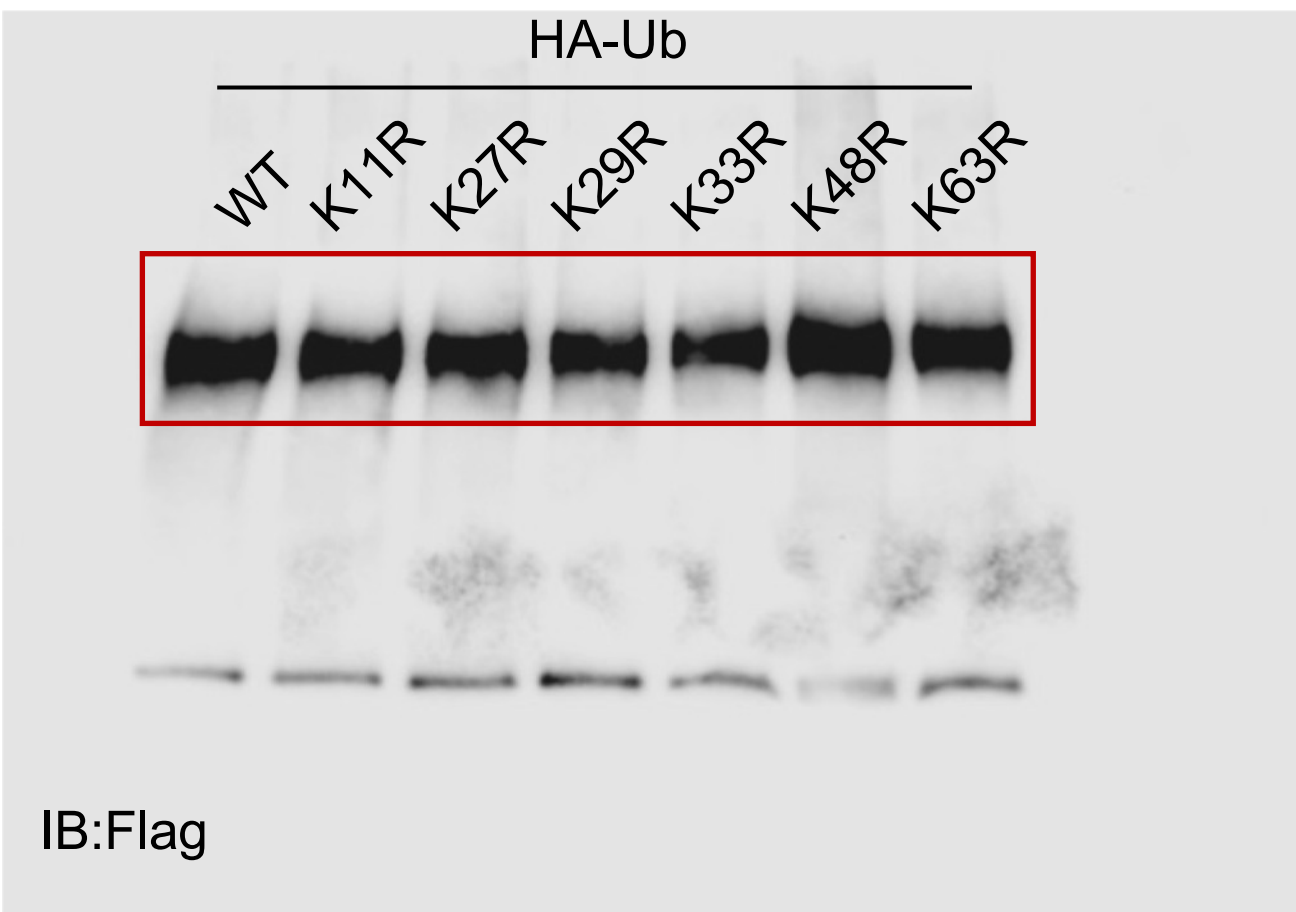

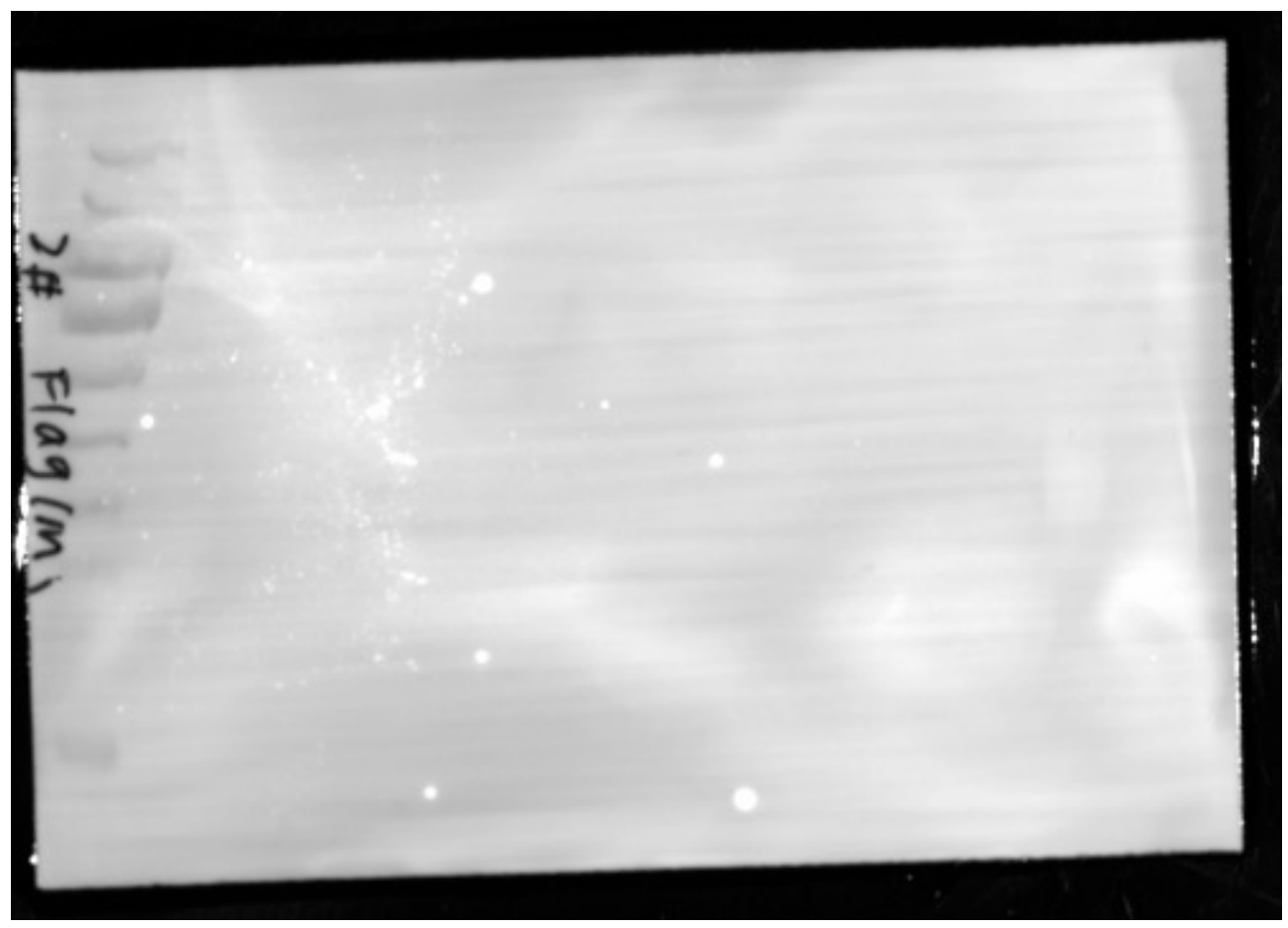

Figure 7B

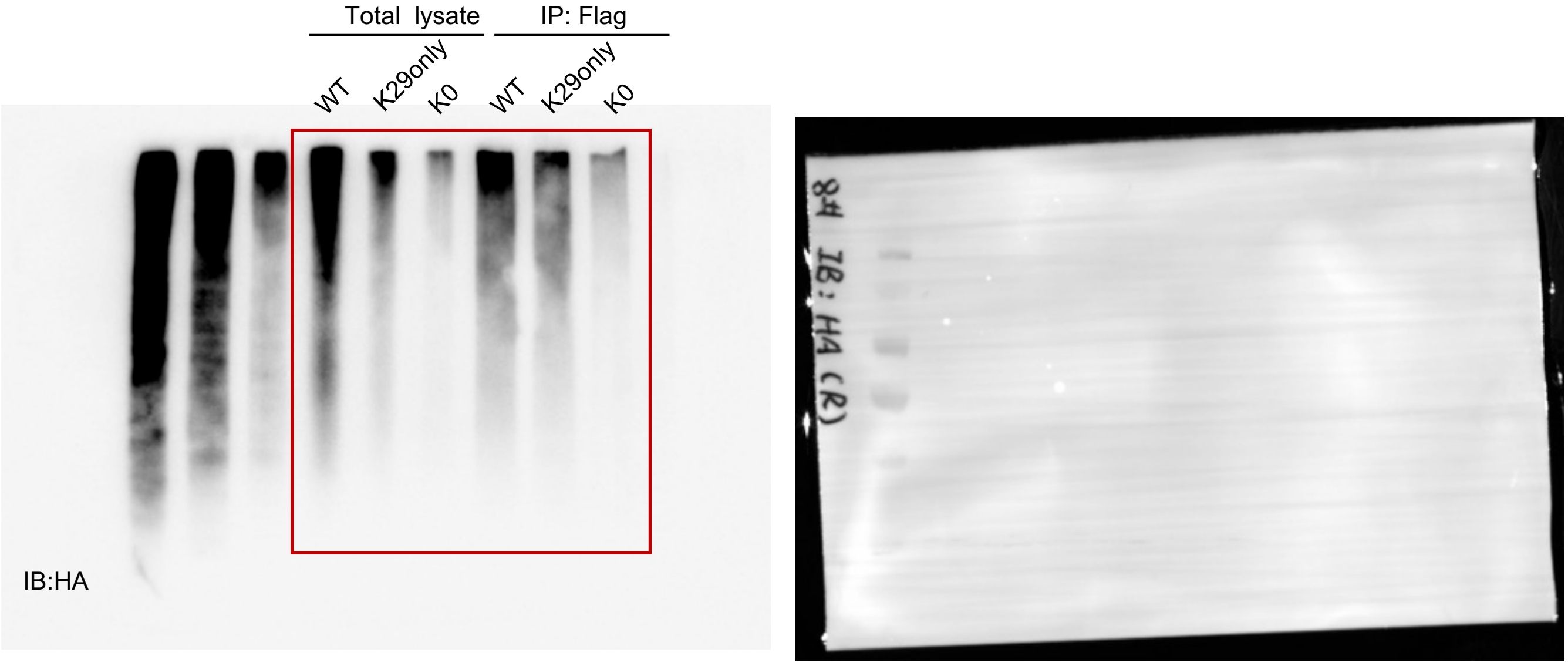

Total lysate
IP: Flag
WT
K29only
K0
WT
K29only
K0
IB:HA

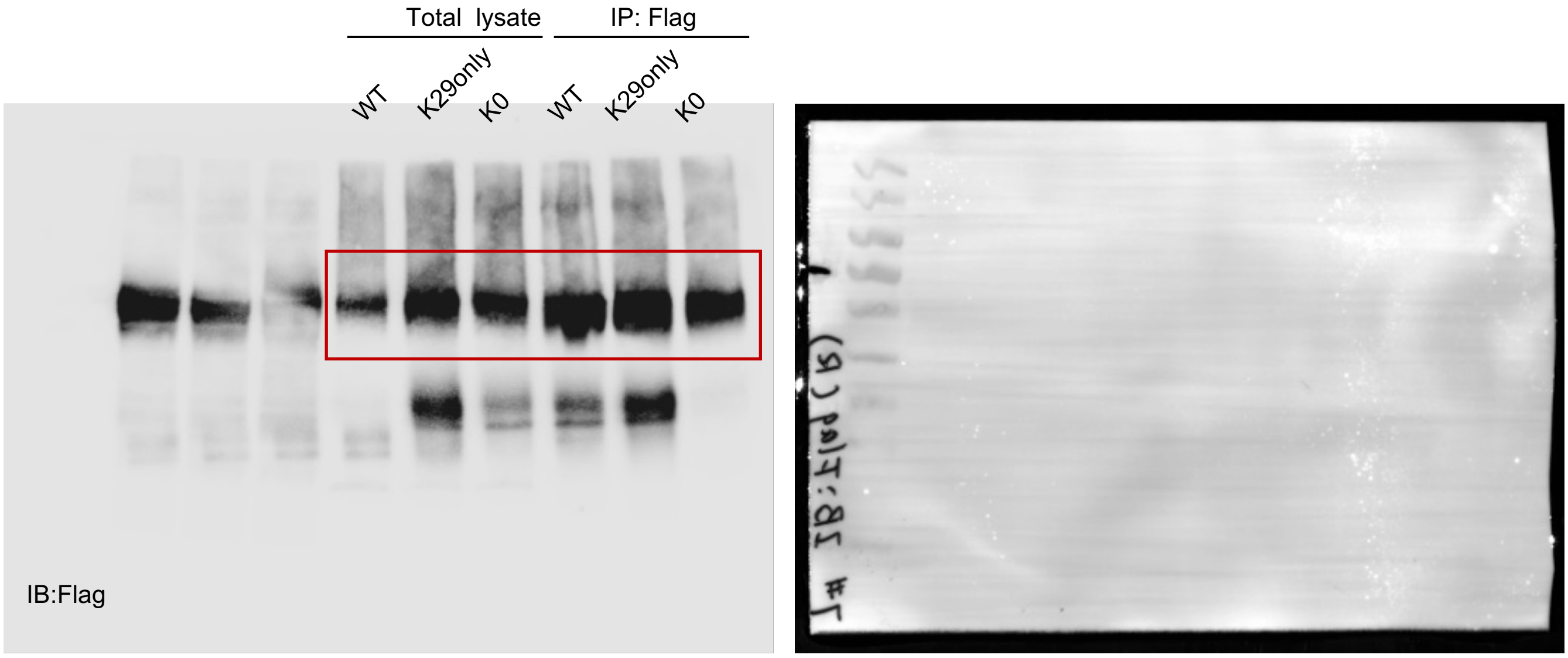

Total lysate
IP: Flag
WT
K29only
K0
WT
K29only
K0
IB:Flag

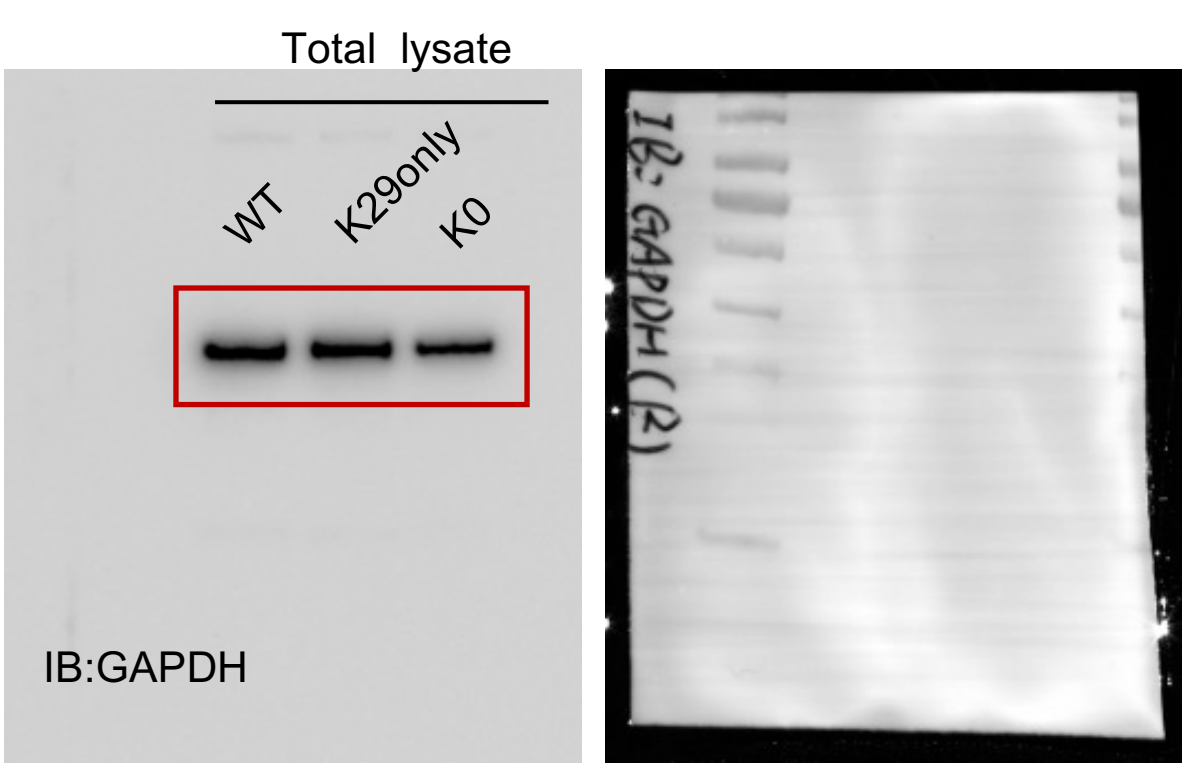

Total lysate
WT
K29only
K0
IB:GAPDH

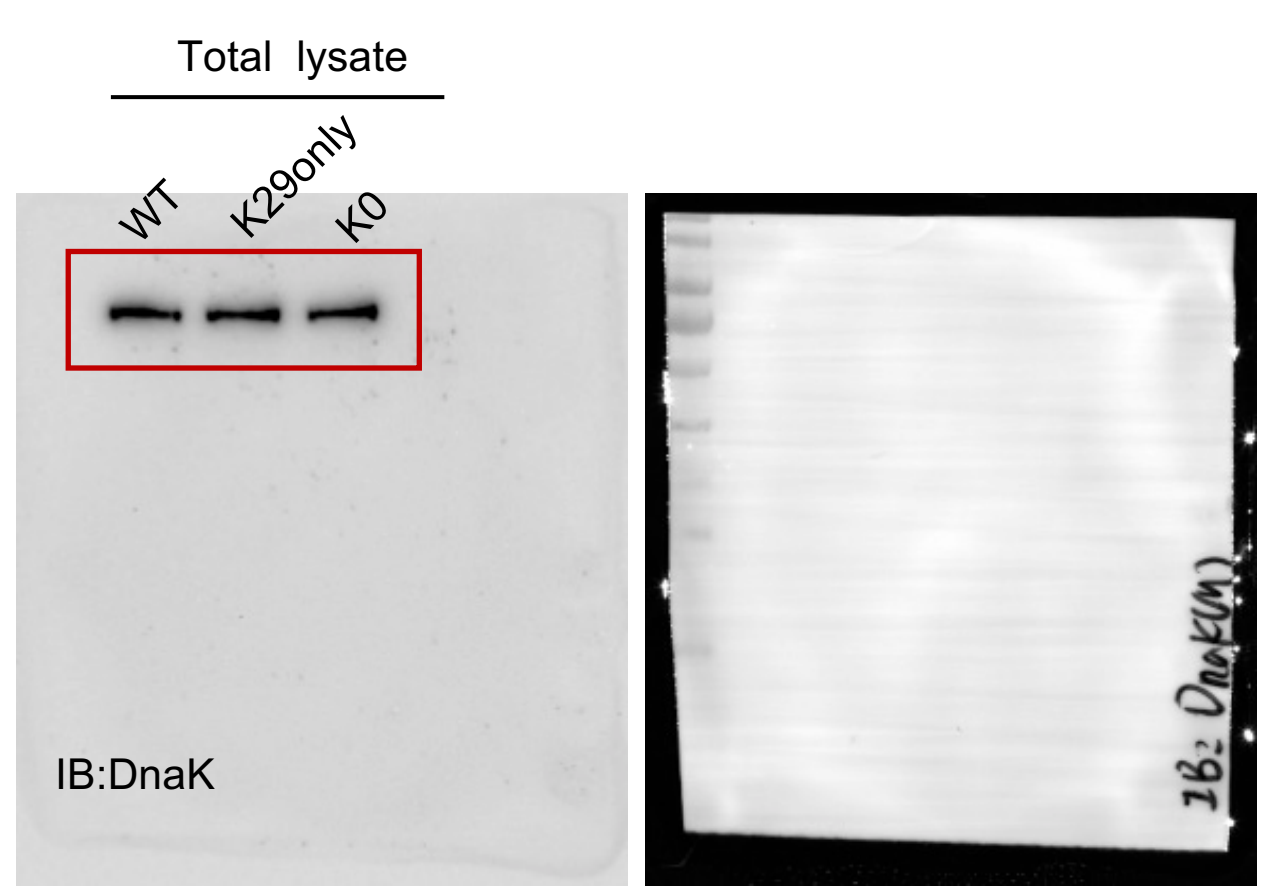

Total lysate
WT
K29only
K0
IB:DnaK

Figure 7F

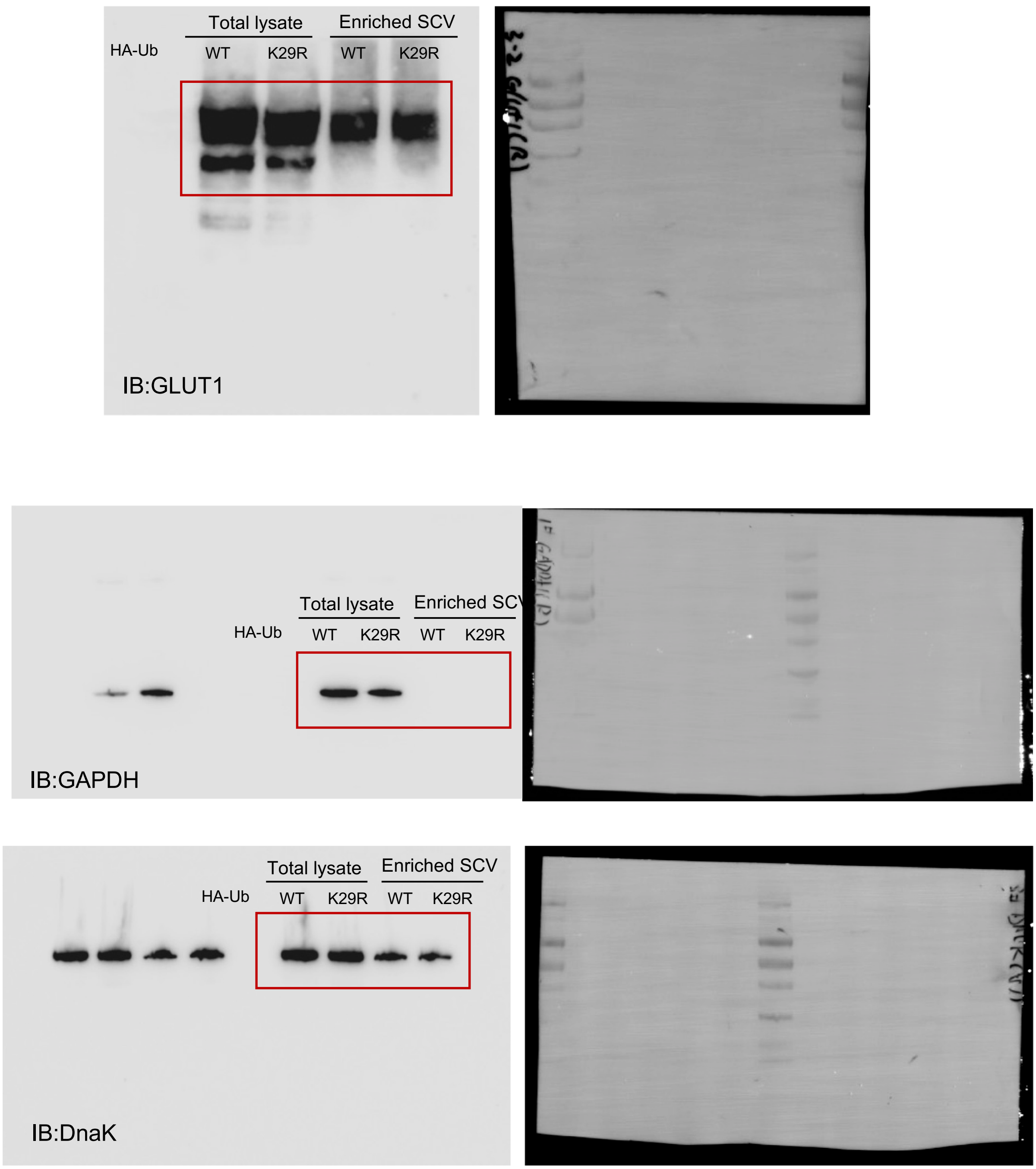
Total lysate
Enriched SCV
HA-Ub
WT
K29R
WT
K29R
IB:GLUT1
Total lysate
Enriched SCV
HA-Ub
WT
K29R
WT
K29R
IB:GAPDH
Total lysate
Enriched SCV
HA-Ub
WT
K29R
WT
K29R
IB:DnaK

Figure 8A IB GLUT1

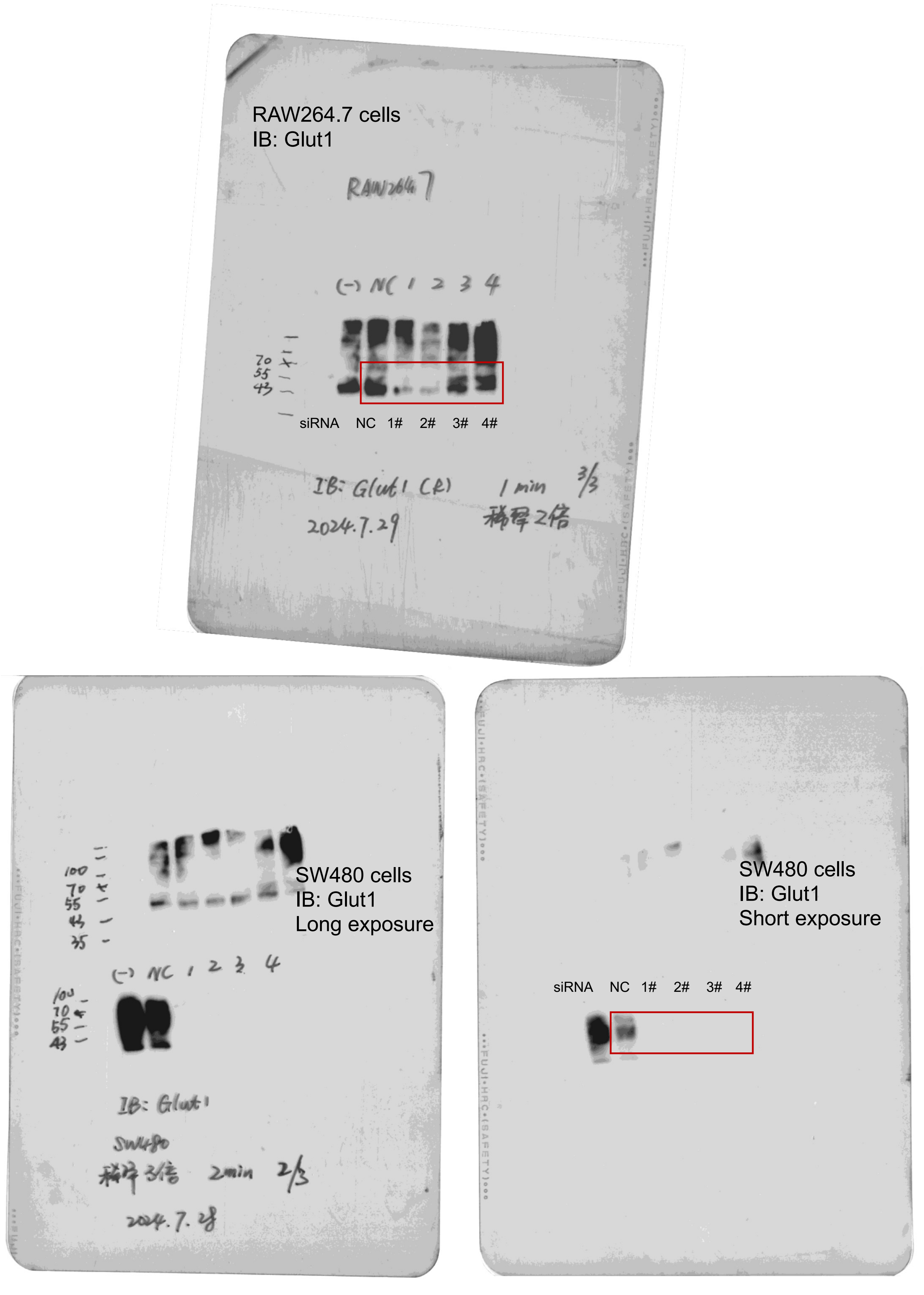

Figure 8A IB GAPDH

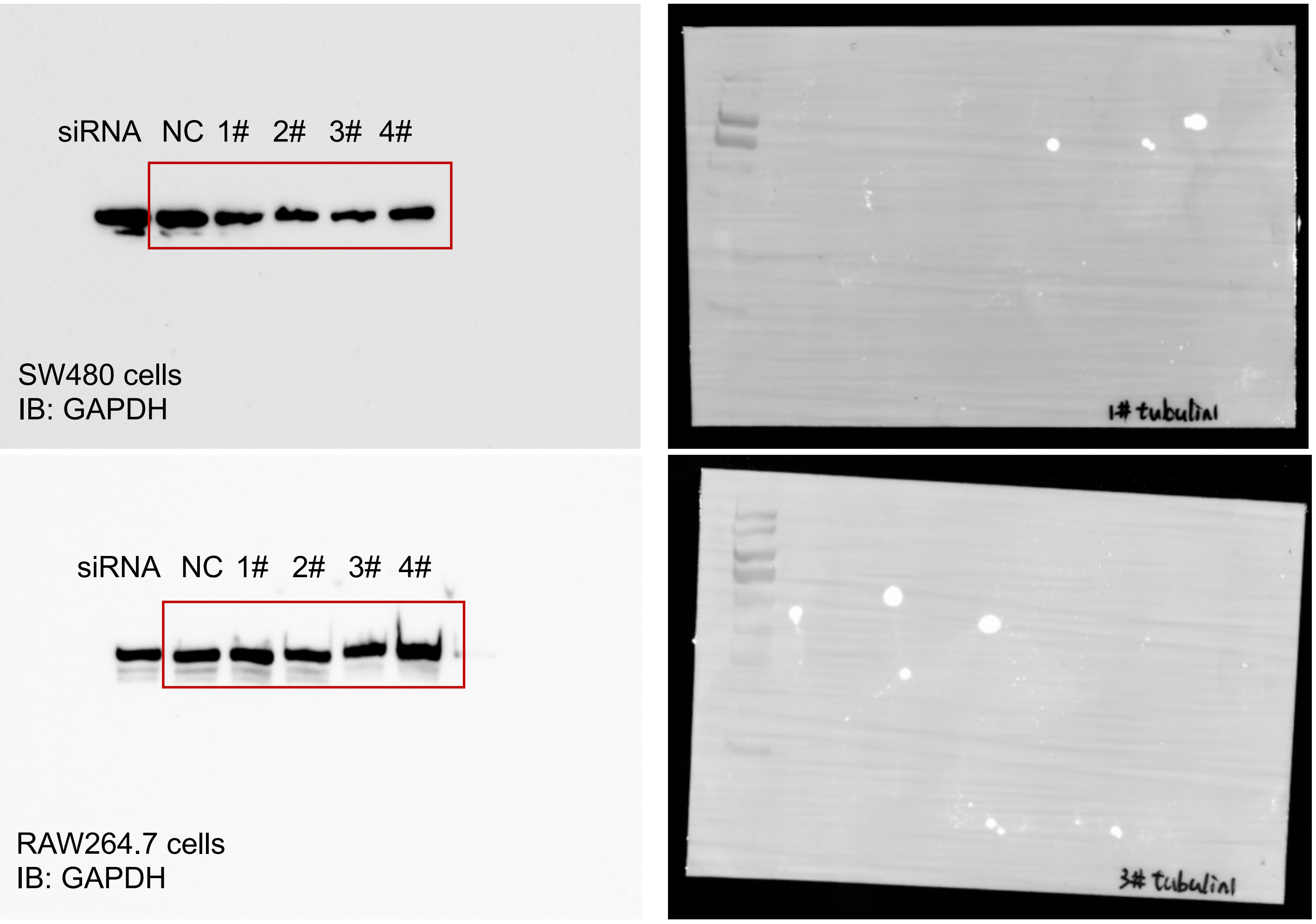

Figure 10D Liver

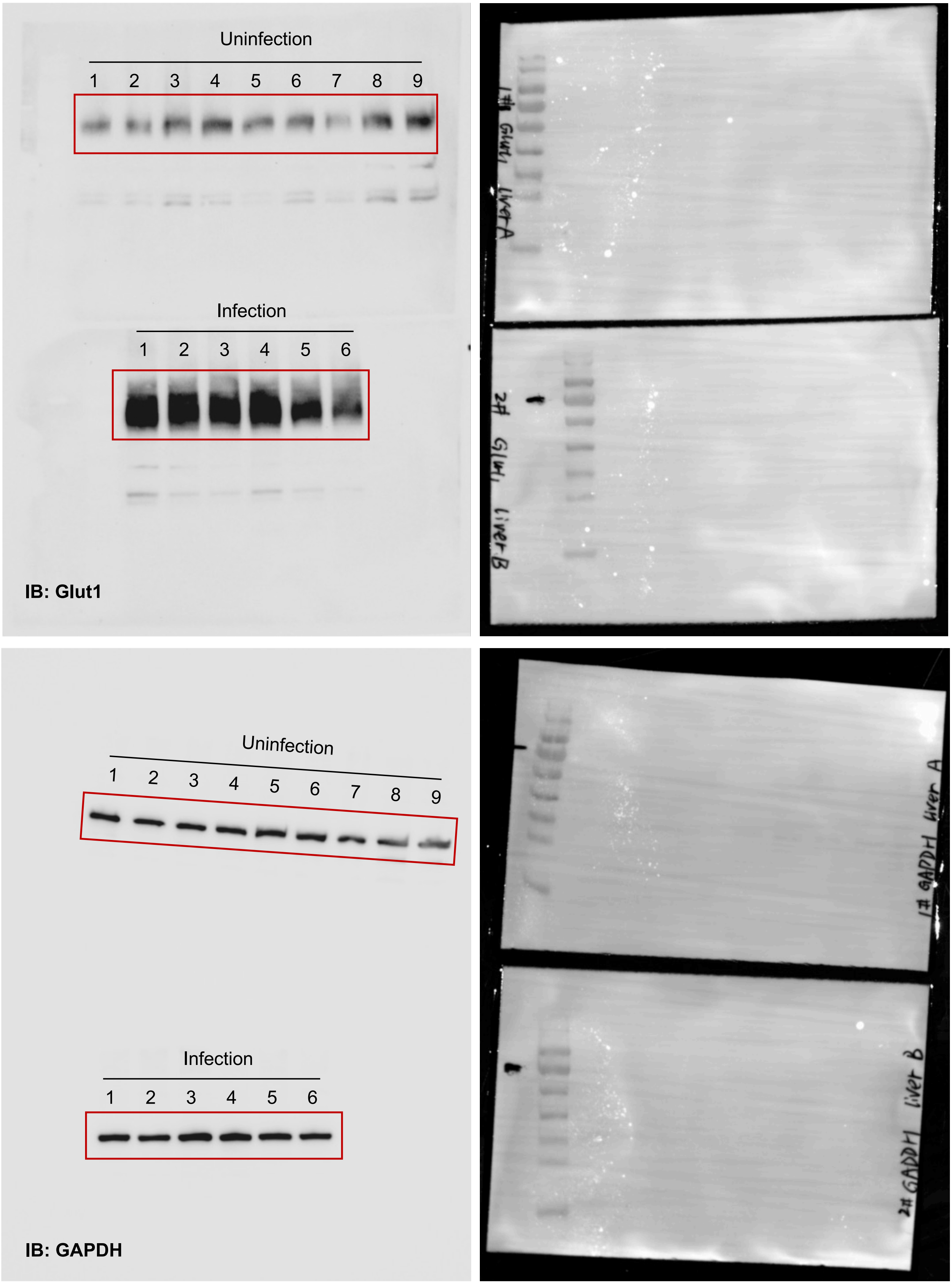

Figure 10D Spleen

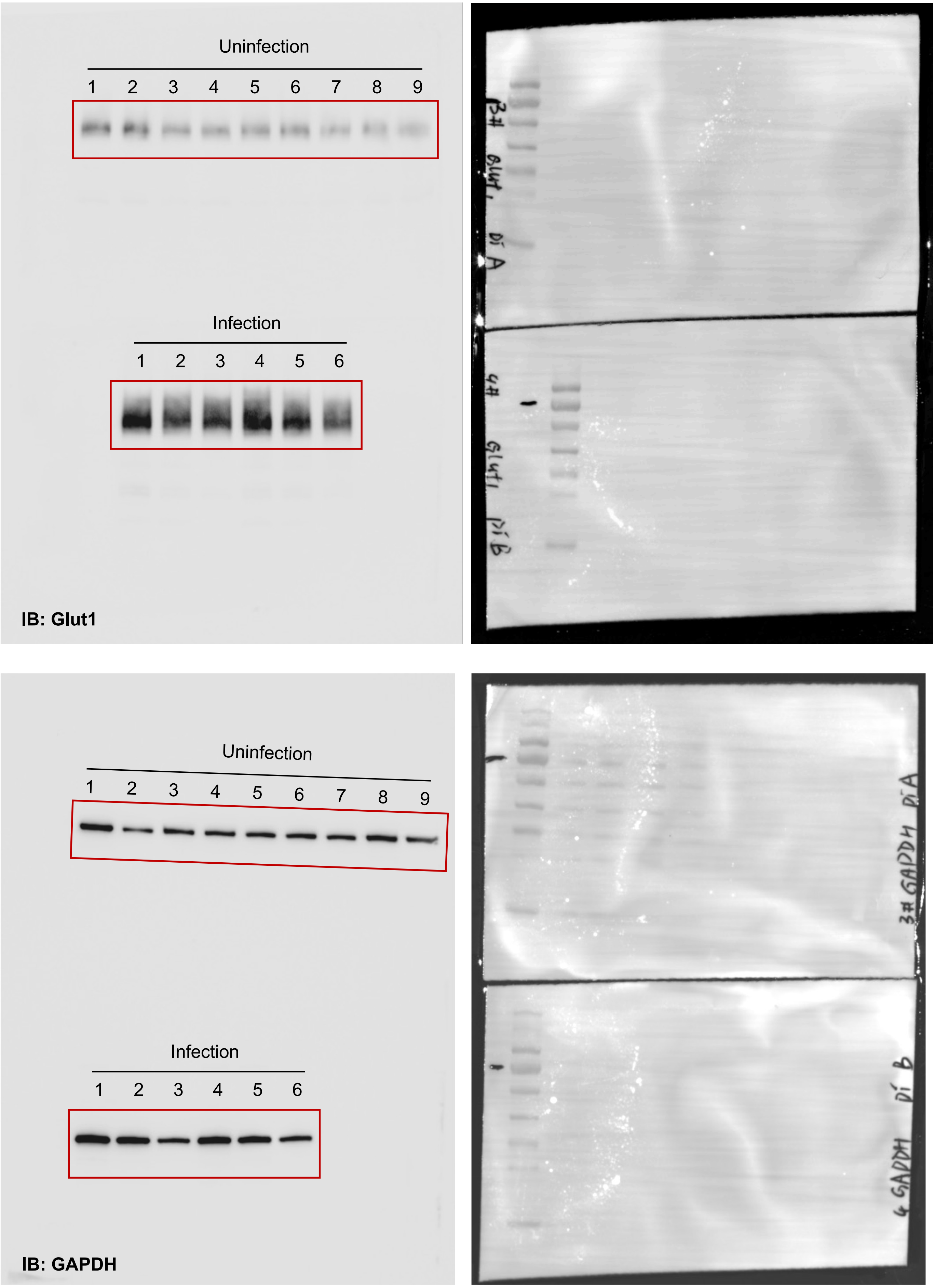

Supplementary Figure 2B

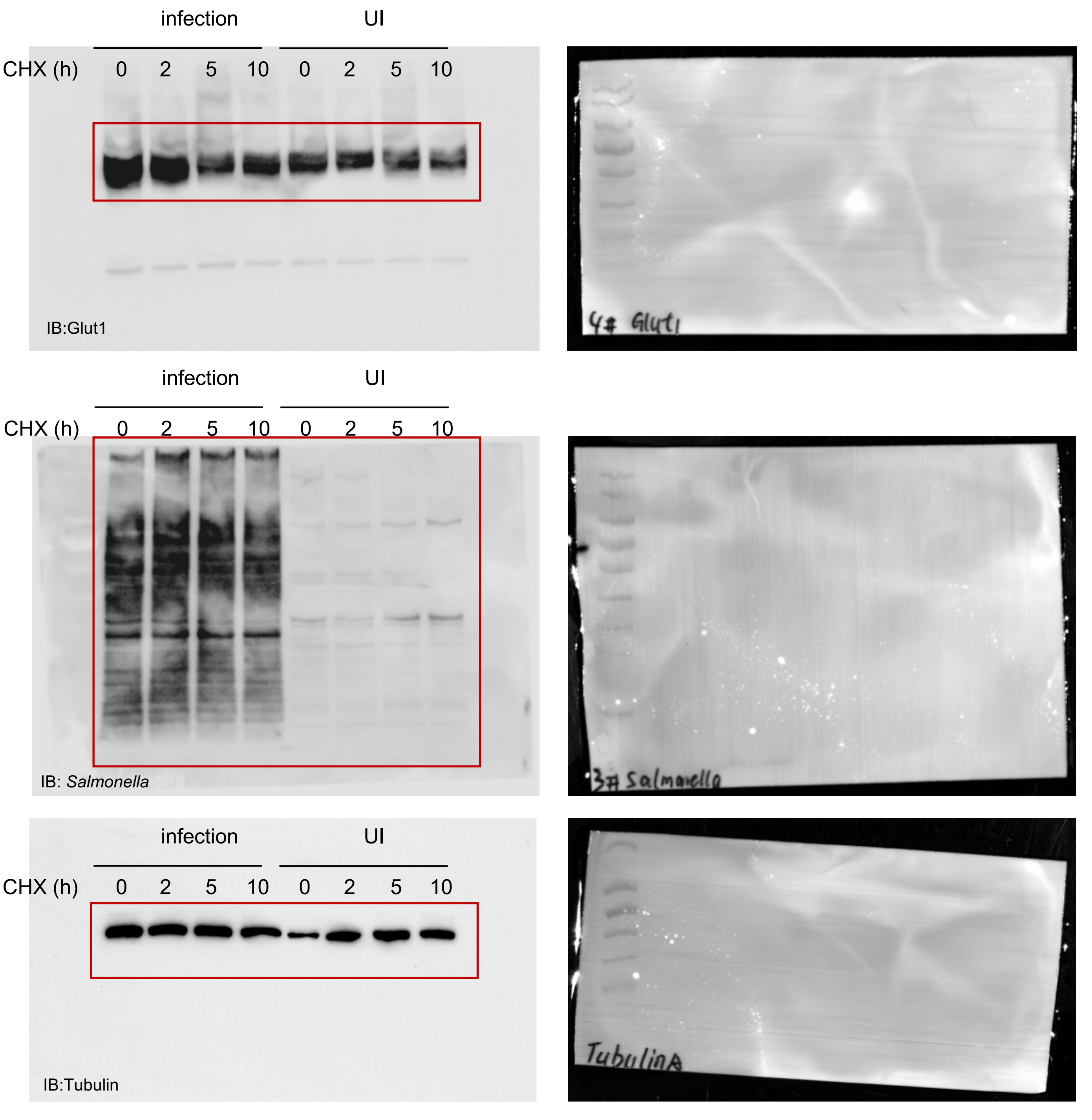

Supplement: Supplementary file 2 [file 12964_2026_2981_MOESM3_ESM.pdf]
